# miRNAs involved in the development and differentiation of fertile and sterile flowers in *Viburnum macrocephalum* f. *keteleeri*

**DOI:** 10.1186/s12864-017-4180-x

**Published:** 2017-10-13

**Authors:** Weixing Li, Zhichong He, Li Zhang, Zhaogeng Lu, Jing Xu, Jiawen Cui, Li Wang, Biao Jin

**Affiliations:** College of Horticulture and Plant Protection, Yangzhou University, Yangzhou, 225009 China

**Keywords:** Viburnum macrocephalum f. keteleeri, miRNA profiling, Fertile and sterile flowers, Flower differentiation and development

## Abstract

**Background:**

Sterile and fertile flowers are important evolutionary developmental phenotypes in angiosperm flowers. The development of floral organs, critical in angiosperm reproduction, is regulated by microRNAs (miRNAs). However, the mechanisms underpinning the miRNA regulation of the differentiation and development of sterile and fertile flowers remain unclear.

**Results:**

Here, based on investigations of the morphological differences between fertile and sterile flowers, we used high-throughput sequencing to characterize the miRNAs in the differentiated floral organs of *Viburnum macrocephalum* f. *keteleeri*. We identified 49 known miRNAs and 67 novel miRNAs by small RNA (sRNA) sequencing and bioinformatics analysis, and 17 of these known and novel miRNA precursors were validated by polymerase chain reaction (PCR) and Sanger sequencing. Furthermore, by comparing the sequencing results of two sRNA libraries, we found that 30 known and 39 novel miRNA sequences were differentially expressed, and 35 were upregulated and 34 downregulated in sterile compared with fertile flowers. Combined with their predicted targets, the potential roles of miRNAs in *V*. *macrocephalum* f. *keteleeri* flowers include involvement in floral organogenesis, cell proliferation, hormonal pathways, and stress responses. miRNA precursors and targets were further validated by quantitative real-time PCR (qRT-PCR). Specifically, miR156a-5p, miR156g, and miR156j expression levels were significantly higher in fertile flowers than in sterile flowers, while *SPL* genes displayed the opposite expression pattern. Considering that the targets of miR156 are predicted to be *SPL* genes, we propose that miR156 may be involved in the regulation of stamen development in *V*. *macrocephalum* f*. keteleeri.*

**Conclusions:**

We identified miRNAs differentially expressed between fertile and sterile flowers in *V*. *macrocephalum* f. *keteleeri* and provided new insights into the important regulatory roles of miRNAs in the differentiation and development of fertile and sterile flowers.

**Electronic supplementary material:**

The online version of this article (10.1186/s12864-017-4180-x) contains supplementary material, which is available to authorized users.

## Background

Flowers are crucial to the reproductive success and continuity of flowering plants over time [1, 2]. In general, the floral meristem sequentially produces floral organs, including the sepals, petals, stamens, and carpels, which arise in concentric rings, or whorls [3]. However, flowers come in a variety of colors, structures, shapes, and sizes, which are the evolutionary consequences of selective pressure by biotic and abiotic environmental factors [4, 5]. For example, flower size is an important characteristic shaped by insect pollinator behavior and selection, as different-sized flowers can result in different visiting frequencies, thus affecting reproductive success [6, 7]. Flowers can be classified as fertile or sterile according to their ability to reproduce sexually and produce available gametes. Fertile flowers generate normal stamens and pistils, while sterile flowers generally have abnormal reproductive structures, such as stamen, anther, and pollen abnormalities conferring sterility in males and stigma, pistil, and embryo sac abnormalities conferring sterility/infertility in females. Although sterile flowers cannot produce fruits or seeds for the next generation, they play roles in improving pollination quality by attracting pollinators and reducing the reproductive costs associated with large floral displays, consequently enhancing reproductive success [8, 9]. As a result, inflorescences composed of coexisting sterile and fertile flowers are very common in flowering plants, from grasses to trees.

Research in developmental genetics and genomics has facilitated studies of flower differentiation and development [10]. Many genes related to floral organ development have been identified. For example, the ABCDE model-related genes and MADS-box gene family are required for flower development in many angiosperm lineages [11]. MADS-box transcription factors and Zinc finger family proteins are involved in male fertile and sterile development, according to floral transcriptomic analyses [12]. Additionally, some genes, such as *TCP* and the *GRFs* family, regulate floral organ size [13].

MicroRNAs (miRNAs) are small, non-coding RNAs produced by Dicer-catalyzed excision from stem-loop precursors, which regulate gene expression at the post-transcriptional level by directing RNA cleavage or inhibiting translation of target transcripts [14]. Numerous studies have demonstrated the critical role of miRNAs in controlling flower development-related processes [15]. Some miRNAs, such as miR156 and miR172, have been found to control genes that function in flower transition [16]. Some evolutionarily conserved miRNAs can regulate the development of floral organs, including the growth and differentiation of sepals, petals, anthers, and carpels [17]. For example, miR319a is critical for petal growth and development in *Arabidopsis* through its targeting of *TCP4*, and miR159, with its target MYB, can affect anther development [18, 19]. These findings suggest that miRNAs have diverse biological functions in flower development. However, the regulatory roles of miRNAs in fertile and sterile flower differentiation and development are still unclear.

**Fig. 1:**
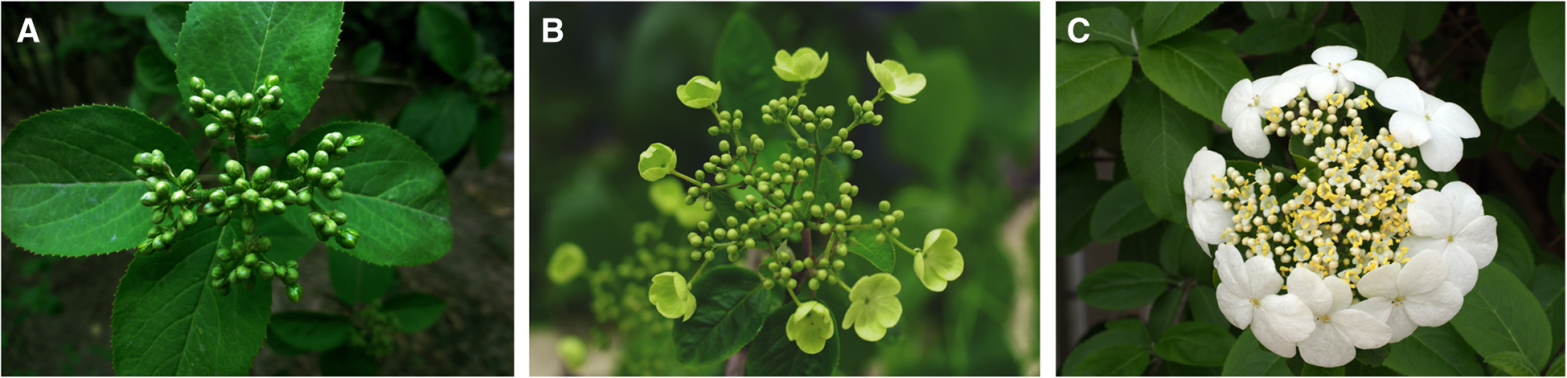
Flowers of *Viburnum macrocephalum* f*. keteleeri* at three stages. **a** Flowers in early stage (ES). **b** Flowers in middle stage (MS). **c** Flowers in late stage (LS)

*Viburnum macrocephalum* f. *keteleeri*, a Chinese wild shrub in the Adoxaceae family, has two kinds of flowers in an inflorescence [8]. The type distributed in the internal position of the inflorescence is fertile, whereas the sterile flowers are distributed around the exterior (Fig. 1). Fertile flowers are morphologically smaller than sterile flowers, but the number of fertile flowers is much greater than that of sterile flowers. In particular, these two kinds of flowers are produced from a single inflorescence, and the sterile flowers evolve from flowers that were initially fertile [8]. Therefore, *V*. *macrocephalum* f. *keteleeri* is an ideal candidate species for investigating the differentiation of fertile and sterile flowers in the same genetic background. To determine the miRNAs regulating the development of fertile and sterile flowers, we used high-throughput sequencing and detected more than 11 million reads for each type of flower. We identified known and novel miRNAs in the flower organs of this species and investigated the targets of these miRNAs to explore the potential functions of the miRNAs. We analyzed the dynamic expression patterns of the miRNAs and their putative targets using our transcriptomic data (NCBI SRA [SRP076665] and GEO [GSE83429]). Quantitative real-time polymerase chain reaction (qRT-PCR) was used to validate the dynamic expression patterns of the miRNAs and their putative targets. The results of this study contribute to our understanding of the regulatory roles of miRNAs in the development of fertile and sterile flowers.

## Methods

### Plant materials

We used adult *V*. *macrocephalum* f. *keteleeri* plants grown at Yangzhou University, Jiangsu, Eastern China (32°39′N, 119°43′E). We observed and digitally recorded flowers at different developmental stages every week from March to May. Fertile and sterile flowers were collected from three phases of floral development: early (ES; Fig. 1a), middle (MS; Fig. 1b), and later (LS; Fig. 1c) stages of flower induction and the flowering process. Sufficient samples were immediately frozen in liquid nitrogen and stored at −80 °C for RNA extraction, and some fresh tissues were also collected for morphological observations. Fertile and sterile flowers from the MS (the period of rapid differential growth; Fig. 1b) were selected for the construction of small RNA (sRNA) libraries.

### Morphological observations of flower petals

For scanning electron microscope observation, flower petals collected at different stages were fixed in 2.5% glutaraldehyde (in 0.1 M phosphate buffer, pH 7.2) for 2 h at 4 °C. They were then rinsed three times in 0.1 M phosphate buffer (15 min per rinse), dehydrated in a graded ethanol series (30, 50, 70, 80, 90, 95, and 100%; 15 min each), and dried by automatic critical point drying (Leica, Wetzlar, Germany). After sputter coating (Bal-Tec, Balzers, Switzerland), the samples were examined, imaged, and measured by scanning electron microscopy (SEM) (Hitachi High-Technologies Corporation, Tokyo, Japan) at 15.0 kV [20]. Petal cell size measurements under SEM were based on three samples selected randomly at each stage. Averages and standard deviations were calculated using Microsoft Office Excel 2003. The surface area, size, and number of petal cells were calculated as follows: 1) Image J (1.48) software was used to measure the surface areas of the flower petals. 2) Measurements of petal cell size using SEM were conducted in three samples selected randomly at each stage of floral development. More than 10 different positions on each petal sample were photographed; thus, at least 30 replicates in each fertile and sterile flower were used to calculate the average cell size. 3) Using the results from 1) and 2), we estimated the number of surface cells on flower petals at each floral stage as the surface area of the flower petal (the result from 1) divided by the petal cell size (the result from 2).

For semi-thin section observations, the sterile and fertile flowers were separately cut from buds to full-bloom stage flowers using a razor blade. Each sample was pre-fixed in 2.5% (*v*/v) glutaraldehyde (in 0.1 mol/L phosphate buffer, pH 7.2) at 4 °C for 2 h. The samples were washed three times in 0.1 M phosphate buffer (pH 7.2, 10 min per rinse), dehydrated in an ethanol series (30, 50, 70, 80, 90, and 95%; 30 min for each step), and then washed three times for 30 min with 100% ethanol. Then, the ethanol/propylene oxide ratios were adjusted to 7:3, 5:5, and 3:7 by adding the oxide proportion. After the samples were treated three times with pure propylene oxide (10 min each rinse), the samples were infiltrated gradually with spurr resin [21]. Then they were agitated in an oscillator for 12 h. Finally, resin masses were acquired by oven drying. For ultrastructural observations, 70-nm thick sections were cut with a Leica EM UC6 ultramicrotome (Leica) and stained with Toluidine blue. The cells were observed and photographed under the Primo Star biological microscope (ZEISS, Oberkochen, Germany).

### sRNA sequencing

Two sRNA libraries, from fertile and sterile flowers, were constructed and sequenced by Novogene Biotechnology Corporation (Beijing, China) using the Illumina Genome Analyzer. Total RNA was isolated from *V*. *macrocephalum* f. *keteleeri* sterile flowers (VMS) and fertile flowers (VMF) using the Mini BEST Plant RNA Extraction Kit (TaKaRa, Dalian, China) and treated with genomic DNA (gDNA) Eraser (TaKaRa) to eliminate any DNA contamination. RNA degradation, contamination, purity, and integrity were all measured to ensure high quality. RNA degradation and contamination were monitored on 1% agarose gels. RNA purity was assessed using the NanoPhotometer® spectrophotometer (IMPLEN, CA, USA), and the RNA concentration was measured using the Qubit® RNA Assay Kit and the Qubit® 2.0 Fluorometer (Life Technologies, CA, USA). RNA integrity was assessed using the RNA Nano 6000 Assay Kit of the Agilent Bioanalyzer 2100 system (Agilent Technologies, CA, USA). Sequencing libraries were generated using the NEBNext® Multiplex Small RNA Library Prep Set for Illumina® (New England Biolabs, Inc., Ipswich, MA, USA) following the manufacturer’s recommendations, and index codes were added to attribute sequences to each sample.

### Identification and prediction of known and novel miRNAs

After quality control and removing tags from these sources, mapped sRNA tags were used to look for known and novel miRNAs. The sRNA annotation process was performed as follows: 1) The sRNA tags were first mapped to the reference sequence (our transcriptome data sets NCBI SRA [SRP076665] and GEO [GSE83429]) using Bowtie [22, 23], without allowing any mismatches, to analyze their expression and distribution relative to the reference sequence. 2) Next, the mapped sRNA tags were used to search for known miRNAs using a modification of the miRDeep2 program (with miRBase used as the reference). 3) The mapped sRNA tags were also mapped to Rfam, RepeatMasker, to annotate the tags and remove those originating from protein-coding genes, repeat sequences, ribosomal RNAs (rRNAs), transfer RNAs (tRNAs), small nuclear RNAs (snRNAs), and small nucleolar RNAs (snoRNAs). 4) The remaining unannotated tags were used to predict novel miRNAs by exploring secondary structures, Dicer cleavage sites, and minimum free energies using the miREvo and miRDeep2 software packages [24, 25]. Novo Custom scripts (Beijing Novo Gene Genomics Institute, China) were used to obtain the miRNA counts; they were also used to obtain the base bias at the first position of the identified miRNAs of certain lengths and, subsequently, at each position of all identified miRNAs.

### Expression analysis of miRNAs and prediction of miRNA targets

MiRNA expression levels were estimated as transcripts per million (TPM) using the following calculation: normalized expression = mapped read count/total mapped reads × 1,000,000. Differential expression analysis of two flowering phases was performed using the DESeq R package (1.8.3). miRNAs that had change ratios of greater than 2 or less than 0.5 (Fold change Log2 > 1 or < −1) and *p* < 0.01 were set as the default thresholds for significant differential expression [26].

We used the web-based psRNATarget program (http://plantgrn.noble.org/psRNATarget/) and psRobot_tar in psRobot to identify putative targets for known and novel miRNAs [27]. We used psRobot software to evaluate all predicted target genes using a previously defined scoring system. Genes with a score < 3 were considered miRNA targets [28].

### Cloning and sequencing of pre-miRNA sequences

Total RNA was isolated from VMS and VMF at MS as described above. cDNAs were synthesized from 2 μg of purified total RNA in 40 μl reactions using the PrimeScript™ 1st Strand cDNA Synthesis Kit (TaKaRa) according to the manufacturer’s protocol. We designed 28 pairs of primers for *V*. *macrocephalum* f. *keteleeri* precursor miRNA sequences (Additional file 1). PCR amplifications were carried out, using the following thermal cycling conditions: 94 °C for 5 min, 35 cycles at 94 °C for 30 s, 55 °C or 60 °C for 15 s, and 72 °C for 50 s. Amplification products were separated on a 2% agarose gel with GelRed nucleic acid staining. Gel-purified PCR fragments were subcloned into the T5-simple Vector system (TransGen, Beijing, China) and sequenced.

### qRT-PCR validation of miRNAs and their target genes

The RNA solution (10 μl), after removal of DNA contamination, was subjected to reverse transcriptase reactions with PrimeScript™ Reverse Transcriptase Reagent Kit with gDNA Eraser (Perfect Real Time) (TaKaRa) in accordance with the manufacturer’s protocol. Specific primers for 35 miRNAs and 6 target genes were designed using Primer Premier 5.0 (Additional file 1). qRT-PCR was performed using the SYBR Green PCR Master Mix (TaKaRa) on a CFX96 Detection System (Bio-Rad, Hercules, CA, USA). Briefly, the 25 μl PCR reaction contained no more than 100 ng cDNA, 12.5 μl SYBR Premix Dimer Eraser (2×), and 0.3 μM of each primer. The reactions were mixed and incubated at 95 °C for 30 s, followed by 40 cycles of 95 °C for 5 s, 55 °C for 30 s, and 72 °C for 30 s. The expression levels of the miRNAs and target genes were normalized to those of the internal controls U6 [29] and actin (NCBI, XM_002282480) respectively. The relative expression levels were analyzed using the 2^-ΔΔCT^ method [30]. Ct represents the threshold cycle.

## Results

### Morphological and structural observations of floral organs

**Fig. 2:**
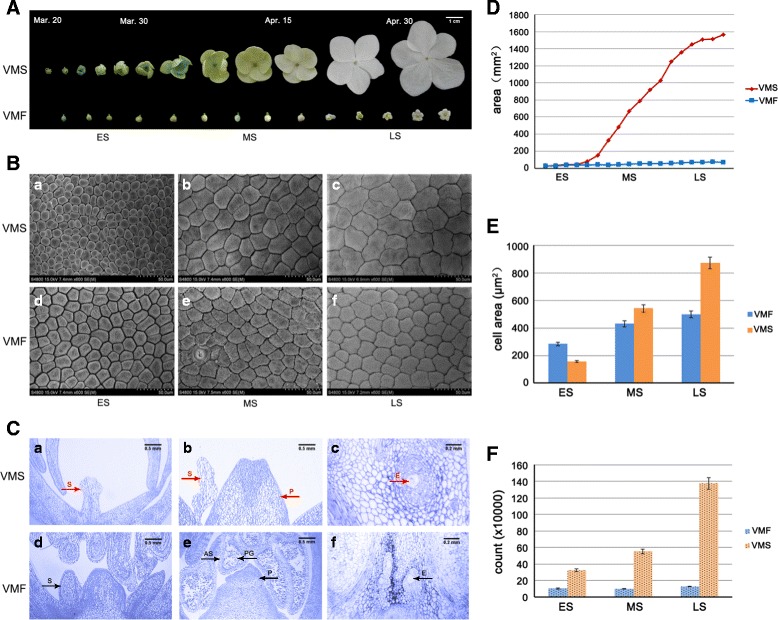
Morphological and structural observations of floral organs. **a** Fertile (VMF) and sterile (VMS) flowers of *V*. *macrocephalum* f. *keteleeri* observed by digital camera from early to late April. Scale bar: 1 cm. **b** Cellular morphology of VMF (**d**–**e**) and VMS (**a**–**c**) at three different developmental stages. Scale bars: 50 μm. **c** Anatomical features of VMF (**d**–**e**) and VMS (**a**–**c**). Red arrows indicate the short stamens (**a**), abnormal stamens (**b**), and the abnormal embryo sac with cavity in the ovary (**c**) of VMS. Black arrows indicate the normal stamens (**d**), stamens with anthers (**e**), and normal embryo sacs (**f**) of VMF. S: stamen, E: embryo sac, P: pistil, PG: pollen grain, AS: anther septa. Scale bars in a, b, d, and e: 0.5 mm; scale bars in c and f: 0.2 mm. **d** Petal areas of VMF and VMS. **e** Cell counts of VMF and VMS. **f** Cell areas of VMF and VMS

Flowers were observed from late March to late April with respect to the differentiation and development into fertile and sterile flowers. The morphological changes of fertile and sterile flowers at different stages were compared by recording images with a digital camera. In the ES (March 20–30), fertile and sterile flower petals were green; in the MS (March 30 to April 15), the color gradually changed from green to white; and, at the LS (April 15–30), the color was completely white (Fig. 2a). Obviously, the petal area of sterile flowers increased significantly during the developmental process, and it did so to a much greater extent than did that of the fertile flowers. For example, in the ES, the petal areas of fertile and sterile flowers were similar, about 30 mm^2^. However, after the ES, sterile flowers expanded rapidly; in the MS, the petal area of the sterile flowers increased from 325.331 mm^2^ to 1028.181 mm^2^, whereas the fertile flower petal area increased by only 14 mm^2^. At the LS, the petal area of sterile flowers reached about 1600 mm^2^ and was more than 20 times that of fertile flowers (Fig. 2d).

SEM photographs displayed the cellular morphology of fertile and sterile petals in the ES, MS, and LS (Fig. 2b). In the ES, petal cells were loosely packed (Fig. 2b, a and d). The flat surface area of a sterile flower petal cell is about 154 μm^2^, and that of fertile petals is about 283 μm^2^ (Fig. 2e). In the MS, cells of both flowers were arranged closely, and the intercellular space was small (Fig. 2b–e). The cellular area of sterile flowers was a little more than that of fertile flowers, at 542 μm^2^ and 430 μm^2^, respectively (Fig. 2e). In the LS, cells were arranged compactly (Fig. 2b–f). The cellular area of sterile flowers increased to 873 μm^2^, with many dividing cells, about twice the size of fertile flowers (Fig. 2e). In addition to the petal area, we also estimated the number of cells on the petal surfaces of fertile and sterile flowers at each stage. The cell count increased from 30 × 10^4^ to 140 × 10^4^ from the ES to LS in sterile flowers, whereas it remained constant, at approximately 10 × 10^4^, in fertile flowers (Fig. 2f).

The anatomical features of VMS and VMF are shown in Fig. 2c. There were no stamens, or only thin and short stamens, in VMS (Fig. 2c, a and b). In contrast, the stamens of VMF had long filaments and normal anthers with pollen grains and anther septa (Fig. 2c–e). Additionally, relative to the normal pistils of fertile flowers, the pistils in sterile flowers lacked normal embryo sacs (Fig. 2c–f). These results indicate that sterile flowers have remarkably low fertility, with degraded stamens and pistils, whereas the stamens and pistils are normal, displaying healthy fertility, in fertile flowers of *V*. *macrocephalum* f. *keteleeri.*

### An overview of high-throughput sequencing datasets in fertile and sterile flowers

To obtain a comprehensive profile of the sRNAs involved in *V*. *macrocephalum* flowers, two sRNA libraries (VMF and VMS) were constructed. A total of 11,447,459 and 12,365,813 raw sRNA reads were generated, respectively (Table 1). After removing low-quality reads, adapter sequences, poly N sequences, sequences of <18 nucleotides (nt), and other artifacts, 7,562,443 and 7,373,905 high-quality clean reads between 18 nt and 30 nt in length, respectively, remained for further analyses (Additional file 2).

**Table 1:** Summary of sRNA sequencing statistics

Type	VMF Count (percent)	VMS Count (percent)
Total reads	11,447,459 (100.00%)	12,365,813 (100.00%)
N% > 10%	0 (0.00%)	2 (0.00%)
low quality	7268 (0.06%)	6973 (0.06%)
3_adapter_null or insert_null	241,136 (2.11%)	491,043 (3.97%)
5_adapter_contamine	36,161 (0.32%)	36,161 (0.32%)
with poly A/T/G/C	30,903 (0.27%)	43,226 (0.35%)
Clean reads	11,131,991 (97.24%)	11,803,832 (95.46%)

**Fig. 3:**
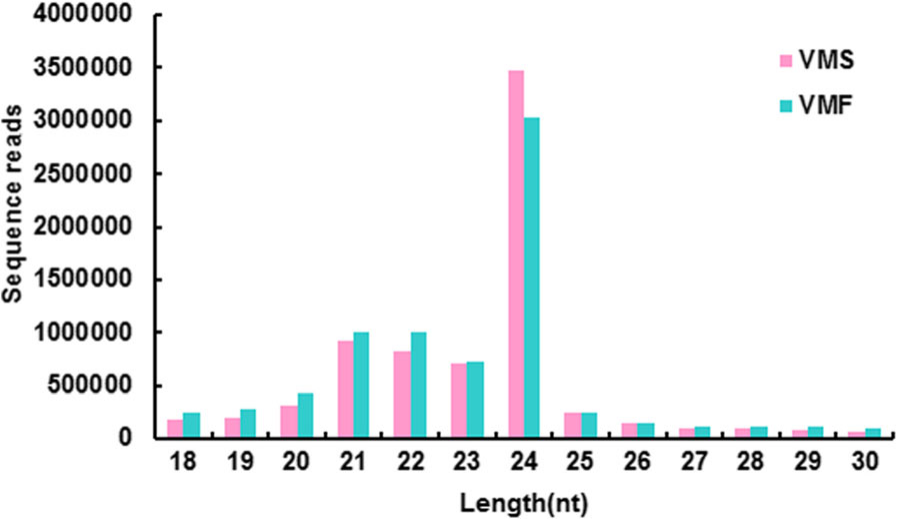
Length distribution of small RNAs (sRNAs) from sterile and fertile flowers

We then summarized the length distribution of the sRNA libraries of fertile and sterile flowers. The size distribution of sequenced sRNAs was similar in both samples. The majority of clean sRNA reads were 21–24 nt in both libraries. The 24 nt sRNAs were the most abundant, at approximately 3,028,365 (40.04%) and 3,480,318 (47.20%) in fertile and sterile flower libraries, respectively (Fig. 3). Additionally, 21, 22, and 23 nt sRNAs were more common than those of any other length besides 24 nt.

A total of 3,327,774 (44%) and 2,811,600 (38.13%) reads from fertile and sterile flowers, respectively, were mapped to non-coding sRNA database (Additional file 3). The mapped sRNA sequences were clustered into several RNA classes, such as known miRNA, rRNA, tRNA, snRNA, snoRNA, repeats, and TAS (Additional file 4). Most of these sRNAs (89.85% and 90.40%, respectively) were clustered in the uncharacterized group. The second most abundant group (5.46% and 4.76%, respectively) was rRNA. 37,648 and 47,191 sequences were known miRNAs, at 1.13% and 1.68% in VMF and VMS, respectively. Additionally, 20,197 and 24,478 of the unmapped sRNA sequences were identified as novel miRNA candidates in VMF and VMS, respectively (Table 2).

**Table 2:** Summary of mapped mature and hairpin structures in known and novel miRNAs

Types	Known			Novel		
	Total	VMF	VMS	Total	VMF	VMS
Mapped total sRNA	84,839	37,648	47,191	44,675	20,197	24,478
Mapped uniq sRNA	425	226	199	1027	514	513
Mapped hairpin	61	54	56	73	71	70
Mapped mature	49	42	45	67	59	64

### Identification of known and novel miRNAs

We searched our results against miRBase 21.0 to annotate known miRNAs in *V*. *macrocephalum* f. *keteleeri*. Ultimately, we identified 226 and 199 unique sRNAs among the known miRNAs and 514 and 513 sRNAs among the novel miRNAs in fertile and sterile flowers, respectively (Table 2). After screening, 49 mature known miRNAs were identified. All precursors of mature miRNAs can adopt hairpin structures resembling the fold-back structure of a miRNA precursor (Additional file 5). Analysis of nucleotide bias was performed on known miRNAs. The analysis showed that the first position of miRNAs of 18 to 24 nt in length was occupied by uracil (U) over 80% of the time (Additional file 6). At the first and second positions, the percentage of U averaged 97.73% and 80.80%, respectively. The percentage of cytosine (C) was the most increased at the last several base pair positions of the miRNAs compared with at the front positions.

**Fig. 4:**
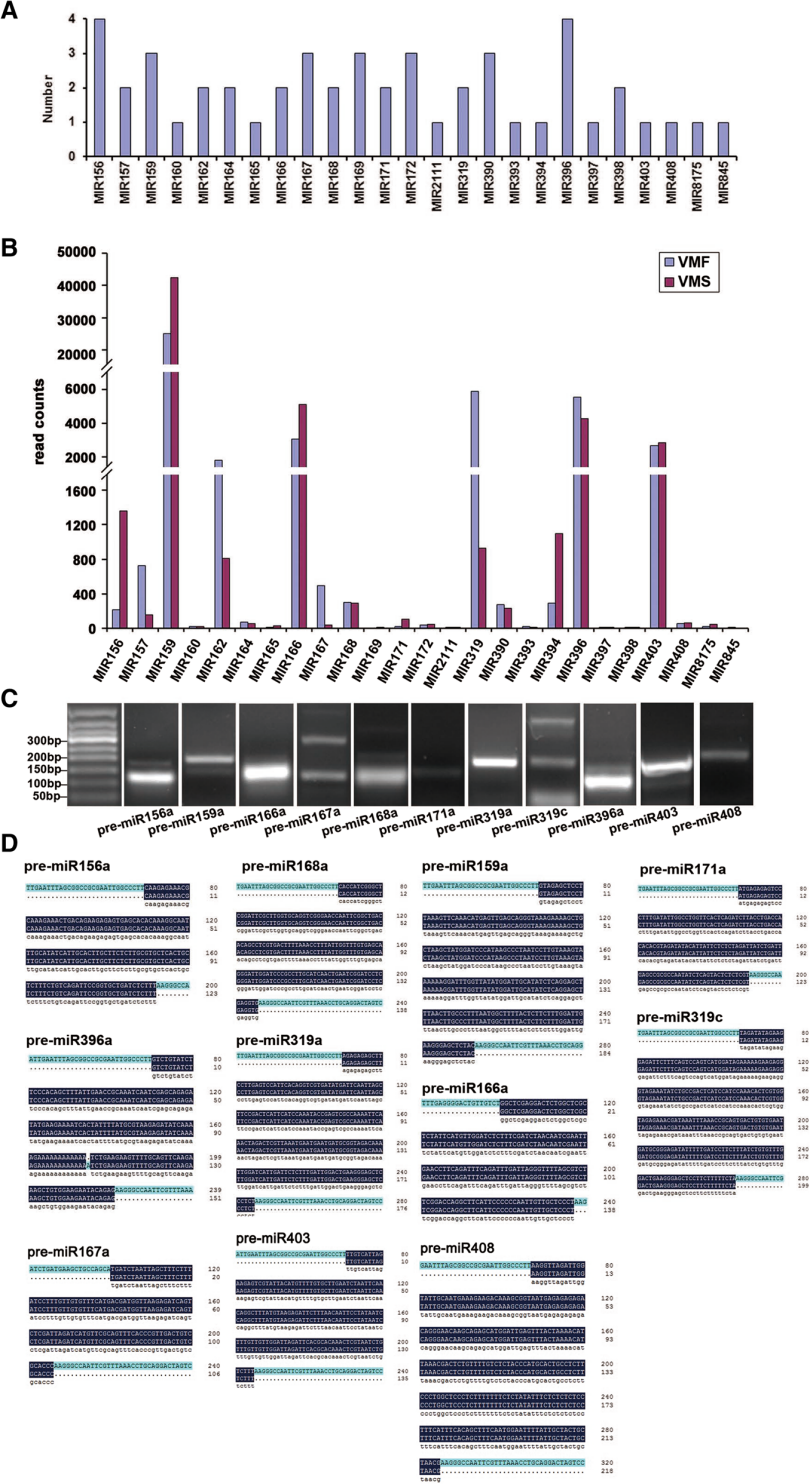
Known microRNAs (miRNAs) in fertile and sterile flowers. **a** Numbers of members identified in the 25 conserved miRNA families. **b** Read counts of each known miRNA family. **c** Pre-miRNA PCR results of some known miRNAs under UV transillumination. **d** Sequence alignment results and Illumina sequencing of known miRNAs

A total of 24 miRNA families were identified among these known miRNAs (Fig. 4a). The largest family was miR156 and miR396, with four members, followed by miR159, miR167, miR169, miR172, and miR390, with three members; however, most miRNA families possessed only one family member (e.g., miR160, miR165, miR408). The abundance of known miRNAs varied greatly. miR159 had 25,054 and 42,454 reads in VMF and VMS, respectively, and was the most abundant. miR162, miR166, miR319, miR396, and miR403 all had abundance reads of more than a thousand, whereas many miRNAs (e.g., miR169, miR2111, miR397, miR398) were sequenced too infrequently to be distinguishable in the histogram (Fig. 4b; Additional file 7).

**Fig. 5:**
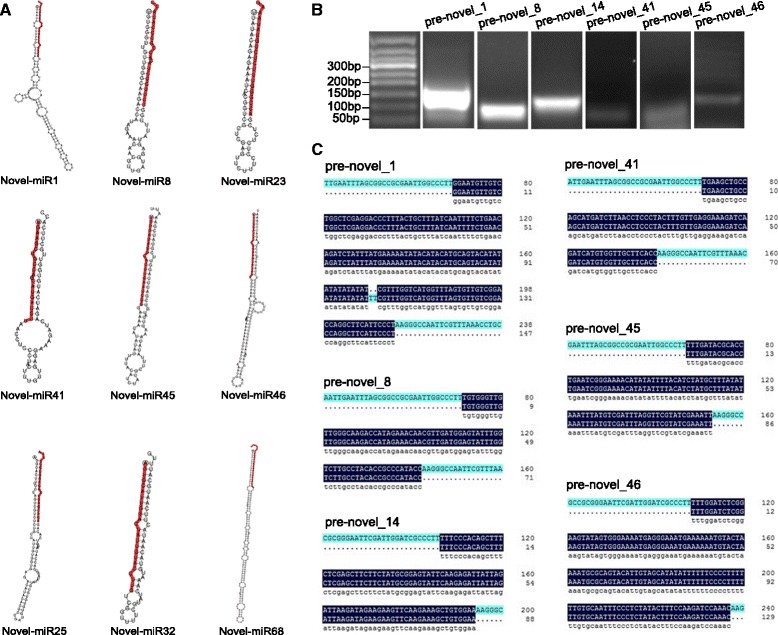
Novel miRNAs in fertile and sterile flowers*.* **a** Examples of stem-loop hairpin secondary structures of predicted new miRNA precursors. Segments corresponding to the mature miRNAs are shown in red. **b** Pre-miRNA PCR results of some novel miRNAs under UV transillumination. **c** Sequence alignment and Illumina sequencing of novel miRNAs

In addition to the known miRNAs, we identified novel miRNAs in flowers. According to the criteria for annotating novel miRNAs, a characteristic stem-loop precursor is a prerequisite [31]. A total of 73 novel miRNA precursors were predicted from 1027 unique sRNAs using miREvo and miRDeep2. The length of novel miRNA precursors ranged from 38 to 300 nt, with an average of 116 nt, and the MFE values obtained for these precursors ranged from −12 kcal/mol to −101.5 kcal/mol, with an average of −46.8 kcal/mol (Additional file 8). A total of 67 candidate novel miRNAs with clear precursors containing a stem-loop secondary structure were identified (Fig. 5a; Additional file 8). The complementary miRNA* sequences for each candidate novel miRNA were also detected, although most were present at lower expression levels than their corresponding miRNAs (Additional file 9).

At the same time, custom scripts were used to analyze the nucleotide bias at the first position of miRNAs of certain lengths; this information was subsequently obtained for each position of all identified miRNAs. The nucleotide bias analysis revealed that U appeared at the beginning of the miRNAs with a high frequency, 78.34% on average (Additional file 10). In novel miRNAs, the expression levels of each mature miRNA were obviously different. The expression of novel_1, 10, 4, and 6 were all above 1000 in VMS and VMF, whereas the expression levels of many novel miRNAs (e.g., novel_100, 102, 103, 105, 55, 57, and 59) were less than 10 (Additional file 11).

The precursor sequences of the 28 known and novel miRNA candidates were further validated by sub-cloning techniques (PCR amplification and Sanger sequencing). Of the 28 sequences validated by Sanger sequencing, 15 were completely identical to hairpin sequences, whereas 2 had fewer than three mismatched nucleotides (Fig. 4c and Fig. 5b). Of the known miRNAs, the pre-miRNA sequences of miR156a, miR159a, miR165a, miR167a, miR319a, and miR171a were perfectly matched with the statistics in the library, and miR396a had one mismatched nucleotide (Fig. 4d). Of the novel miRNAs, the pre-miRNA sequences of novel_8, 14, 41, 45, and 46 were identical to the sequences obtained from sRNA sequencing, and novel_1 contained two mismatched nucleotides (Fig. 5c).

### Analysis of miRNAs and their targets in fertile and sterile flowers

To further understand the functions of known and novel miRNAs, we predicted their putative targets by employing the web-based software psRNATarget and psRobot_tar in psRobot, with default parameters. We found 630 miRNA-target pairs for known miRNAs and 1209 for novel miRNAs (Additional files 12 and 13). The number of predicted targets varied from 1 to 141 per miRNA, and miRNAs of the same family had similar targets. A total of 190 unigene sequences were predicted to be the targets of 49 known miRNAs, and 384 unigene sequences were predicted to be the targets of 67 novel miRNAs (Additional files 14 and 15).

These target genes belong to several gene families predicted to play roles in a broad range of physiological processes. Of these targets, nine SBP-box genes were potentially regulated by miR156 and miR157 family members; four MYB genes were potentially regulated by novel_83, novel_87, and novel_112, and one *AP2* gene was potentially regulated by miR172 and novel_50, respectively, suggesting complex and specific regulation of flower development by miRNAs. Four and three genes annotated as *GRAS* and *ARF* genes, respectively, which are closely associated with plant growth, were identified among the miRNA targets. Other putative gene families identified included NAC, bHLH, and CAMTA. Other than genes known to be members of transcription factor families, many target genes were annotated with their assumed functions associated with cellular components and biological processes. Additionally, a small proportion of the putative target genes had no known functions or significant similarities to other genes in the databases, implying the existence of unknown and specific flower development regulatory pathways.

**Fig. 6:**
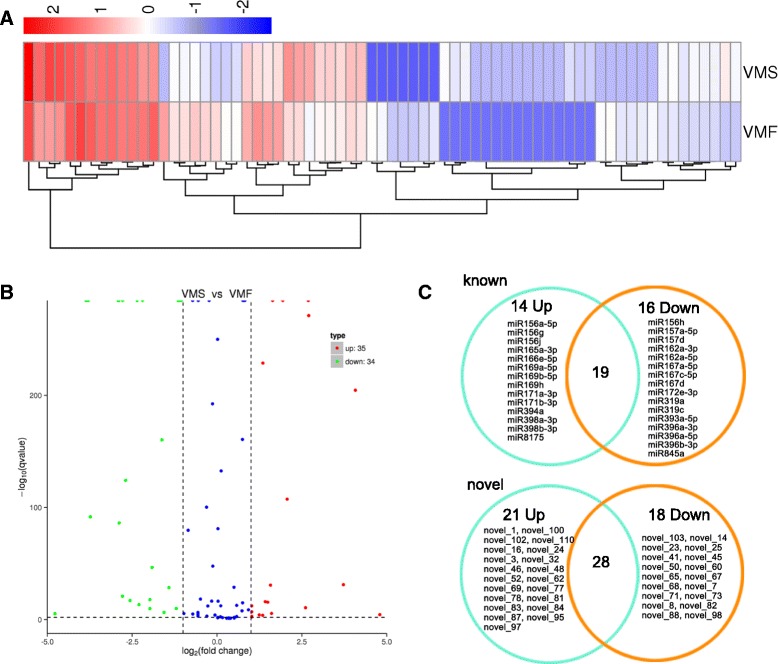
Differentially expressed miRNAs between VMF and VMS. **a** Cluster analysis of differentially expressed sRNA. Red represents highly expressed miRNAs. Blue represents slightly expressed miRNAs. **b** Scatter diagram of the differential read counts of miRNAs. Red and green points represent miRNAs that have significant differences when their *p* < 0.01 and log2(foldchange)| > 1. Blue points represent miRNAs with no significant expression differences. **c** Known and novel miRNAs up- and downregulated in VMS vs. VMF

To investigate the miRNAs involved in flower development, we compared normalized miRNA levels between VMF and VMS (Fig. 6a). miRNAs that had fold change log2 > 1 or < −1, and *p* < 0.01, were considered to be differentially expressed. Among these, 30 known and 39 novel miRNA sequences were differentially expressed (Fig. 6b, Additional file 16). Compared with the comparable data for fertile flowers, in sterile flowers, 14 known miRNAs were upregulated and 16 were downregulated, whereas 21 novel miRNAs were upregulated and 18 were downregulated (Fig. 6c).

**Fig. 7:**
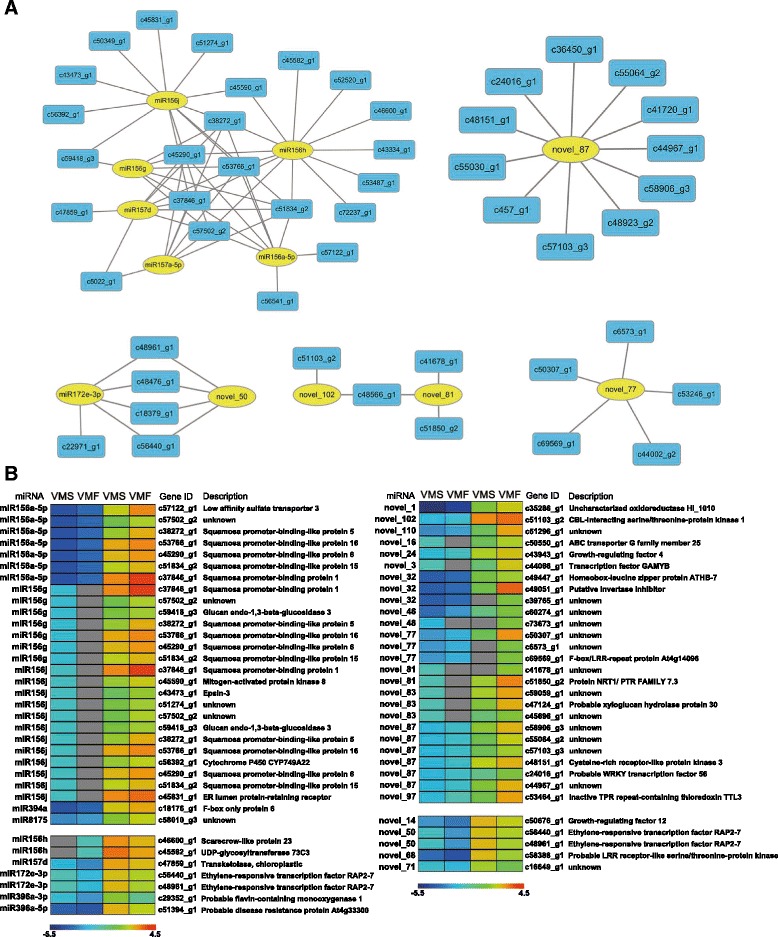
Network and heat maps of miRNAs and their targets. **a** Network of miRNAs (yellow) and their targets (blue). **b** A combined view of the inverse correlation between the expression of miRNAs and that of their target genes in VMS and VMF. The expression of miRNAs (left) in VMS and VMF was determined by sRNA sequencing. The miRNA targets (right) were predicted by computational methods, and their expression was determined by RNA sequencing. The bar represents the expression scale for the miRNAs (−log10 transcripts per million) and target genes (log10 transcripts per million) in VMS and VMF. Higher miRNA expression levels are indicated by dark blue and higher target gene expression levels by dark red. Non-expressed miRNAs are shown in grey

Along with the transcriptome, we also constructed a network of miRNAs that regulated mRNA based on differentially expressed miRNA and targets (Fig. 7a). Novel_87 targeted 11 differentially expressed genes, implying that novel miRNAs may play regulatory roles. Additionally, novel_50 and miR172e-5p were found to target the same genes (Fig. 7a).

Furthermore, we compared the expression profiles of the differentially expressed miRNAs and target genes (Additional files 17 and 18). A heat map showed that the expression levels of most of the known and novel miRNAs were negatively correlated with those of their target genes, which is consistent with the gene-silencing function of miRNAs (Fig. 7b). Although the functions of half the novel miRNA targets are unknown, we found that some individual novel miRNAs may target important transcription factors (Fig. 7b; Additional file 19). For example, c50550_g1 was described as ABC transporter G family member 25, which is involved in the intercellular ABA signaling pathway in *Arabidopsis thaliana* [32]. Additionally, c43943_g1 was described as *GRF4*, which plays a role in the regulation of cell expansion in leaf and cotyledon tissues in *A. thaliana* [33]. However, the expression of only about 20% of target genes were positively correlated with their corresponding known miRNAs and novel miRNAs enriched in flowers. We cannot rule out the possibility that these miRNAs are not responsible for the differential expression patterns of their target genes, because gene expression can be influenced by many factors. These data indicated the difference between the expression profiles of known and novel miRNAs and their targets in *V*. *macrocephalum* f. *keteleeri* flowers.

### Expression of miR156 and its targets

We particularly focused on miR156 family members, based on their expression in the network and the heat map. In the network of miRNA and targets, miR156 family members (miR156a-5p, miR156h, miR156g, and miR156j) with miR157 family members (miR157a-5p, miR157d) regulated similar potential targets (c106171_g1, c107764_g1, c11862_g1, c30688_g1, c32903_g1, c33287_g2, etc.) (Fig. 7a).

In addition, we found the expression of some miR156 family members (miR156a-5p, miR156g, miR156j) to be the opposite of the expression of the SBP family genes (*SPL1*, *SPL5*, *SPL6*, *SPL15*, *SPL16*), which is involved in flower transition and late development (Fig. 7b). Based on the expression of 1352 and 213 reads in VMS and VMF, miR156a-5p showed obvious expression differences. miR156g and miR156j were expressed only in VMS. The expression levels of their potential targets *SPL1*, *SPL5*, *SPL6*, *SPL15*, and *SPL16* were greater in VMF than in VMS.

### qRT-PCR validation of the miRNAs

**Fig. 8:**
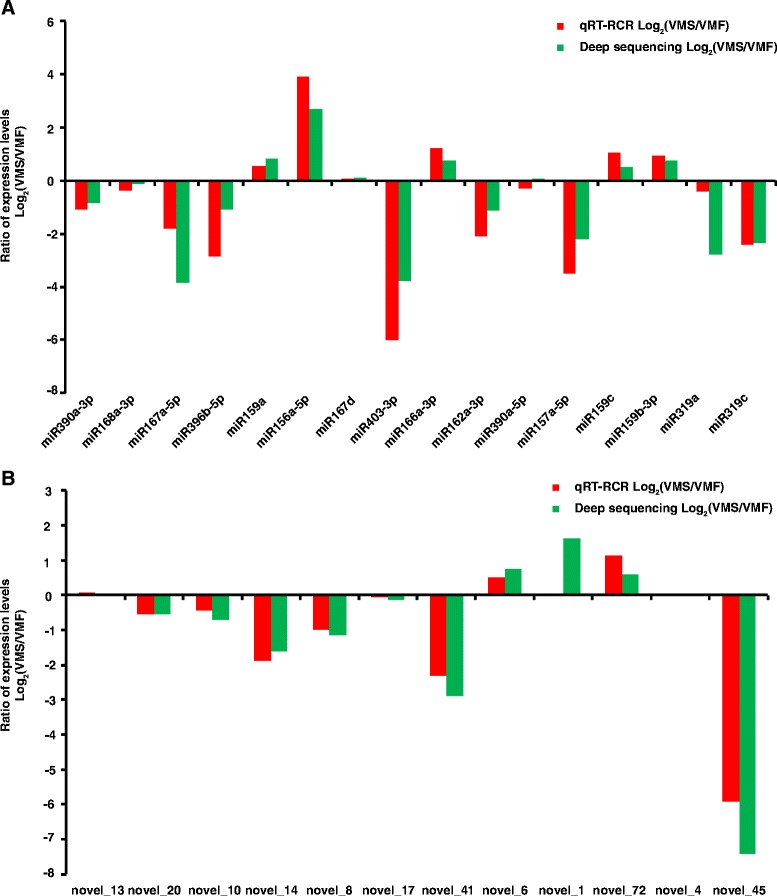
The expression ratios [log2 (VMS/VMF)] of miRNAs (known [**a**] and novel [**b**]) in *V. macrocephalum* f. *keteleeri,* as determined by qRT-PCR and deep sequencing

To validate the dynamic expression patterns of the miRNAs, we analyzed the expression patterns of 20 known miRNAs and 15 novel miRNAs with known precursor sequences by qRT-PCR at the MS of flower development (Fig. 8). The relative transcript levels of 16 known miRNAs (Fig. 8a) and 12 novel miRNAs (Fig. 8b) were similar to those observed in the sequencing results (Fig. 8a, b). However, the relative transcript levels of four known miRNAs and three novel miRNAs at this stage were different from those in the sequencing results (Additional file 20). Potential explanations for these inconsistent miRNA expression results include differences in the preparation of the library versus validation samples and the lack of replicates in the sequencing library.

**Fig. 9:**
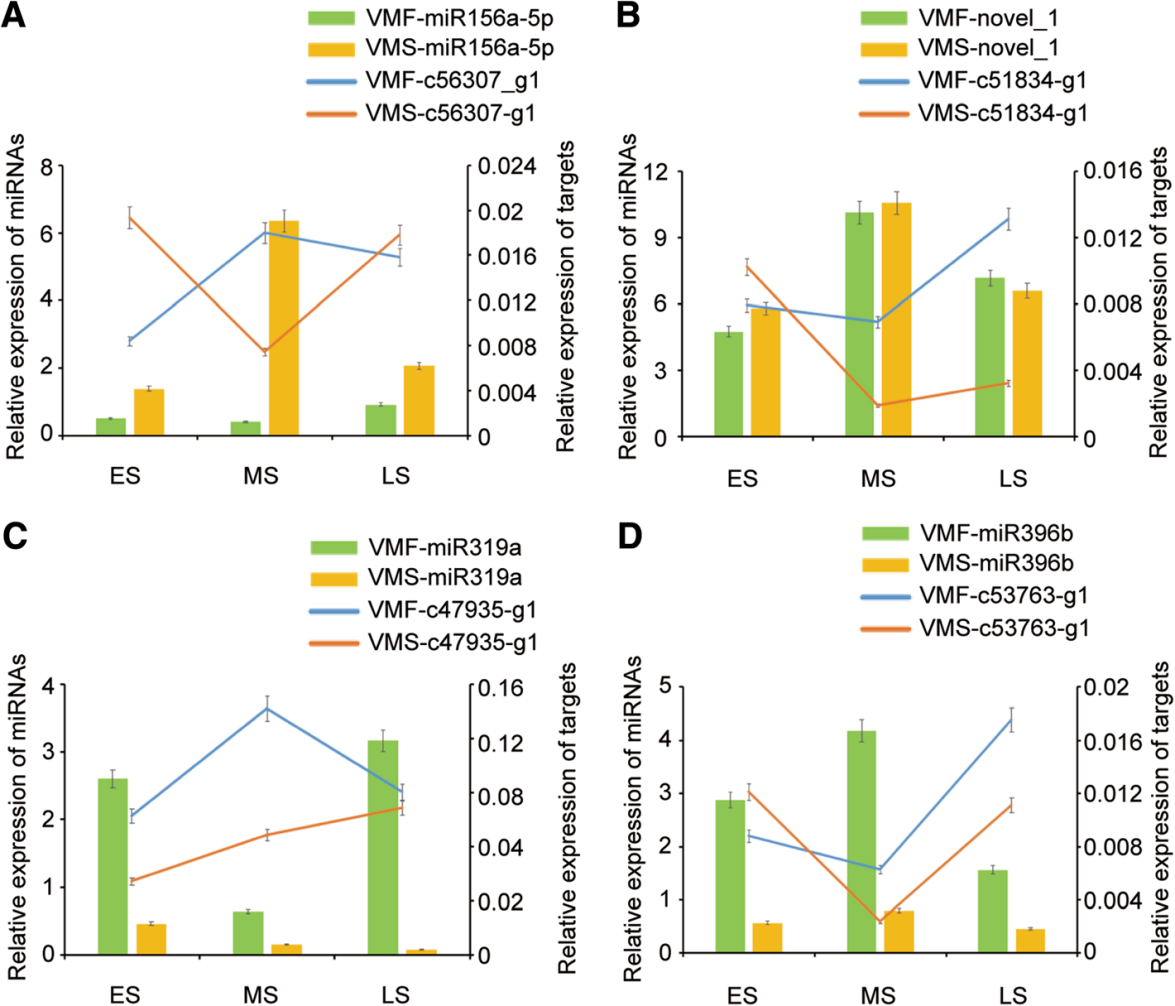
Correlations between miRNA and target gene expression. **a-d** The bars and lines indicate the expression levels of miRNAs and their corresponding targets, respectively, at the three different floral growth stages, as determined by qRT-PCR. The left and right y-axes represent the expression levels of the miRNAs and target genes, respectively. The error bars indicate the standard deviations

To assess the influence of the miRNAs on their putative targets, we analyzed the expression correlations between miRNAs and their identified targets during three different floral growth stages (Additional file 21). Six interesting miRNA/target modules were identified by qRT-PCR analysis (Fig. 9, Additional file 22). A comparison of the expression levels observed at ES with those at MS revealed negative relationships of miR156a-5p and novel_1 with their corresponding gene targets (Fig. 9a, b). The expression level of c47935-g1 gradually increased, while that of the corresponding miR319a decreased, from the ES to LS in VMS (Fig. 9c). In addition, while the expression of miR396b decreased dramatically, that of the target c53763-g1 increased, from the MS to LS in VMF (Fig. 9d).

## Discussion

### Complex sRNA populations involved in the differentiation and development of fertile and sterile flowers

During the life cycle of flowering plants, flower development is one of the most important processes leading to the emergence of an organ oriented toward sexual reproduction [34]. To achieve the highest fitness, floral traits are largely determined during adaptive evolution by their capability for sexual reproductive success [35]. Flower size, biomass, and number are variable at the inflorescence and individual levels, as they are considered a consequence of the trade-off rule [36]. Therefore, when plants possess both sterile and fertile flowers, a large size is often associated with sterile or infertile flowers, contributing to diverse flower morphologies and structures, such as in *Viburnum* (Adoxaceae), *Hydrangea* (Hydrangeaceae), and Asteraceae. Here, we analyzed the differences between sterile and fertile flowers of *V*. *macrocephalum* f. *keteleeri* and found that the size of sterile petals was more than 20 times that of fertile petals in the LS. Furthermore, the stamens of sterile flowers are short with no anthers, and the pistils contain abnormal embryo sacs, compared with the normal structures of fertile flowers. These vast phenotypic differences between fertile and sterile flowers from the same genetic background are interesting in terms of attempts to understand their underlying molecular mechanisms.

MiRNAs are important regulators of flower development and floral organ differentiation [37]. Although much effort has been devoted to the cloning and identification of key genes involved in floral development and flowering regulation, the role of miRNAs in floral developmental processes such as flower size and flower organ differentiation is poorly understood [38]. In our study, we constructed sRNA populations of VMF and VMS. The two sRNA libraries both have abundant, high-quality data. Known and novel miRNAs were identified, and several of these were verified by sub-cloning techniques. These results suggest that there is a complex and diverse array of sRNAs involved in the development and differentiation of fertile and sterile flowers.

### Known miRNAs and their targets associated with the differentiation and development of flowers

MiRNAs negatively regulate certain genes involved in plant development by directing RNA cleavage or inhibiting translation of target transcripts. Furthermore, molecular and genomic studies in seed plant species have demonstrated that a number of miRNAs and their target genes are involved in the coordinated regulation of flower differentiation and development [39]. For example, miR164 and its targets are thought to be involved in carpel fusion in addition to their roles in sepal and petal boundary development [40]. MiR159 and its targeted GAMYB-related genes are required for normal anther development in *Arabidopsis* and rice [41–43]. Apart from their functions in the formation of normal flower tissue, which may influence fertility, miRNAs also contribute to flower shape and size. In *Arabidopsis*, a loss-of-function miR319a mutant exhibited a reduction in petal width and decreased petal and stamen length, supporting the hypothesis that miRNA319 is critical for controlling the size and shape of floral organs by targeting TCP genes [44]. These findings suggest that miRNAs have diverse biological functions in flower organ development and formation.

In this study, a total of 30 known miRNAs were differentially expressed in VMF and VFS. Among them, 14 miRNAs were significantly upregulated in VMS, whereas the other 16 miRNAs were downregulated in VMS. As for the differentially expressed miRNAs, miR156g, miR156j, miR166e-5p, miR169, and miR398a-3p were detected only in VMF, whereas miR156h, miR162a-5, miR167c-5p, and miR845a were found only in VMS. Additionally, some miRNAs had significantly different expression levels. For example, in miR319 family members, the read counts of miR319a were 4164 and 598, whereas those of miR319c were 1725 and 332 in VMF and VMS, respectively. These results suggest that these differentially expressed miRNAs might be involved in the development of fertile or sterile flowers.

Our transcriptomic analysis showed that many transcription factors exhibit dynamic gene expression changes in *V*. *macrocephalum* f. *keteleeri* flowers [23], and some of these genes are miRNA targets. A total of 190 targets of known miRNAs were predicted and annotated with gene descriptions. For example, miR160 targeted *ARF8*, one of the ARF family members, which is critical for the proper development of stamens and ovules [45]. Additionally, we found some direct development-related miRNAs involved in plant growth and development. For example, miR159 target genes encode F-box proteins, which play several important roles in flower development [46–48]. Furthermore, some miRNA-targeted transcription factors, such as bHLH and NAC, which are involved in biological synthesis and stress resistance, were found in sterile and fertile flowers [49–51]. We predicted that miR159, miR156, and miR394 regulate genes encoding transcription factors of the bHLH family and that miR164 regulates genes encoding transcription factors of the NAC family. The target genes of flower-related miRNAs suggest that miRNAs specific to flower tissues might have broad regulatory functions with regard to transcription factors, hormones, biosynthesis, and stress responses.

miR156 is widely known for repressing the expression of *SPL* genes [52]. *SPL* genes encode plant-specific transcription factors that play important roles in plant phase transition, flower and fruit development, plant architecture, sporogenesis, and response to copper and fungal toxins [53]. In the leaves of *Arabidopsis*, the effects of both miR156-targeted *SPL* genes on organ size are correlated with changes in plastochron length, potentially influencing leaf size [54]. In flowers, besides regulating multiple important and divergent biological processes such as flowering time, *SPL*s also regulate genes mediating cell division, differentiation, and specification in early anther development. Fertile *Arabidopsis* flowers with secure male fertility require the action of multiple miR156/7-targeted *SPL* genes in concert with *SPL8* [55]. Additionally, an SBP-box transcription factor, tasselsheath4 (tsh4), is a target of miR156 and is known to regulate the development of bracts and meristem boundaries in maize [56]. In our study, four members of the miR156 family were obtained in VMS and VMF, including miR156a-5p, miR156g, miR156h, and miR156j. In addition to miR156h, which was expressed only in VMS, the expression levels of the other three were all significantly higher in VMF than in VMS. Based on the findings that sterile flowers produce few anthers, while fertile flowers produce abundant anthers, miR156 might be involved in the regulation of stamen development. The role of miR156 in flower differentiation and development needs to be further determined using molecular genetics studies.

### Targets of the novel miRNA candidates involved in flower development

The characteristic hairpin structure of miRNA precursors was used to predict novel miRNAs [31]. According to the novel miRNA annotation criteria, we obtained unique sRNA sequences with complementary miRNA*s. Among these miRNAs, several novel miRNAs were significantly differentially expressed in fertile and sterile flowers, including miRNAs found only in VMF/VMS. These results suggest that the novel miRNAs might play a regulatory role in the development and differentiation of flowers of *V*. *macrocephalum* f. *keteleeri*.

In terms of the annotation of predicted targets, we found that some novel miRNAs targeted the same transcription factor families as known miRNAs. We predicted that genes encoding bHLH transcription factors are targeted by novel_112, novel_46, novel_96, and novel_62. Additionally, several novel miRNAs (novel_87, 40, 64, 97, 55, and 52) and one known miRNA (miR156) were predicted to target genes encoding cytochrome P450 enzymes, which play important roles in the biosynthesis of flavonoids, including anthocyanins, which contribute to flower color [57]. These results suggest that some novel miRNAs have functions similar to those of known miRNAs in that they target the same transcription factor families or genes. Additionally, we found novel_50 and miR172e_5p targeted the same unigenes. According to a BLAST search with the plant non-coding RNA database website (https://doi.org/10.1093/nar/gku1162), the mature sequence of novel_50 is consistent with miR172c in *Vitis vinifera*; thus, we presume that novel_50 in *V*. *macrocephalum* f. *keteleeri* may be a candidate member of the miR172 family of important regulatory miRNAs in flowers.

Novel miRNAs have their own targets, and these differ from the targets of known miRNAs. We predicted the identification of two transcription factors involved in hormone regulation in *V*. *macrocephalum* f. *keteleeri* flowers. WRKY transcription factors are one of the largest families of transcriptional regulators in plants and form integral parts of signaling webs that modulate many plant processes [58, 59]. Here, in differentially expressed genes, novel_87 and novel_96 were predicted to target c24016_g1 and c38632_g1, respectively, belonging to the WRKY transcription factor family. The transcription factor LATERAL ORGAN BOUNDARIES (LOB) negatively regulates accumulation of the plant hormone brassinosteroid (BR) at organ boundaries [60]. c27700_g1, targeted by novel_87, was annotated as LOB, and c44637_g1, one target of novel_14 in *V*. *macrocephalum* f. keteleeri, was annotated as *GRF3*. Additionally, the novel miRNAs function in stress response and biological processes. Furthermore, we predicted c17130_g1, targeted by novel_96, to encode a WUSCHEL-related homeobox member, which plays crucial roles in plant development by regulating cell division and differentiation [61]. These results suggest that some novel miRNAs, including novel_87, novel_96, and novel_14, play important roles in hormone regulation, disease resistance, and biological processes during the differentiation and development of fertile and sterile flowers in *V*. *macrocephalum* f. *keteleeri*.

## Conclusion

The fertile and sterile flowers of *V*. *macrocephalum* f. *keteleeri* show a distinct disparity in morphology and fertility; these differences are the results of differences in petal cell size, number, and the differentiation of stamens and carpels. Two sRNA libraries were constructed, and miRNAs differentially expressed between fertile and sterile flowers were identified. Some miRNA precursors were validated by sub-cloning, and the dynamic expression levels of miRNAs, such as miR160, miR156, miR164, novel_87, novel_14, and novel_96, and their target genes were determined by qRT-PCR. Our work showed that miRNAs potentially play roles in differentiation and development of fertile and sterile flowers.

## Additional files

Additional file 1: All the primers used in this study, including 29 pairs of primers for precursor sequences,10 pairs of primers for quantitative real-time PCR of precursor sequences, and 6 pairs of primers for quantitative real-time PCR of target genes. (XLSX 16 kb)

Additional file 2: Summary of clean 18–30 nt sRNAs. (XLSX 10 kb)

Additional file 3: Summary of mapped sRNAs. (XLSX 10 kb)

Additional file 4: Non-coding RNAs among the sRNAs. (XLSX 10 kb)

Additional file 5: Fold-back structures for known miRNA from *Viburnum macrocephalum* f. *keteleeri*. Precursor sequences for known miRNAs were shown in black letters with miRNA sequences highlighted in red. (PDF 1539 kb)

Additional file 6: Nucleotide bias analysis of known miRNAs in VMF and VMS. (PDF 231 kb)

Additional file 7: Known miRNAs in *Viburnum macrocephalum* f. *keteleeri* flowers, including family members, mature sequence, length, and expression levels in fertile (VMF) and sterile (VMS) flowers. (XLS 37 kb)

Additional file 8: Fold-back structures for novel miRNA from *Viburnum macrocephalum* f. *keteleeri*. Precursor sequences for novel miRNAs were shown in black letters with miRNA sequences highlighted in red. Precursor secondary structures and MEF value were produced using the MFOLD (http://unafold.rna.albany.edu/?q=mfold/) software. Precursor sequences for novel miRNA*s were shown in black letters with miRNA sequences highlighted in green. (PDF 2121 kb)

Additional file 9: Novel miRNA*s in *Viburnum macrocephalum* f. *keteleeri* flowers, including expression levels in VMF and VMS. (XLS 22 kb)

Additional file 10: Nucleotide bias analysis of novel miRNAs in VMF and VMS. (PDF 237 kb)

Additional file 11: Novel miRNAs in *Viburnum macrocephalum* f. *keteleeri* flowers, including expression levels in VMF and VMS. (XLSX 11 kb)

Additional file 12: Known miRNA and predicted targets. (XLSX 19 kb)

Additional file 13: Novel miRNA and predicted targets. (XLSX 29 kb)

Additional file 14: Known miRNA and predicted targets with annotation. (XLSX 24 kb)

Additional file 15: Novel miRNA and predicted targets with annotation. (XLSX 39 kb)

Additional file 16: Normalized expression of miRNAs. Log2 Fold_change and *p*-value are obtained for searching differentially expressed miRNAs. (XLSX 13 kb)

Additional file 17: Up-regulated miRNAs in VMS and related mRNA. (XLSX 10 kb)

Additional file 18: Down-regulated miRNAs in VMS and related mRNA. (XLSX 10 kb)

Additional file 19: Differentially expressed genes with gene description. (XLSX 606 kb)

Additional file 20: miRNAs have opposite tendency between qRT-PCR and sRNA sequencing. (PDF 420 kb)

Additional file 21: Expression changes of miRNAs at different developmental stages between VMF and VMS. (PDF 2509 kb)

Additional file 22: Expression changes between miRNAs and targets at different developmental stages. (PDF 1595 kb)

## Abbreviations

*ARF*: *Auxin response factor*
ES: Early stage
*GRFS*: *Growth-regulating factors*
LS: Later stage
miRNA: micro RNA
MS: Middle stage
SBP: Squamosa promoter binding protein
*SPL*: *Squamosa promoter binding protein-like*
sRNA: small RNA
*TCP*: *Teosinte Branched/Cycloidea/PCF*
VMF: Fertile flowers in *Viburnum macrocephalum* f. *keteleeri*
VMS: Sterile flowers in *Viburnum macrocephalum* f. *keteleeri*
