# Supplementary material for: miRNAs involved in the development and differentiation of fertile and sterile flowers in Viburnum macrocephalum f. keteleeri
Source: BMC Genomics. 2017 Oct 13;18:783. doi: 10.1186/s12864-017-4180-x (PMC5640959; doi:10.1186/s12864-017-4180-x)

Additional file 5. Fold-back structures for known miRNA from *Viburnum macrocephalum* f. *keteleeri*. Precursor sequences for known miRNAs were shown in black letters with miRNA sequences highlighted in red.

miR156a-5p
CAAGAGAAACGCAAAGAAACUGACAGAAGAGAGUGAGCACACAAAGGCAAUUUGC
AUAUCAUUGCACUUGCUUCUCUUGCGUGCUCACUGCUCUUUCUGUCAGAUUCCGG
UGCUGAUCUCUUU

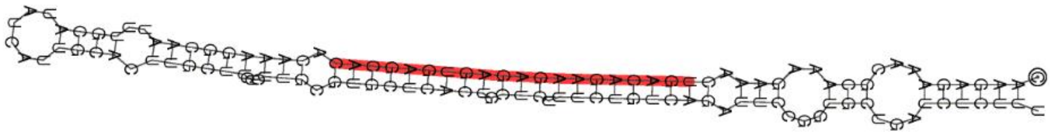

miR156g
AUAACGAAGGCGACAGAAGAGAGUGAGCACACAUGGCUCUUUUUCUAGCAUGCUC
AUGCUCGAAAGCUCUGCGUGCUUACUCUCUUCUUGUCUCCUGCUCUCU

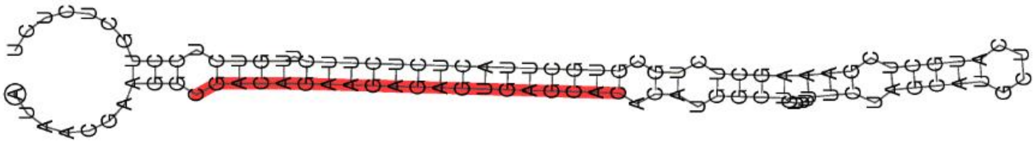

miR156h
AUGAAAAAUGUUGACAGAAGAAAGAGAGCACAACCUGGGAUUAGCAAAAAGAUAG
UUUUGCCCUUGUCGGGAGUGUGCUCUCUUUCCUUCUGCCACCAUCAUUGCG

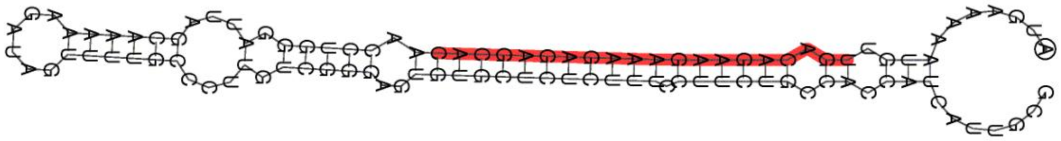

miR156j
UUUGACAGAAGAGAGAGAGCACAGUUUGAGUCGCCAAUUCCCUUGUAGCUCUCUA
GUUUAGUGUCCAU

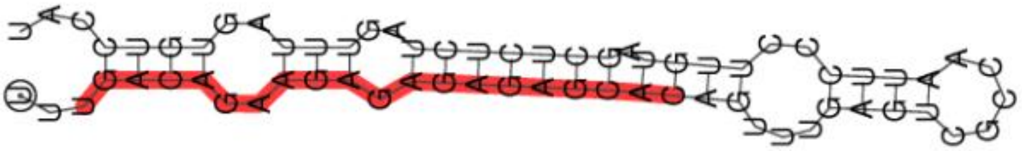

miR157a-5p

GUGUUGACAGAAGAUAGAGAGCACAGAUGAUGAGAUACAAUUCGGAGCAUGUUCU
UUGCAUCUUACUCCUUUGUGCUCUCUAGCCUUCUGUCAUCACC

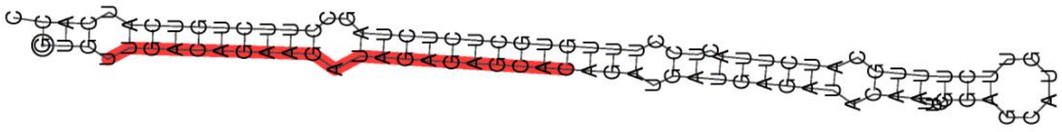

miR157d

GUGGAGGGUGAUAGUGUGGUUGCUGACAGAAGAUAGAGAGCACUAAGGAUGCUAU
GCAAAACAGACACAGAUAUGUGUUUCUAAUUGUAUUUCAUACUUUAACCUCAAAG
UUGAUAUAAAAAAAGAAAGAAAGAUAGAAGAGCUAGAAGACUAUCUGCAUCUCUA
UUCCUAUGUGCUCUCUAUGCUUCUGUCAUCACCUUUCUUUCUCUAUUUCUCUCUAC

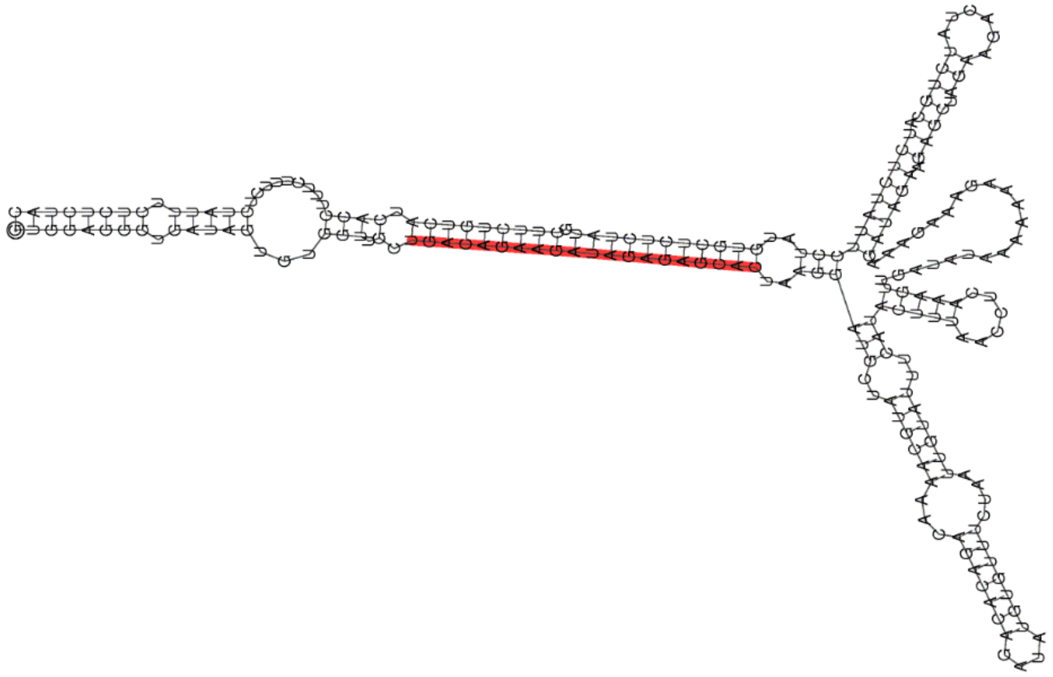

miR159a

GUAGAGCUCCUUAAAGUUCAAACAUGAGUUGAGCAGGGUAAAGAAAAGCUGCUAA
GCUAUGGAUCCCAUAAGCCCUAAUCCUUGUAAAGUAAAAAAGGAUUUGGUUAUAU
GGAUUGCAUAUCUCAGGAGCUUUAACUUGCCCUUUAAUGGCUUUUACUCUUCUUU
GGAUUGAAGGGAGCUCUAC

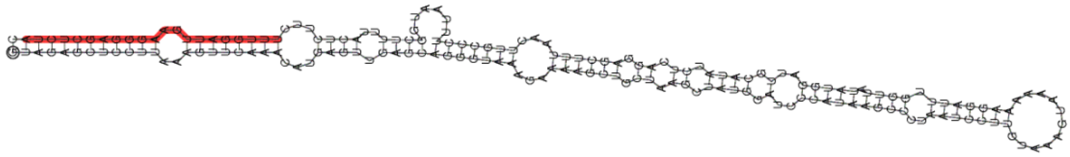

miR159b-3p

GGAAGAGCUCCUUGAAGUUCAAUGGAGGGUUUAGCAGGGUGAAGUAAAGCUGCUA
AGCUAUGGAUCCCAUAAGCCUUAUCAAAUUCAAUAUAAUUGAUGAUAAGGUUUUU
UUUAUGGAUGCCAUAUCUCAGGAGCUUUCACUUACCCCUUUAAUGGCUUCACUCU
UCUUUGGAUUGAAGGGAGCUCUUCAUCUCUC

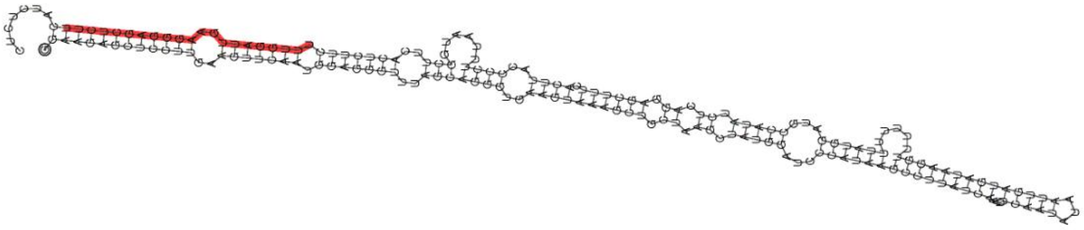

miR159c

GUGUAACAGAAGGAGCUCCCUUCCUCCAAAACGAAGAGGACAAGAUUUGAGGAAC
UAAAAUGCAGAAUCUAAGAGUUCAUGUCUUCCUCAUAGAGAGUGCGCGGUGUUAA
AAGCUUGAAGAAAGCACACUUUAAGGGGAUUGCACGACCUCUUAGAUUCUCCCUC
UUUCUCUACAUAUCAUUCUCUUCUCUUCGUUUGGAUUGAAGGGAGCUCCUUUUCU
UCUUC

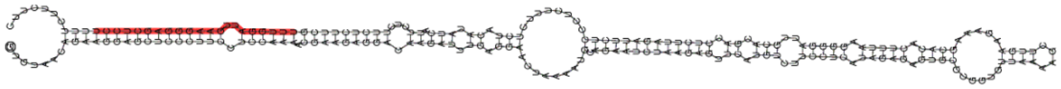

miR160a-5p

GUAUGCCUGGCUCCCUGUAUGCCAUAUGCUGAGCCCAUCGAGUAUCGAUGACCUCC
GUGGAUGGCGUAUGAGGAGCCAUGCAUAU

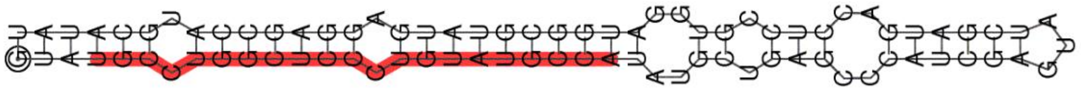

miR162a-3p

UAGUUGGAAGAAGAGUGAGAGUCGCUGGAGGCAGCGGUUCAUCGAUCUCUUCCUG
UGAACACAUUAAAAAUGUAAAAGCAUGAAUAGAUCGAUAAACCUCUGCAUCCAGC
GUUUGCCUCUUGUAUCUUUCUUAUUGACUU

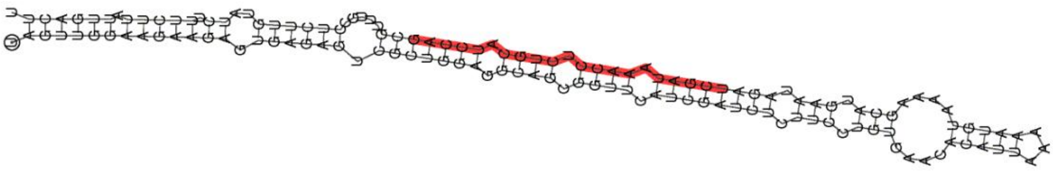

miR162a-5p

UAGUUGGAAGAAGAGUGAGAGUCGCUGGAGGCAGCGGUUCAUCGAUCUCUUCCUG
UGAACACAUUAAAAAUGUAAAAGCAUGAAUAGAUCGAUAAACCUCUGCAUCCAGC
GUUUGCCUCUUGUAUCUUUCUUAUUGACUU

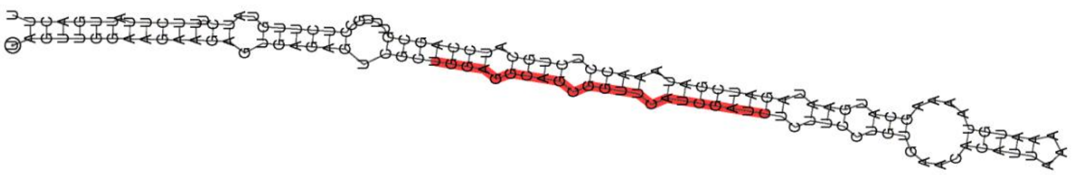

miR164a
GGGUGAGAAUCUCCAUGUUGGAGAAGCAGGGCACGUGCAAACCAACAAACACGAA
AUCCGUCUCAUUUGCUUAUUUGCACGUACUUAACUUCUCCAACAUGAGCUCUUCAC
CC

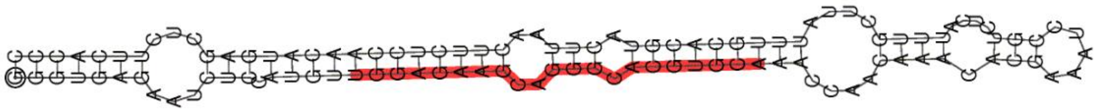

miR164c-5p
UAACACUUGAUGGAGAAGCAGGGCACGUGCGAACACAAAUGAAAUCGAUCGGUAC
UUGUUGAUCAUAUUUUCGCACGUGUUCUACUACUCCAACACGUGUCU

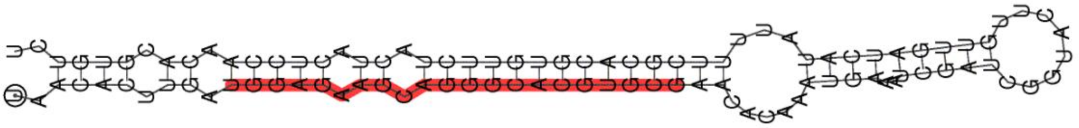

miR165a-3p
GUUGAGGGGAAUGUUGUCUGGAUCGAGGAUAUUAUAGAUAUAUACAUGUGUAUG
UUAAUGAUUCAAGUGAUCAUAGAGAGUAUCCUCGGACCAGGCUUCAUCCCCCCA
AC

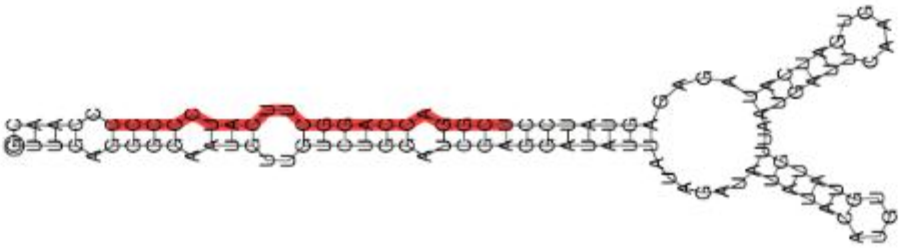

miR166a-3p
AGGGGCUUUCUCUUUUGAGGGGACUGUUGUCUGGCUCGAGGACUCUGGCUCGCUC
UAUUCAUGUUGGAUCUCUUUCGAUCUAACAAUCGAAUUGAACCUUCAGAUUUCAG
AUUUGAUUAGGGUUUUAGCGUCUUCGGACCAGGCUUCAUUCCCCCCAAUUGUUGC
UCCCU

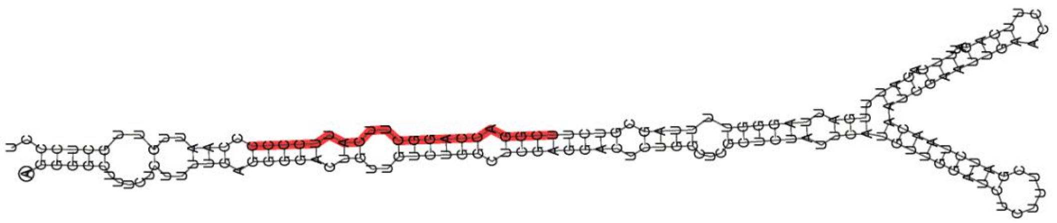

miR166e-5p

UUGAGGGGAAUGUUGUCUGGCACGAGGCCCUUAACUUAGAUCUAUAUUUGAUUAU
AUAUAUAUGUCUCUUCUUUAUUCAUUAGUCUAUACAUGAAUGAUCAUUUUACGGU
UAAUGACGUCGGACCAGGCUUCAUUCCCCUCAA

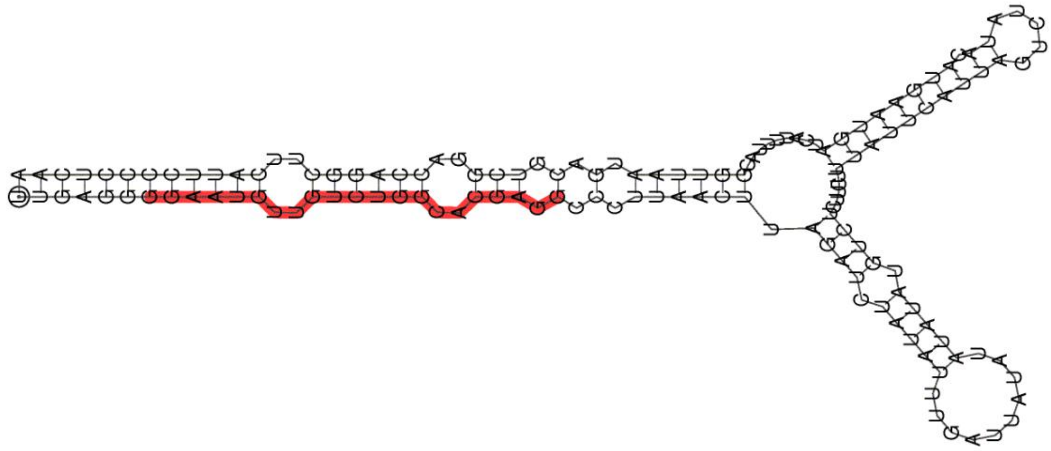

miR167a-5p

UGGUGCACCGGCAUCUGAUGAAGCUGCCAGCAUGAUCUAAUUAGCUUUCUUUAUC
CUUUGUUGUGUUUCAUGACGAUGGUUAAGAGAUCAGUCUCGAUUAGAUCAUGUUC
GCAGUUUCACCCGUUGACUGUCGCACCC

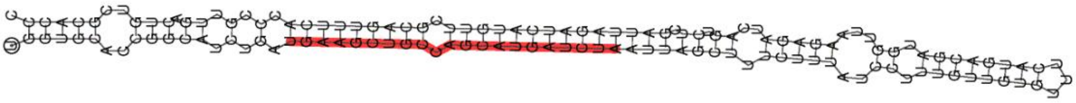

miR167c-5p

CCAGUAGCAGUUAAGCUGCCAGCAUGAUCUUGUCUUCCUCUCUUAGGUUUCAUAU
AUAGUUAAUAAAUAUUUUAUAUAUUUCUUGUUCUUACAAGAUUAUAUGAUCAUAG
CUUAGAGAGAGAGAGAGACUAGGUCAUGCUGGUAGUUUCACCUGCUAAUG

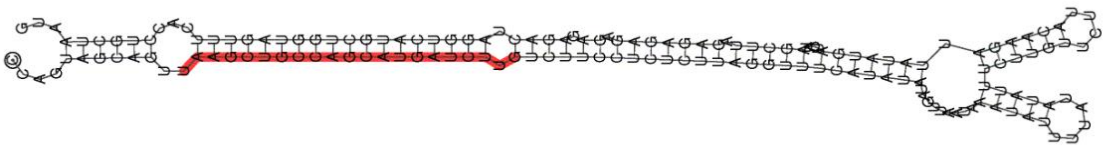

miR167d

UGUUGGUUUUUAGAAGCUGAAGCUGCCAGCAUGAUCUGGUAAUCGCUACAUACGA
CAUACACACAUCACUAAACUUCUUUAUAAUUUAUGCACACACAUACAGCUCUUAA
UGGCCACAACUCAAAGUUAUAAUUAGUGCAUGAUCUCUAGUUAUUUGACUGCUUU
UAAUAUAUGUUUAUGGAUUCACGCAUGUGUGUGUAUGUACAUAAUUUACAUGCAU
GCACUUUGUGUAUGGUACACAUCAAUUUGAACCCGUUCAAAAUUCUGUUUUUAUU
AGUAUAUAUAUAGAUGUAUGUGGUGUGUGUGUCAGUGUGUGUGUGUGUUUAUAG
AUAGUAGUACUAGGUCAUCCUGCAGCUUCAGUCACUAAAUCACCAACA

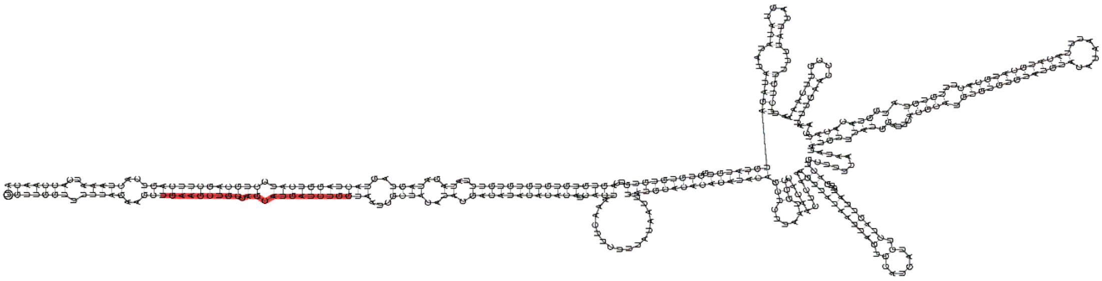

miR168a-3p

CACCAUCGGGCUCGGAUUCGCUUGGUGCAGGUCGGGAACCAAUUCGGCUGACACA
GCCUCGUGACUUUUAAACCUUUAUUGGUUUGUGAGCAGGGAUUGGAUCCCGCCUU
GCAUCAACUGAAUCGGAUCCUCGAGGUG

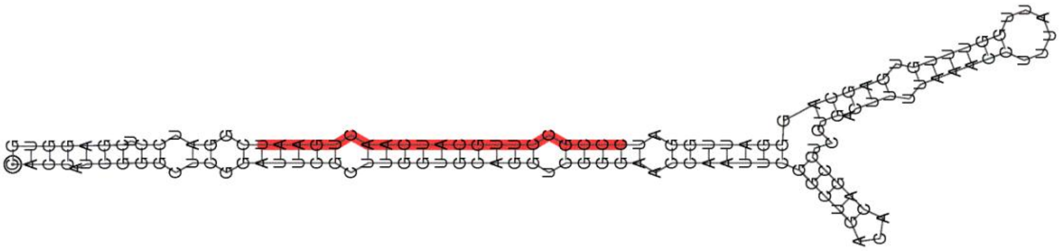

miR168a-5p

CACCAUCGGGCUCGGAUUCGCUUGGUGCAGGUCGGGAACCAAUUCGGCUGACACA
GCCUCGUGACUUUUAAACCUUUAUUGGUUUGUGAGCAGGGAUUGGAUCCCGCCUU
GCAUCAACUGAAUCGGAUCCUCGAGGUG

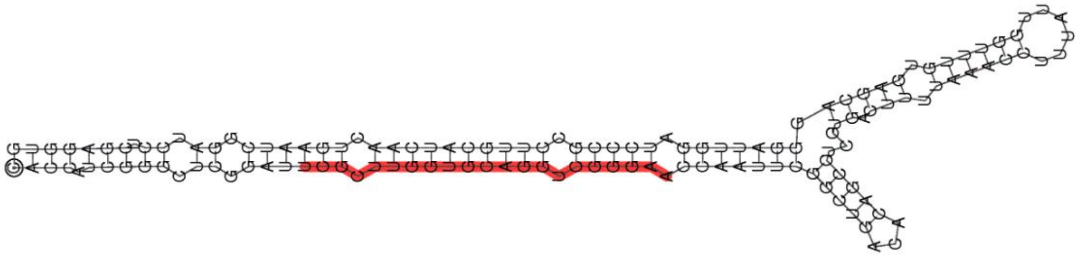

miR169a-5p

GUGACGAAAGUAGUGUGCAGCCAAGGAUGACUUGCCGAUUUAAAUGAUCUUUCUU
UAUACUCUAUUAAGACAAUUUAGUUUCAAACUUUUUUUUUUUUUUUUUUUUGAAG
GAUUCAGGAAGAAAUUAGGAUAUAUUAUUCCGUAUAAAAUACAAGAUAUAUAAAA

CCAAAAAGAAAAAGUAACAUGAUCGGCAAGUUGUCCUUGGCUACACGUUACUUUGUGUCGC

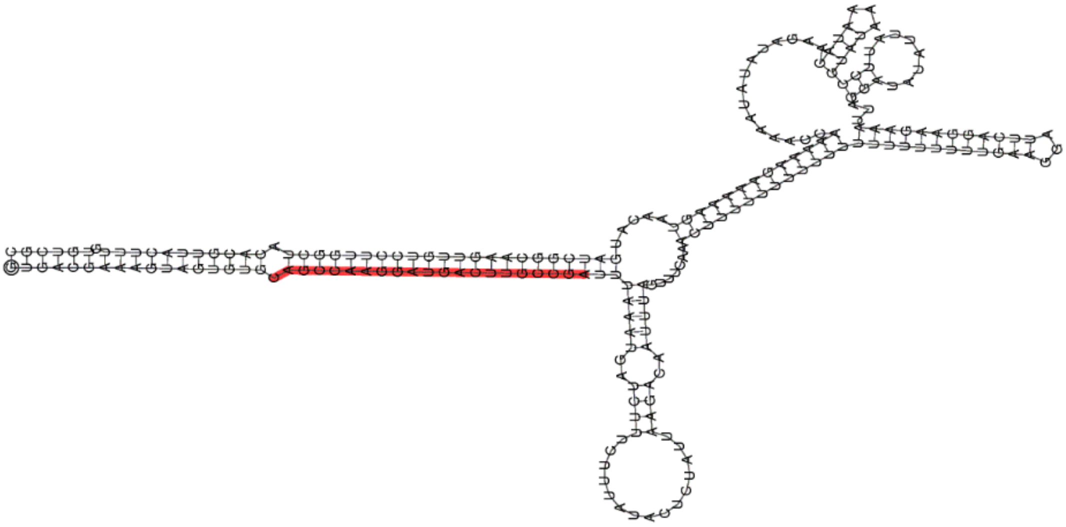

miR169b-5p

CCCAACGGAGUAGAAUUGCAUGAAGUGGAGUAGAGUAUAAUGCAGCCAAGGAUGACUUGCCGGAACGUUGUUAACCAUGCAUAUGAAUAAUGUGAUGAUUAAUUAUGUGAUGAACAUAUUUCUGGCAAGUUGUCCUUCGGCUACAUUUUGCUCUCUUCUUCUCAUGCAAACUUUCCUUGGG

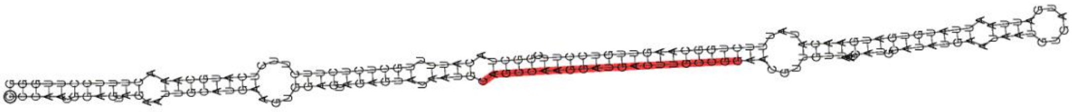

miR169h

UCAUAUAAGAGAAAAUGGUGACAUGAAGAAUGAGAACUUGUGUGGUAGCCAAGGAUGACUUGCCUGCGUUUUAGACCAUAUAUAUCAAAGACUCACUCGAUCGAUAGUCUUAGAGUUGGUUGGUCGUCAGGCAGUCUCCUUGGCUAUUCAAACAAUUCUCAUUCUCUUCAUUCACAUUUCUCUUUUUUGG

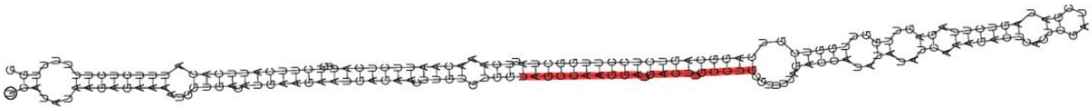

miR171a-3p

AUGAGAGAGUCCCUUUGAUAUUGGCCUGGUUCACUCAGAUCUUACCUGACCACACACGUAGAUAUACAUUAUUCUCUCUAGAUUAUCUGAUUGAGCCGCGCCAAUAUCUCAGUACUCUCUCGU

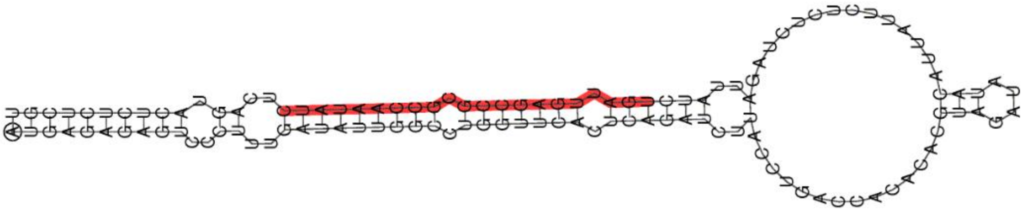

miR171b-3p

UGCAAGGUAACGCGAGAUAUUAGUGCGGUUCAAUCAAAUAGUCGUCCUCUUAACU
CAUGGAGAACGGUGUUGUUCGAUUGAGCCGUGCCAAUAUCACGCGGUAAACCAAA
AAUGGCA

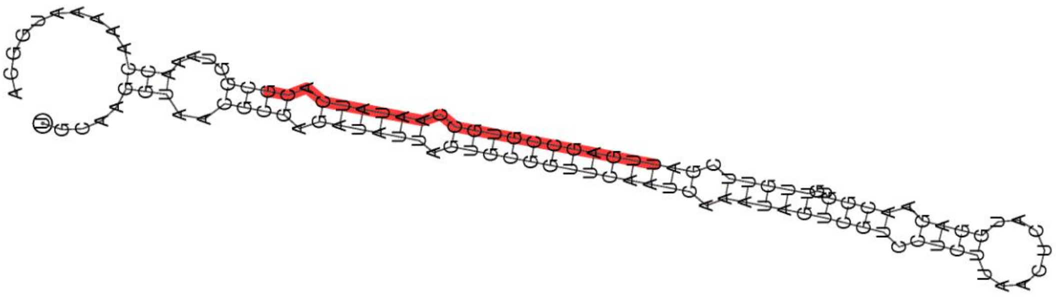

miR172a

UGCUGUGGCAUCAUCAAGAUUCACAUCUGUUGAUGGACGGUGGUGAUUCACUCUC
CACAAAGUUCUCUAUGAAAAUGAGAAUCUUGAUGAUGCUGCAUCGGC

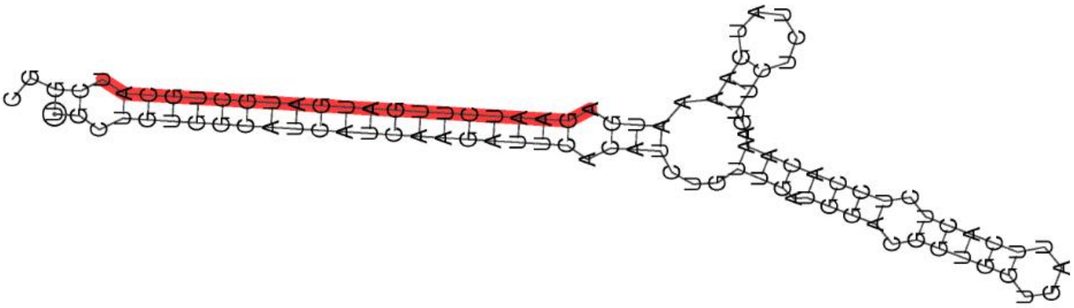

miR172c

AGCUACUGUUCGCUGUUGGAGCAUCAUCAAGAUUCACAAAUCAUCAAGUAUUCGU
GUAAAUAAACCCAUUUAUGAUUAGAUUUUUGAUGUAUGUAUGAGAAUCUUGAUGA
UGCUGCAGCUGCAAUCAGUGGCU

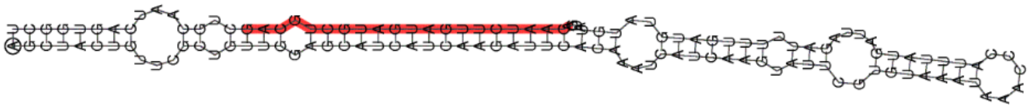

miR172e

GUAGUCGCAGAUGCAGCACCAUUAAGAUUCACAAGAGAUGUGGUUCCCUUUGCUU
UCGCCUCUCGAUCCGCAGAAAAGGGUUCCUUAUCGAGUGGGAAUCUUGAUGAUGC
UGCAUCAGCAAAUAC

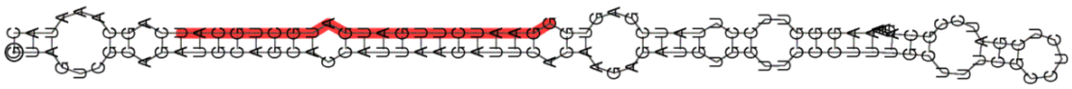

miR319a

AGAGAGAGCUUCCUUGAGUCCAUUCACAGGUCGUGAUAUGAUUCAAUUAGCUUCC
GACUCAUUCAUCCAAAUACCGAGUCGCCAAAAUUCAAACUAGACUCGUUAAAUGA
AUGAAUGAUGCGGUAGACAAAUUGGAUCAUUGAUUCUCUUUGAUUGGACUGAAGG
GAGCUCCCUCU

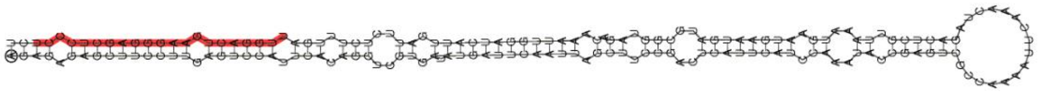

miR319c

UAGAUAUAGAAGGAGAUUCUUUCAGUCCAGUCAUGGAUAGAAAAAGAAGAGGGUA
GAAAUAUCUGCCGACUCAUCCAUCCAAACACUCGUGGUAGAGAAACGAUAAAUUU
AAACCGCAGUGACUGUGUGAAUGAUGCGGGAGAUAUUUUUGAUCCUUCUUUAUCU
GUGUUUGGACUGAAGGGAGCUCCUUCUUUUUCUA

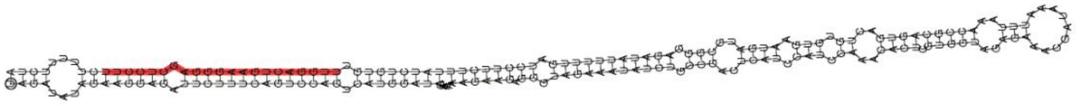

miR390a-3p

GUAGAGAAGAAUCUGUAAAGCUCAGGAGGGAUAGCGCCAUGAUGAUCACAUUCGU
UAUCUAUUUUUUGGCGCUAUCCAUCCUGAGUUUCAUUGGCUCUUCUUACUAC

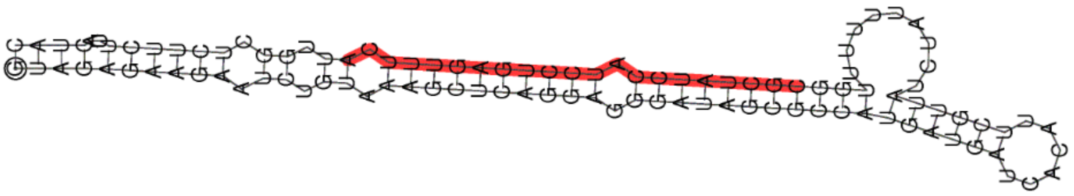

miR390a-5p

GUAGAGAAGAAUCUGUAAAGCUCAGGAGGGAUAGCGCCAUGAUGAUCACAUUCGU
UAUCUAUUUUUUGGCGCUAUCCAUCCUGAGUUUCAUUGGCUCUUCUUACUAC

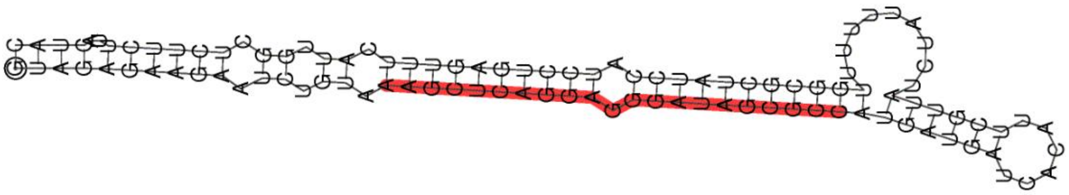

miR390b-3p

GAGAAAUAGCUAUAAAGCUCAGGAGGGAUAGCGCCAUGGCUCACCAGUGCUGUAU
GUUUUGUAUAUGCGUACAUGUAUAUCUGUUGGCGCUAUCCAUCCUGAGUUCCAUA
GCUUCUUCUU

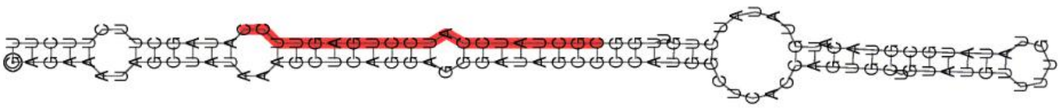

miR393a-5p
AGAGGAAGGAUCCAAAGGGAUCGCAUUGAUCCUAAUUAAGGUGAAUUCUCCCCAU
AUUUUCUUUAUAAUUGGCAAAUAAAUCACAAAAAUUUGCUUGGUUUUGGAUCAUG
CUAUCUCUUUGGAUUCAUCCUUC

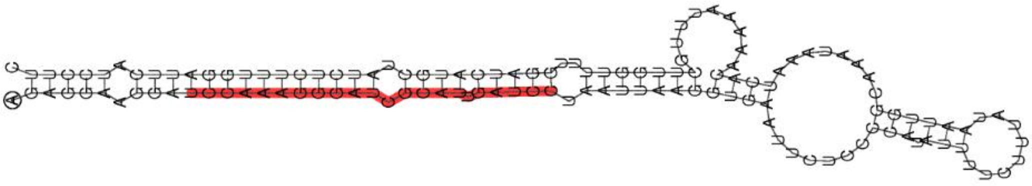

miR394a
CUUACAGUCAUCUUUGGCAUUCUGUCCACCUCCUUCUAUACAUAUAUGCAUGUGU
AUAUAUAUAUGCGUUUCGUGUGAAAGAAGGAGGUGGGUAUACUGCCAAUAGAGAU
CUGUUAG

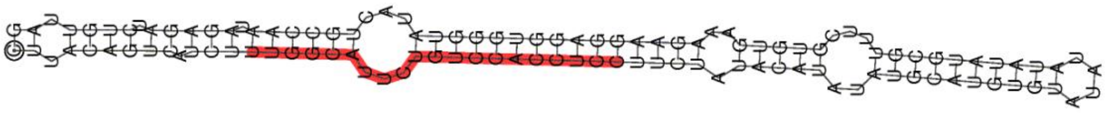

miR396a-3p
CUCUGUAUUCUUCCACAGCUUUCUUGAACUGCAAAACUUCUUCAGAUUUUUUUUU
UUUUCUUUUGAUAUCUCUUACGCAUAAAAUAGUGAUUUUCUUCAUAUCUCUGCUC
GAUUGAUUUGCGGUUCAAUAAAGCUGUGGGAAGAUACAGAC

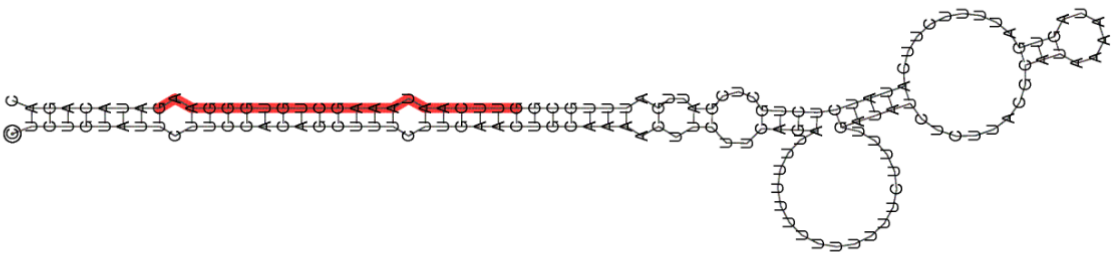

miR396a-5p
CUCUGUAUUCUUCCACAGCUUUCUUGAACUGCAAAACUUCUUCAGAUUUUUUUUU
UUUUCUUUUGAUAUCUCUUACGCAUAAAAUAGUGAUUUUCUUCAUAUCUCUGCUC
GAUUGAUUUGCGGUUCAAUAAAGCUGUGGGAAGAUACAGAC

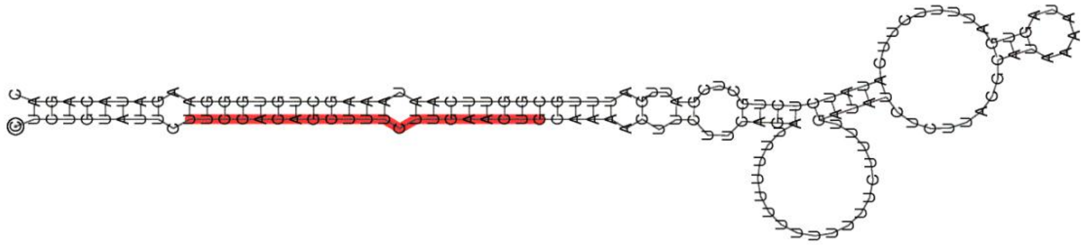

miR396b-3p
GGUCAUACUUUUCCACAGCUUUCUUGAACUUUCUUUUUCAUUUCCAUUGUUUUUU
UCUUAAACAAAAGUAAGAAGAAAAAAAACUUUAAGAUUAAGCAUUUUGGAAGCUC
AAGAAAGCUGUGGGAAAACAUGACA

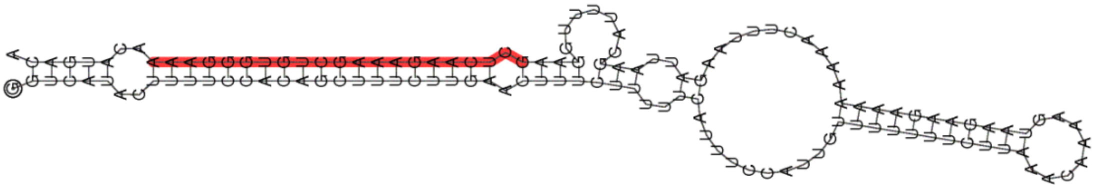

miR396b-5p
GGUCAUACUUUUCCACAGCUUUCUUGAACUUUCUUUUUCAUUUCCAUUGUUUUUU
UCUUAAACAAAAGUAAGAAGAAAAAAAACUUUAAGAUUAAGCAUUUUGGAAGCUC
AAGAAAGCUGUGGGAAAACAUGACA

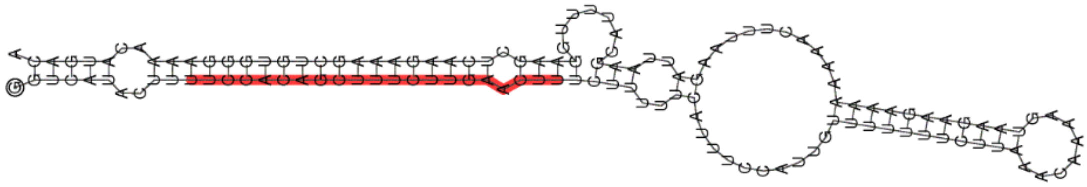

miR397a
UGAAUGAACAUCAUUGAGUGCAGCGUUGAUGUAAUUUCGUUUUGUUUUUCAUUGU
UGAAUGGAUUAAAAGAAUUUAUACCAGCGUUGCGCUCAAUUAUGUUUUUCUA

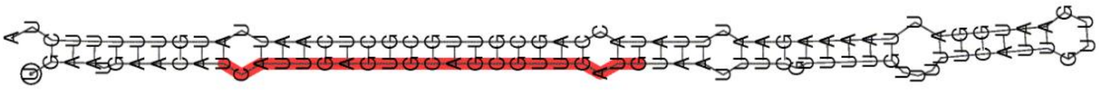

miR398a-3p
UGAAAUUUCAAAGGAGUGGCAUGUGAACACAUAUCCUAUGGUUUCUUCAAAUUUC
CAUUGAAACCAUUGAGUUUUGUGUUCUCAGGUCACCCCUUUGAAUCUCCC

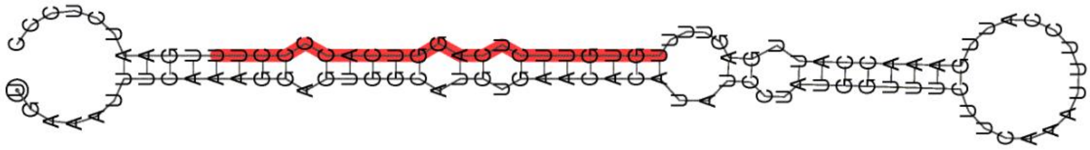

miR398b-3p

UGGAUCUCGACAGGGUUGAUAUGAGAACACACGAGUAAUCAACGGCUGUAAUGAC
GCUACGUCAUUGUUACAGCUCUCGUUUUCAUGUGUUCUCAGGUCACCCCUGCUGA
GCUCUU

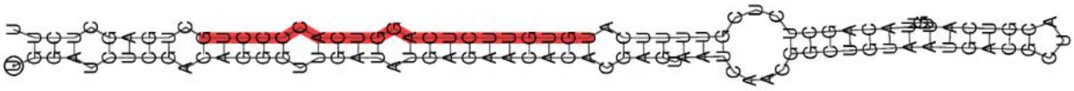

miR403-3p

UUGUCAUUAGAAGAGUCGUAUUACAUGUUUUGUGCUUGAAUCUAAUUCAACAGGC
UUUAUGUAAGAGAUUCUUUAACAAUUCCUAUAAUCUUUGUUGUUGGAUUAGAUUC
ACGCACAAACUCGUAAUCUGUCUUU

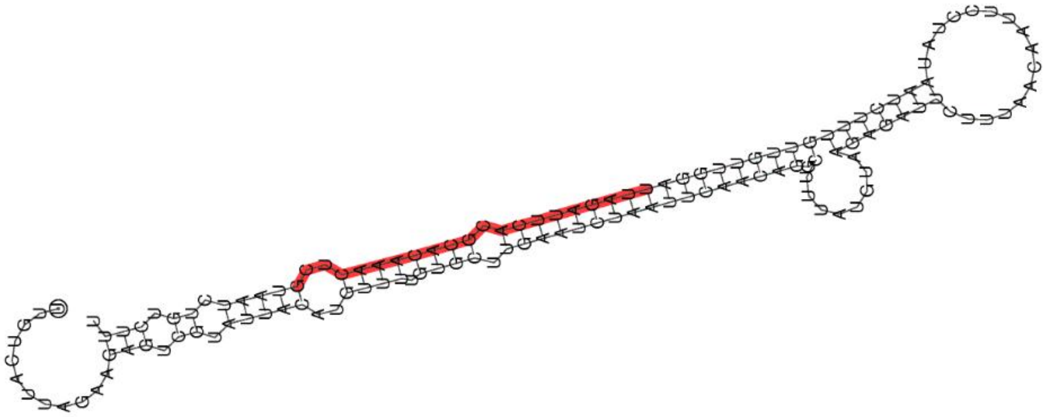

miR408-3p

AAGGUUAGAUUGGUAUUGCAAUGAAAGAAGACAAAGCGGUAAUGAGAGAGAGACA
GGGAACAAGCAGAGCAUGGAUUGAGUUUACUAAAACAUUAAACGACUCUGUUUUG
UCUCUACCCAUGCACUGCCUCUUCCCUGGCUCCCUCUUUUUUUCUCUAUAUUUCUC
UCUCUCCUUUCAUUUCACAGCUUUCAAUGGAAUUUUAUUGCUACUGCUAACG

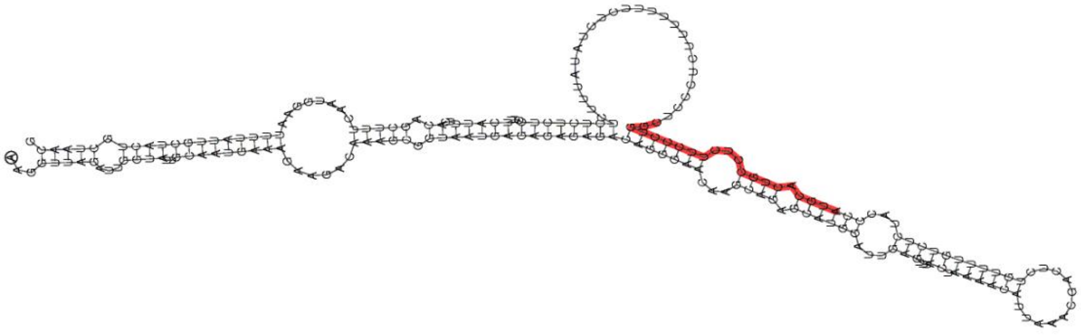

miR845a

AGAUAAGGGAUUGCACGAUUUCUCAAUAGUUCCGUCGCUGCGUUUCCAUCACGUC
GAUUGCUGUCAGAGCCACGCCUAUGAAGAAUUUGUGCGGCUCUGAUACCAAUUGA
UGCUAUGGAAACAUCGAUAUUGCCUGAUUCCGUGUUUCCUUUUACCUUGUUGU

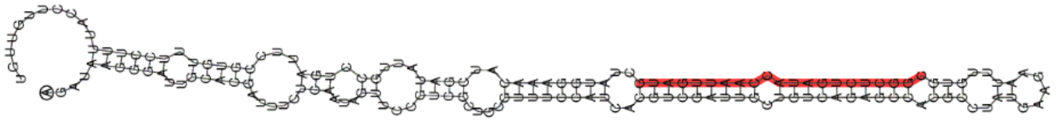

miR2111a-5p
CGAUGAUGAGUAUUGGUGAGGACCGGGUAAUCUGCAUCCUGAGGUUUAAAGCUUA
AUUUACGCAGGAAAUUUGUAUACGCAUAUACGUAUGUGUAUUAGUAUACCUUUUA
GUCCUCGGGAUGCGGAUUACCUCGUUCUUACUUACAAUACACGUACUCG

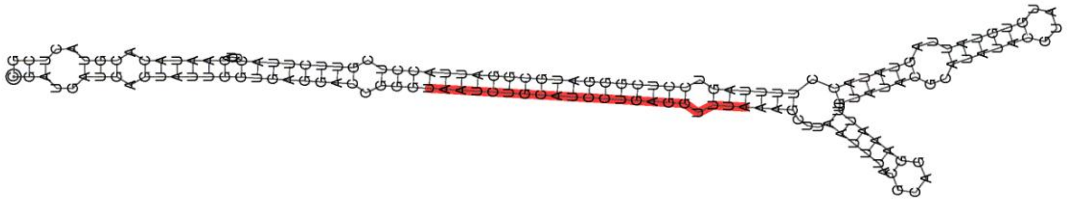

miR8175
CUUAAGGCAAUUUGACCUAUAUCAAAUUUGGCACCGUUGCCGGGGAUCGACCCGG
GUCACCCGCGUGACAGGCGGGAAUACUUACCACUUAGUACAACGACCCAAUAUAG
UGGUAAGUAUUCUCGCAUGUCACUCGGGUUCGAUCCCCGGCAACGGCGCCAAAUU
UGAUAUAGCUCAAAUCGCCUUAAG

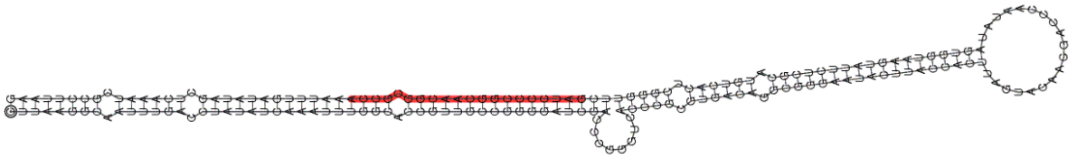

Supplement: Supplementary file 5 — Fold-back structures for known miRNA from Viburnum macrocephalum f. keteleeri. Precursor sequences for known miRNAs were shown in black letters with miRNA sequences highlighted in red. (PDF 1539 kb) [file 12864_2017_4180_MOESM5_ESM.pdf]
