# Supplementary figures and images for: miRNAs involved in the development and differentiation of fertile and sterile flowers in Viburnum macrocephalum f. keteleeri
Source: BMC Genomics. 2017 Oct 13;18:783. doi: 10.1186/s12864-017-4180-x (PMC5640959; doi:10.1186/s12864-017-4180-x)

**Additional file 6.** Nucleotide bias analysis of known miRNAs in VMF and VMS.

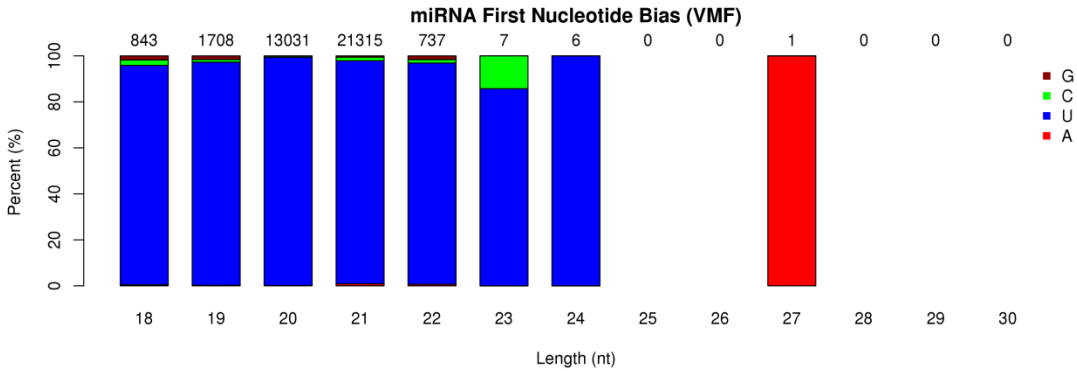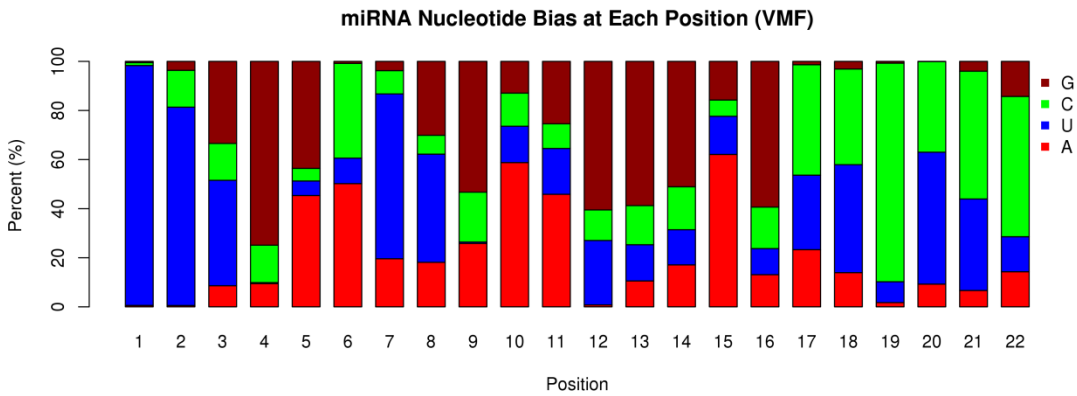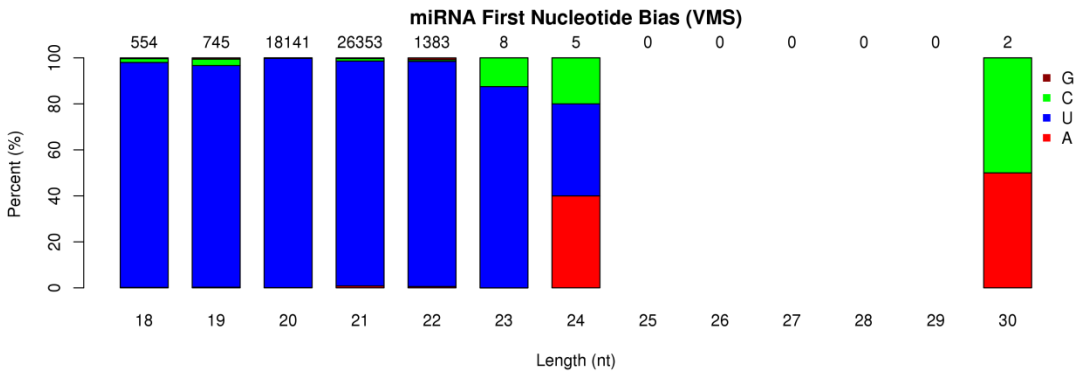

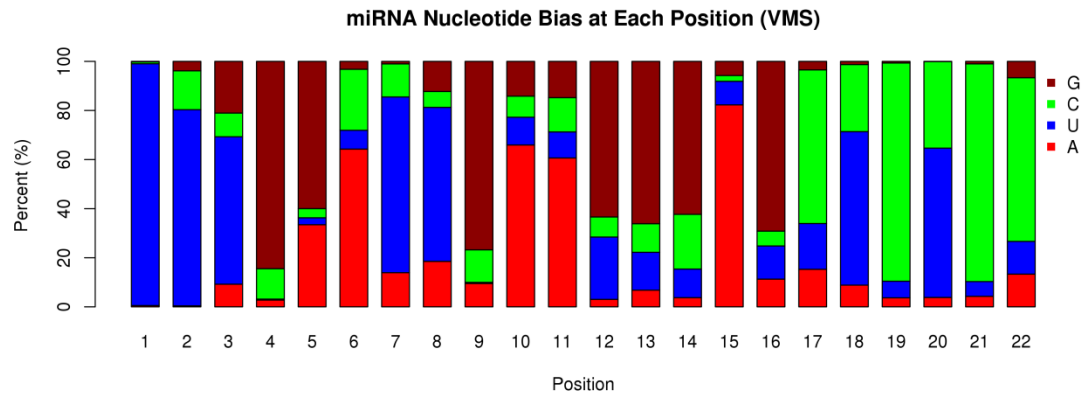

Supplement: Supplementary file 6 — Nucleotide bias analysis of known miRNAs in VMF and VMS. (PDF 231 kb) [file 12864_2017_4180_MOESM6_ESM.pdf]

**Additional file 10.** Nucleotide bias analysis of novel miRNAs in VMF and VMS.

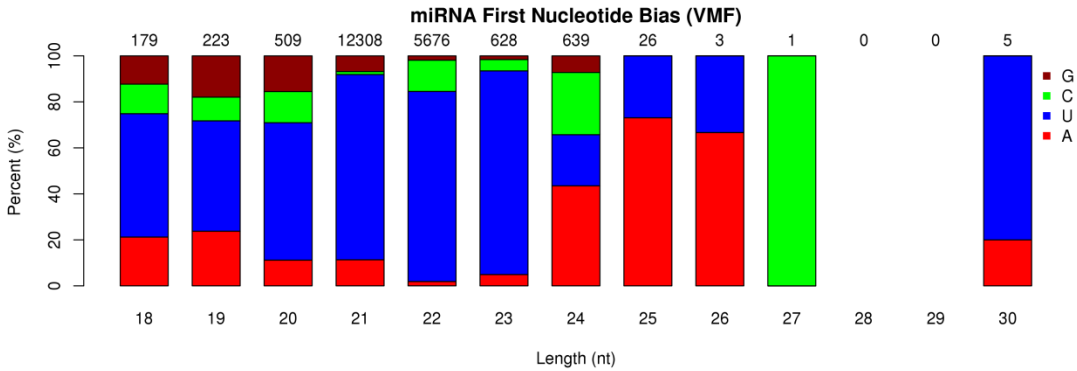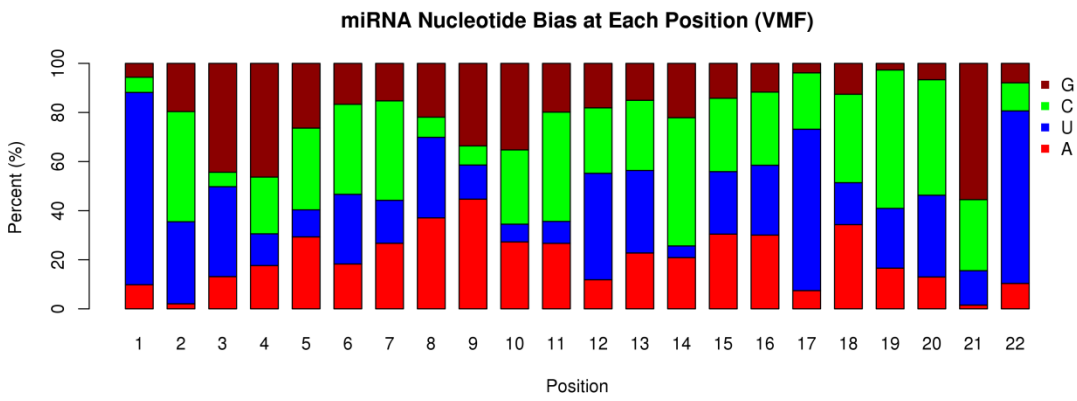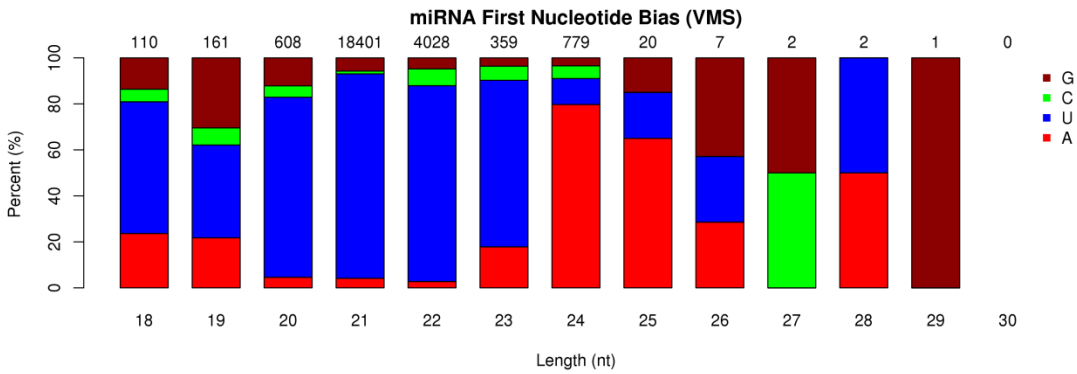

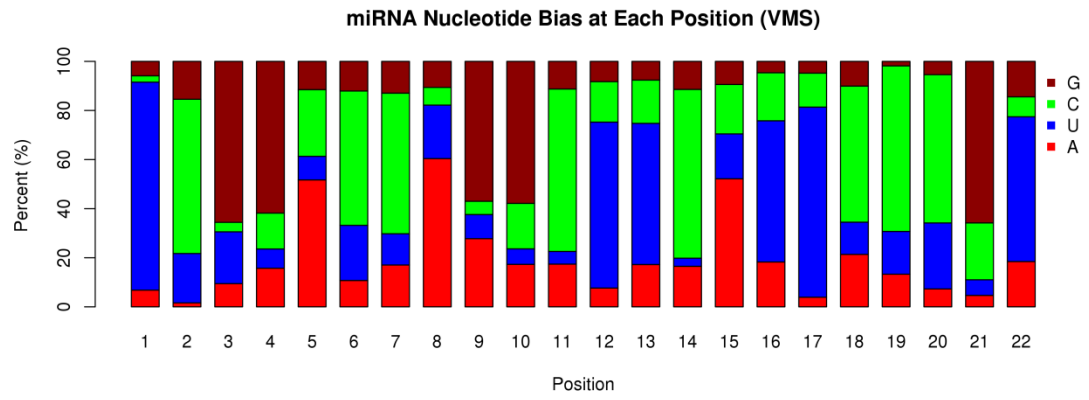

Supplement: Supplementary file 10 — Nucleotide bias analysis of novel miRNAs in VMF and VMS. (PDF 237 kb) [file 12864_2017_4180_MOESM10_ESM.pdf]

**Additional file 20.** miRNAs have opposite tendency between qRT-PCR and sRNA sequencing.

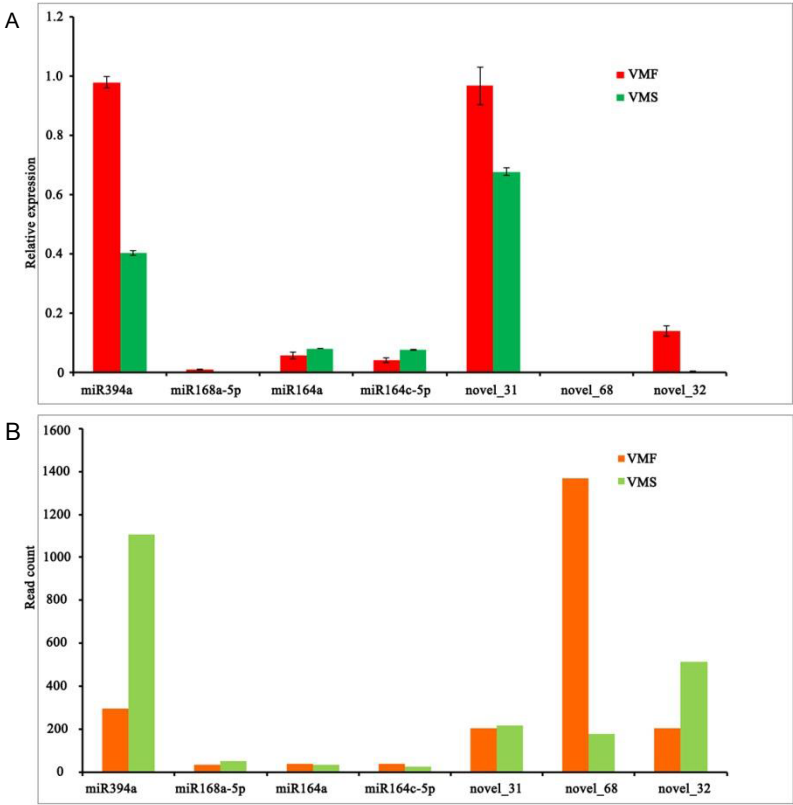

Supplement: Supplementary file 20 — miRNAs have opposite tendency between qRT-PCR and sRNA sequencing. (PDF 420 kb) [file 12864_2017_4180_MOESM20_ESM.pdf]
