# Supplementary material for: miRNAs involved in the development and differentiation of fertile and sterile flowers in Viburnum macrocephalum f. keteleeri
Source: BMC Genomics. 2017 Oct 13;18:783. doi: 10.1186/s12864-017-4180-x (PMC5640959; doi:10.1186/s12864-017-4180-x)

Additional file 8. Fold-back structures for novel miRNA from *Viburnum macrocephalum* f. *keteleeri*. Precursor sequences for novel miRNAs were shown in black letters with miRNA sequences highlighted in red. Precursor secondary structures and MEF value were produced using the MFOLD (http://mfold.bioinfo.rpi.edu/) software. Precursor sequences for novel miRNA*s were shown in black letters with miRNA sequences highlighted in green.

novel_1 MFE = -44.50 kcal/mol

GGAAUGUUGUCUGGCUCGAGGACCCUUUACUGCUUUAUCAAUUUUCUGAACAG
AUCUAUUUAUGAAAAAUAUACAUACAUGCAGUACAUAUAUAUAUAUAUUUCG
UUUGGUCAUGGUUUAGUGUUGUCGGACCAGGCUUCAUUCCCU

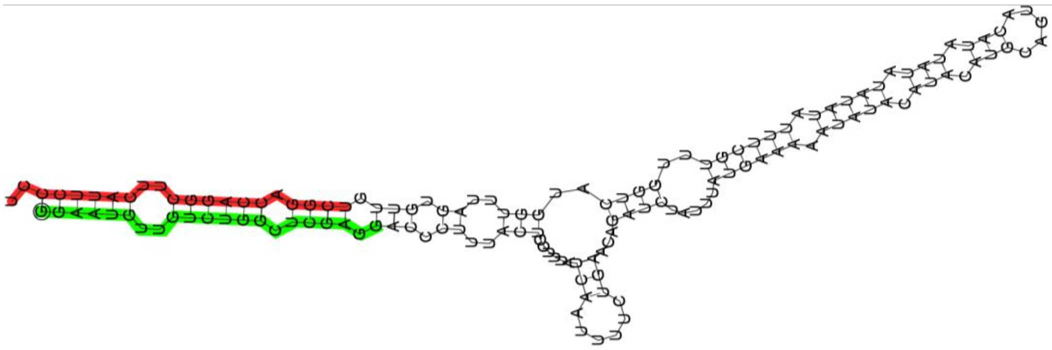

novel_10 MFE = -34.60 kcal/mol

GGUAUGGUGGGCUUGGGGAGGAAAGUAUUUAUAUAAAAUAAAAACCCUUUCC
AAGACCCCCCAUACCGU

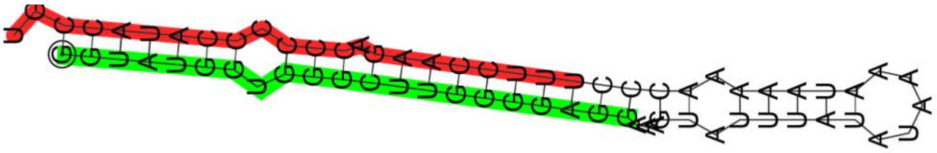

novel_100 MFE = -32.90 kcal/mol

GGUGAUGGAGGUUCUAAAACCUAGAAUCAAUCGCGGGGUGGGGAGAGGGUAG
GACGAUUCUGACUUGUUCAUAAACGGUUUGAGAACCUCUGUCAACGC

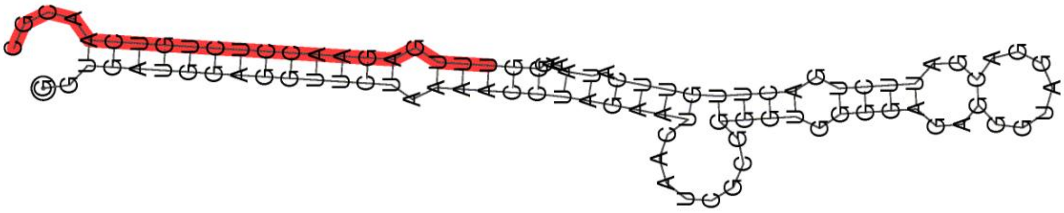

novel_102 MFE = -35.70 kcal/mol

UUUUUAUUAGACUUUGGUACUUCUUUAGUCACUGAUGAUUAAAAUUGUCACU
AAAGAUAACGUUCAGUGUCAUUCAGUGACAAAAAUGAACGAAAGUCUAAUAA
AAAAU

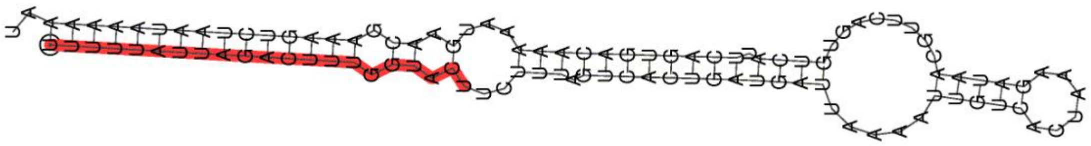

novel_103 MFE = -50.70 kcal/mol

AGUAAUUCUACGCUGGCUUUUGACAGAGACUCGCUAAUAUGACAAUAAACUAU
AGAAGCAACUAAGUCGUGGACCAAACACCAAUCACUGCAGUGUUGUUAUGUUA
UCGAGUCCUUUGUUUCAGUGUAGAAUUACUCU

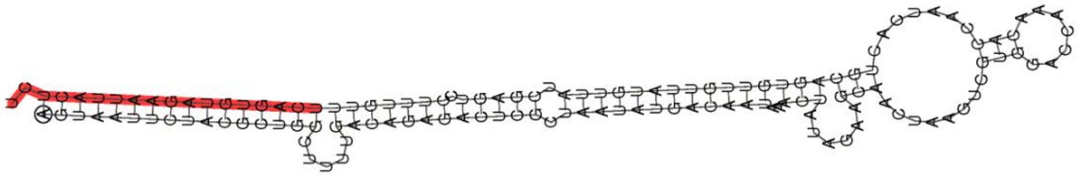

novel_105 MFE = - 23.60 kcal/mol

ACUGGUGCAGUGUAGUGUCUGGCCCGUAUGUUUUCAUCAUAAGAAACAGAUUC
UACGGCUACGUGACACAUGUUACACCCGUGU

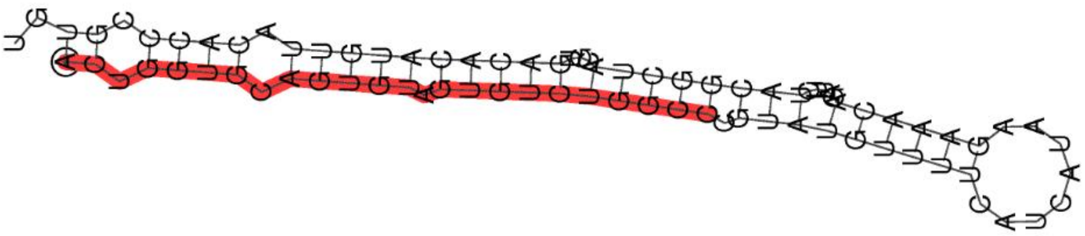

novel_107 MFE = -12.00 kcal/mol

AUAGUUGAAGAAUAGAUCUGCACCAUUGAAGAUAGAUCUGUCGUCAAUUUUG
C

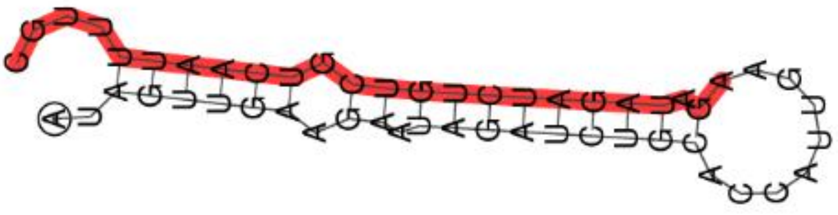

novel_108 MFE = -36.00 kcal/mol

CAUUCAAUGGACAUGUGAUACAGAUGAGAUUACAGACCCAACUUAUAAUCAAU
CAUGAGGAAAAGAAUAUGAUUUUGGGAUUUUGUAUUACACCCGAGUCGUGUG
UCUAUUGAAUGUGAAGU

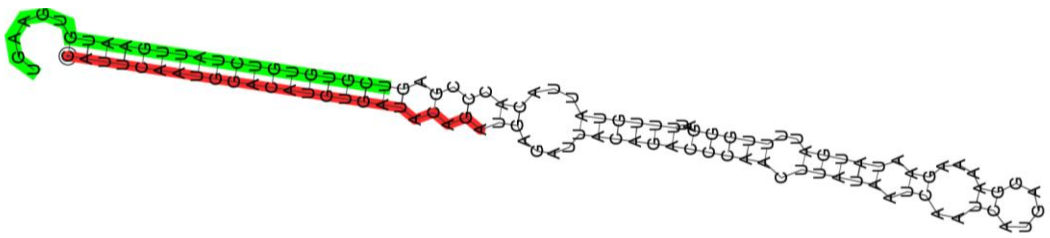

novel_110 MFE = -48.29 kcal/mol

UUAAGAUCUGUCCAAGUGAUGGAGUCUACGUCAUAAUUUGUGUCAGAGGGGG
CCUGCGUUCGCGCGGCCCCUACUCAAUUAAUAUUUCAAAUGUUCGUGGUCUAG
GCAAUUAUAUCUCCUUCAUUUGGAAGGACUUAUG

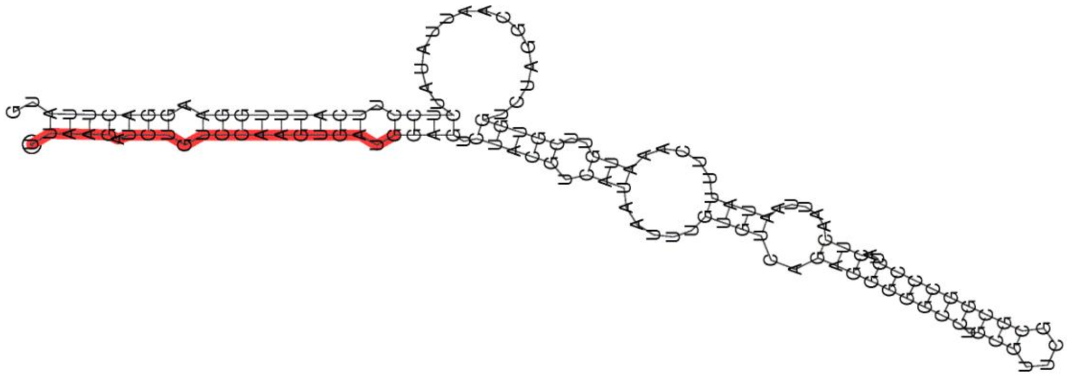

novel_112 MFE = -36.10 kcal/mol

UUGGAUCUUGGAAAGUAUAGAGGAAAAUGCGAAGGAGAGGGAAAAAUAAGUA
UUUUUUGUAGCAUGUUUUUUUCCCUUUCAUUUAGUCUUUCCCUAUACAAUCUG
AGAACAAAC

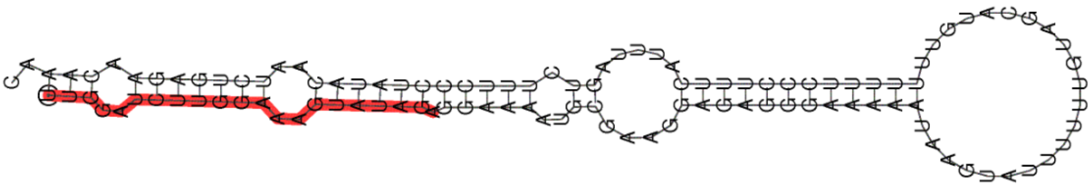

novel_13   MFE = -29.13 kcal/mol

GGAAUGGGAGCGUGGGAAAGAAAAUUACGAGGGAAAAUGAUUUCUUUCCUAC
ACCCCCCAUUCCGC

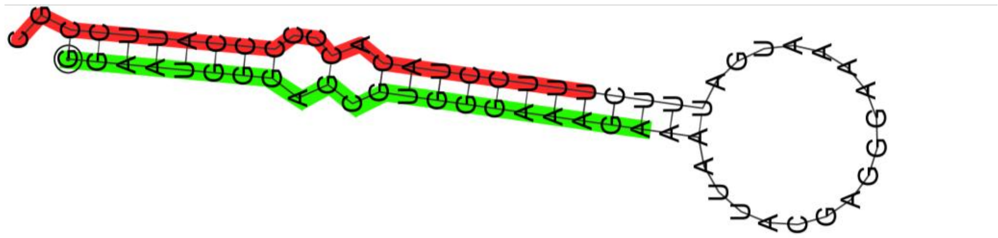

novel_14   MFE = - 38.43 kcal/mol

UUCCACAGCUUUCUUGAACUUCUUCUAUCUUAAUCUAAUAAUCUCUUGAAUAC
UCCGCAUAGAAGAAGCUCGAGAAAGCUGUGGGAAA

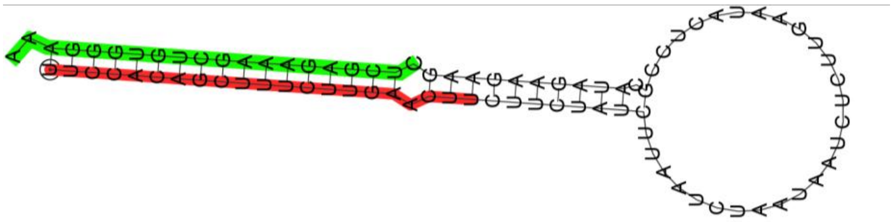

novel_16   MFE = -42.30 kcal/mol

AGCUUGUGCGUGAAUCUAACCUCAUCACAUCACAGCCAUCCAUUCUUUCUGUU
CACUCCAGAUCUCGAGGCAUGGCCAAUCAGAUCCGGUGAUAUUAGAUUCACGC
ACAAACUCG

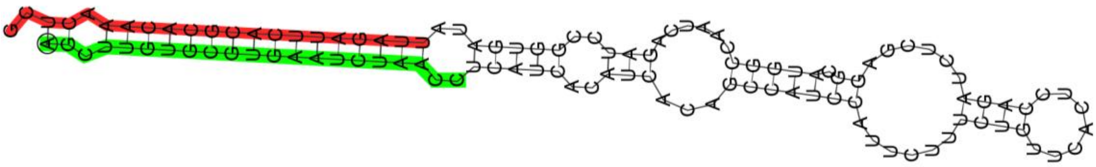

novel_17   MFE = -27.30 kcal/mol

GGAGCGACAUGAGACCACAUCAAUCCUCAUUUAAGUAGGUGUUUAUGUGUUCU
CAGGUCGCCCCUG

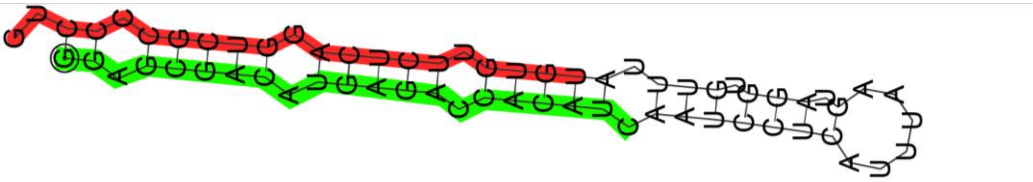

novel_19   MFE = -35.30 kcal/mol

GGAAGUGGGAGGAUUGGCAAGGGAAAAUAAAUACUCUGUUUGGUACUCCUUG
CCCAUUCCUCCCAUUCCGC

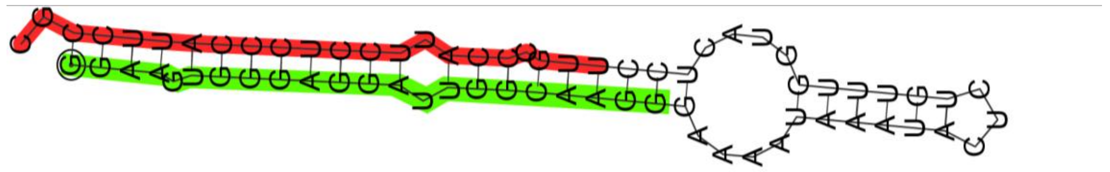

novel_20   MFE = -33.60 kcal/mol

ACGUUAUUCCUCUCCAUAAUAUUUAUCACGGUCUCUAGUAUGGCUCCAAAAGC
UAUCAUCAUAAAUAUUAUGGAGAGGAACAAUGUUG

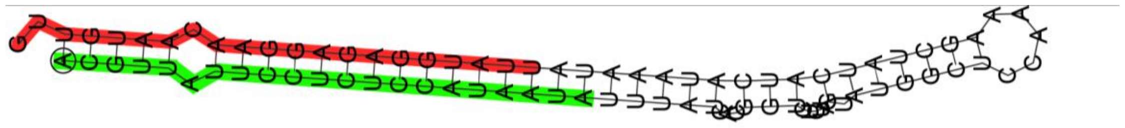

novel_23   MFE = -32.70 kcal/mol

UUAUACAGAGAAAUCACGGUCGCUCGAGUUCUUUCCUCUCUCGACCGGGUUUC
UCUGUAUAAAG

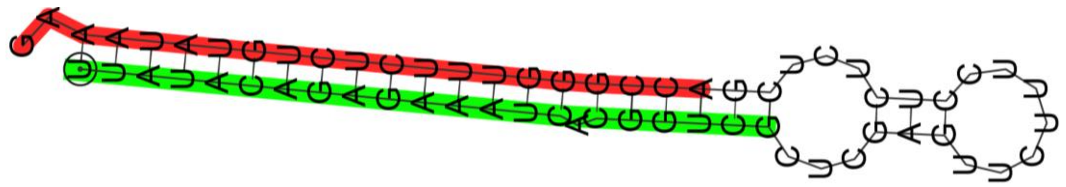

novel_24   MFE = -41.00 kcal/mol

AGUCAUGGGAGGUCUUGGUGGUGGGUUGGCAGUGACCGUGUUAACGUUAGGA
GACGAGCUGGCAGCGGCCUUCUUGGCAGUGAACUGAUUUGGCGUUCUCUUAGC
UUCUUCCACAGCUUUCUUGGACUC

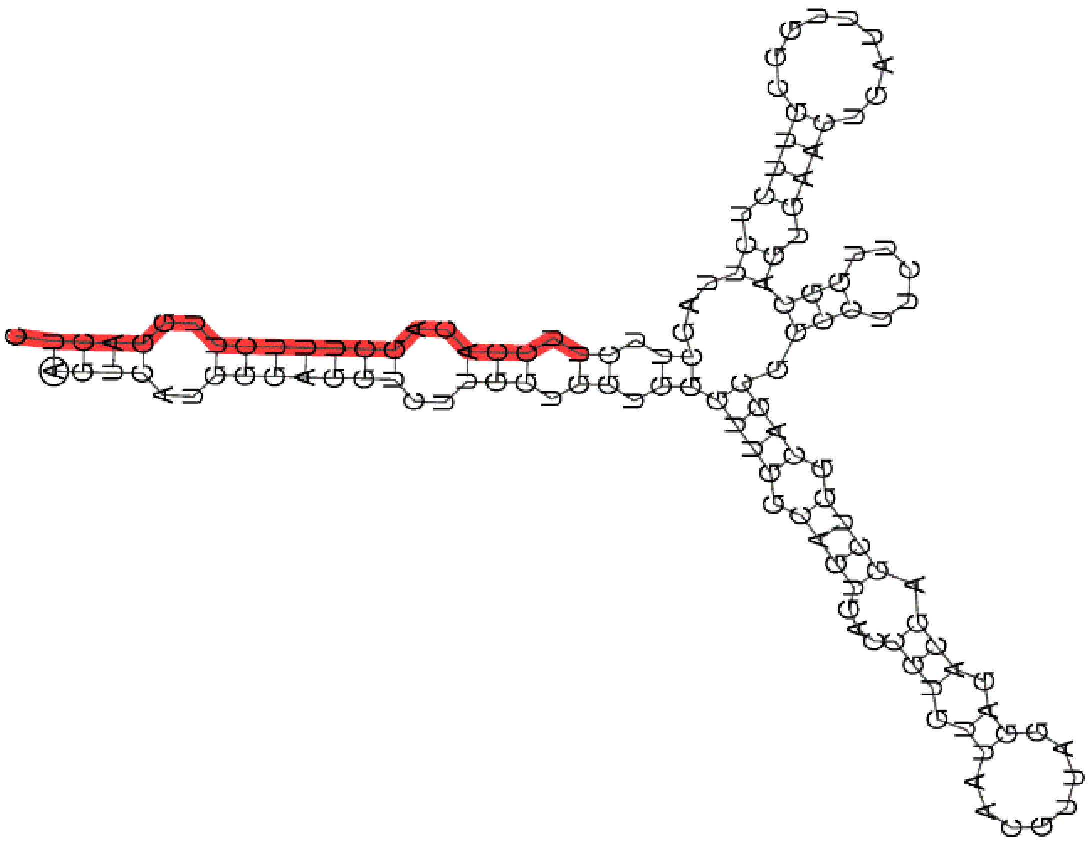

novel_25   MFE = -54.30 kcal/mol

AGGCACUUUCCCAGUCGUCUCUGCUAUUGUGGUGAGACAUACACGCCGAUGUG
UGUGUGUAUAUAUGCCACCAUAUUCGUCACAGAGACGACUUGGAAGUGCCAGA

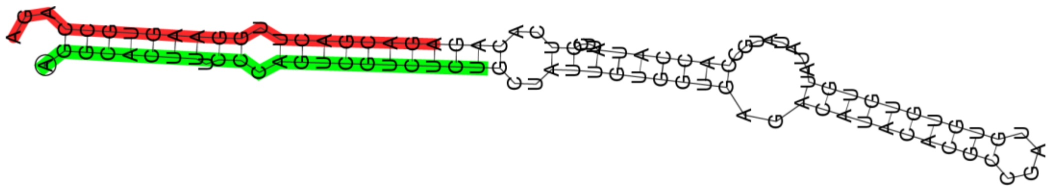

novel_27   MFE = -36.00 kcal/mol

CGUGGUAUUGGUCCGGCUCAGCUUCUUCUGUUGCAUAAAUAGUUAAGAGAUG
AGCCGAACCAAUAUCACUC

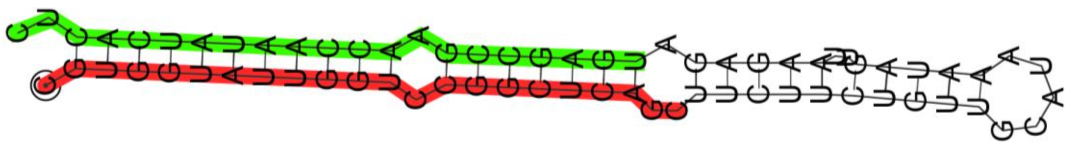

novel_3 MFE = -76.54 kcal/mol

GAGCUCCUUGAAGUCCAAUAGAGGUUCUUGCUGGGAAGAUUAAGCUGCUGAU
UUAUGGAUCCCUUAAGCCCUAUCCCAUCUUUAAUUUAUGGAUAGGCUUAUGGC
UUGCAUAUGUCAGGAGCUUUUAUUACCCAAGUUAGAUCCUUGUUUGGAUUGA
AGGGAGCUCU

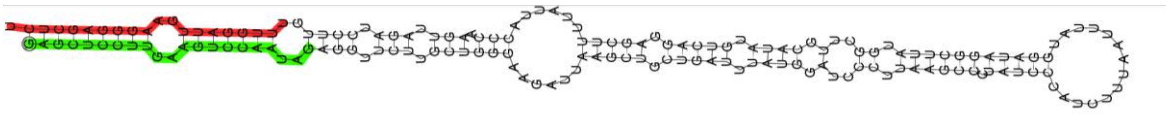

novel_31 MFE = -30.70 kcal/mol

UACAGAUGGGUAAUCGACAUUGUGGCAACUACUGUUUCCAUAAAGCCGUUGAA
UCACAUUGUCGAUAACCCCUUUGUAC

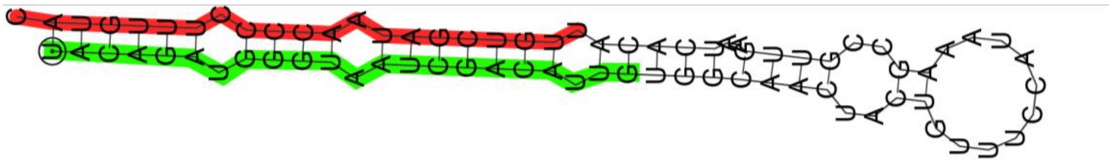

novel_32 MFE = -22.20 kcal/mol

AGUGCAUUGGACUAUUGUACCUUGUCGGUUUCGUAUCAAAUGUACAAUACU
UCAAUGCAUUUG

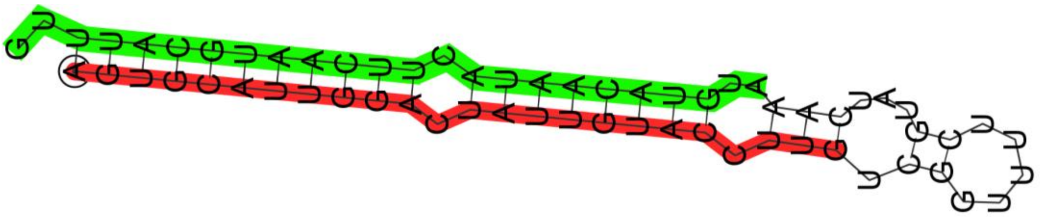

novel_37 MFE = -71.70 kcal/mol

UCGCUUGGUGCAGGUCGGGACGGAUUCGCCGGCGAGGUCAUUUCCUUGUUGUU
CAUGUGCAAACAUAUAUGUACAUAUGCAUAUAUGUAUUUAUGUGUAUGUAUA
UCAUGUACACGUGAUUUCGUUGUAGGUGAAUAUUGUCCCGCCUUGCAUCAAUU
GAAU

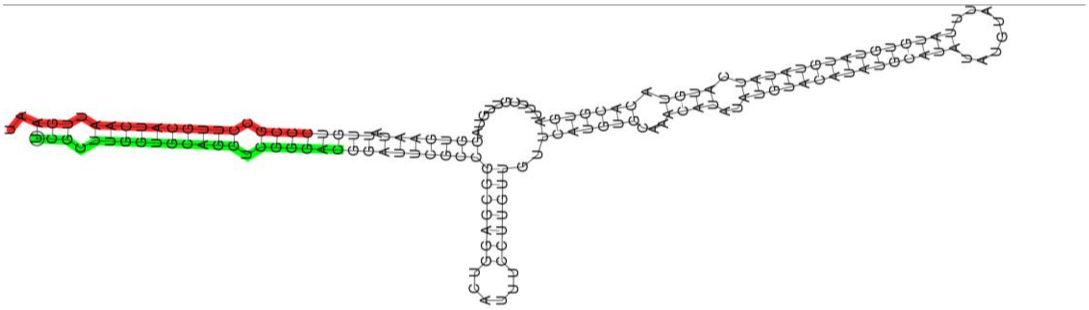

novel_38   MFE = -32.00 kcal/mol

UUUUGUUGAUGGUCAUCUAAUCAUCUUUGACACCUGACAUGGCAUUAUGGUU
AGAUGAUCAUCAGCAAACA

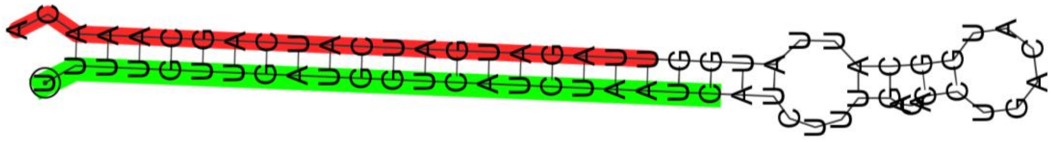

novel_4   MFE = -32.60 kcal/mol

AUAGAUUAUUUUCAGCGAAUUACAGAUUAUAGAUUAAAUUGUAAAACUAUUA
UAAUUUAACGUAUGAUUCAUAAUCGACUGAAAAUAAUCUAUUC

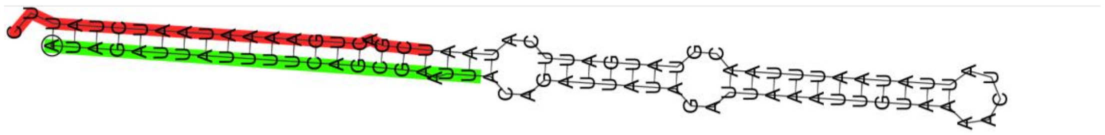

novel_40   MFE = -32.90 kcal/mol

ACUCUCCCUCAAGGGCUUCUGUAUUAGCCUCUAUUAGAAGGUCAAGACUGAAG
GUCUUGUGGGGAGAGUGG

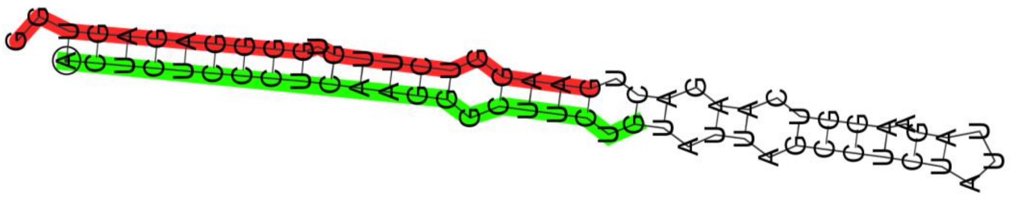

novel_41   MFE = -26.44 kcal/mol

UGAAGCUGCCAGCAUGAUCUUAACCUCCCUACUUUGUUGAGGAAAGAUCAGAU
CAUGUGGUUGCUUCACC

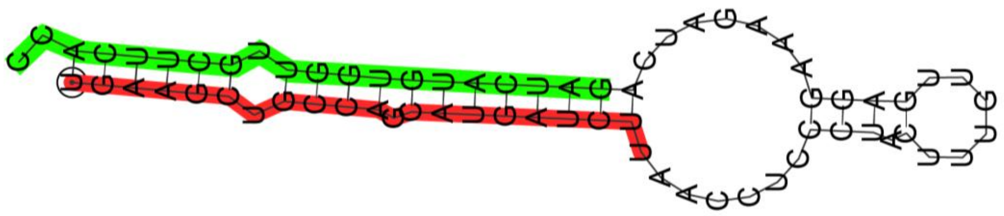

novel_45 MFE = -26.50 kcal/mol

UUUGAUACGCACCUGAAUCGGGAAAACAUAUAUUUACAUCUAUGCUUUAUA
UAAAUUUAUGUCGAUUUAGGUUCGUAUCGAAAUU

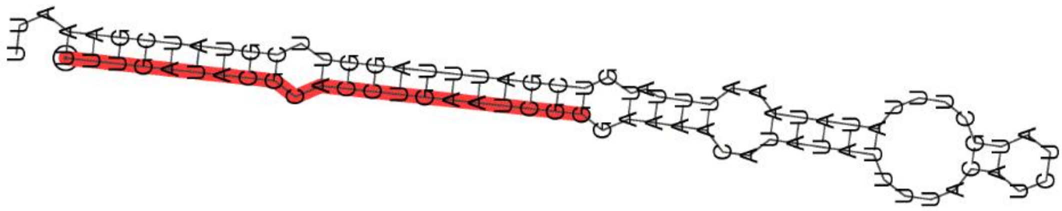

novel_46 MFE = -47.80 kcal/mol

UUUGGAUCUCGGAAGUAUAGUGGGAAAAUGAGGGAAAUGAAAAAAUGUACUA
AAAUGCGCAGUACAUUGUAGCAUAUAUUUUUCCCCUUUUUUGUGCAAUUUCC
CUCUAUACUUUCCAAGAUCCAAAC

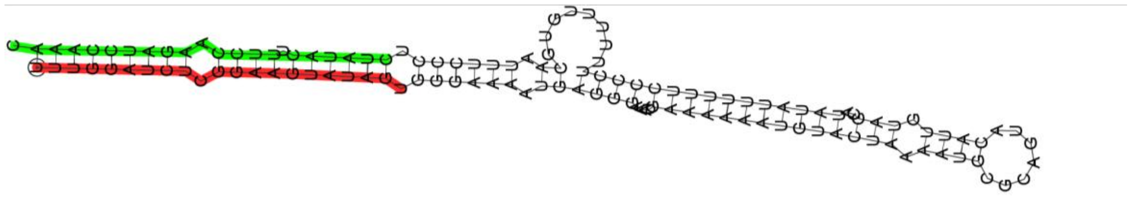

novel_48 MFE = -91.50 kcal/mol

UCAGUUCAGUCAAAGGGCAGACUAUCCACGAUUCGACUAGGCAAGUCACGGGA
GACACUUGGGGCUGGCUAGAUUGUAGAUUAGUGUGCUUUUUCAAUGGAAGCU
GAAACUUUACAAAAAAUAAAACACUUGGGCUAGACGGGUGGAGUUGGUAUGC
CCCGUGAAUCCCCUAGUCUCUGCGUGGAUAGUUUGUCCUUGACGGACUGAAG

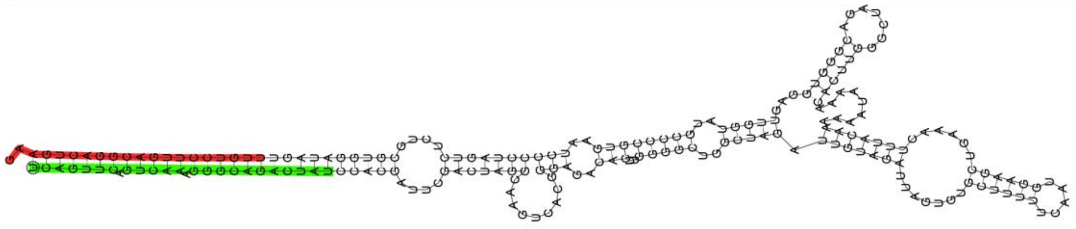

novel_50 MFE = -59.30 kcal/mol

GGAGCAUCAUCAAGAUUCACAGAGCUAUCUAGGGUUUCAUGGAGAGGGGUAG
UUUGGGAAUGAUAGUGCCUUUUUGGUCUCUUUACCCUUUACUCCCUCUGGCCC
UUUGAUAUGGGAAUCUUGAUGAUGCUGCAG

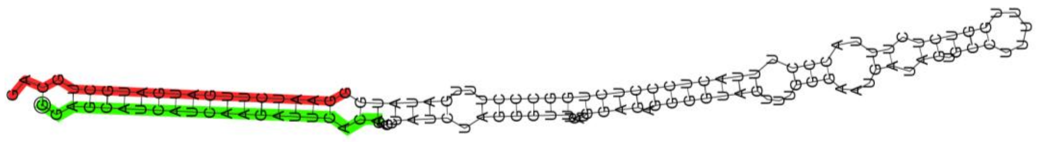

novel_52 MFE = -41.80 kcal/mol

ACGUGGUACUUCAGGACCAAUAAACAAACAACAUAUCAAUGAUGUCCCCCUAUUUGCAGGGUAACCACUACCCCACUACUACCACAACAUCUUUUUAUUGGUUCUGGAGUACCACCUGGC

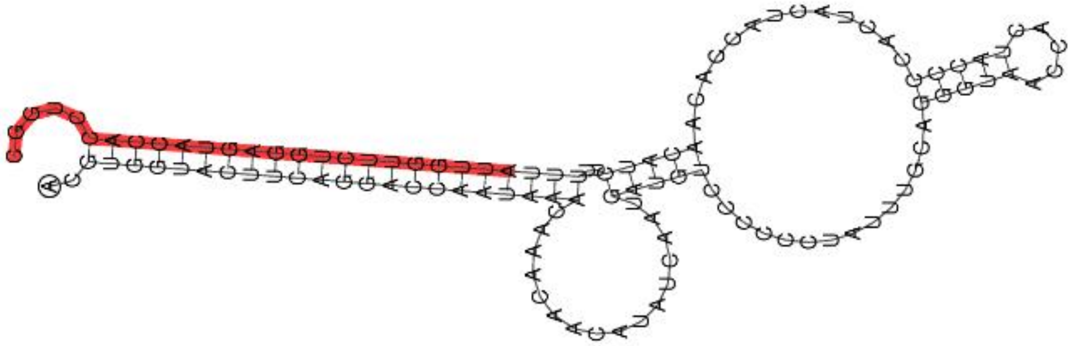

novel_53 MFE = -54.10 kcal/mol

UUUGGAUCUUGGAAAGUAUAGUGGAAAAGGUGAGGGAAAGGAAAAAAAUGUAUUAAAAAGUAAAAUAACUUUUAGGGUAUUGUUUUCCCUUUCCUUCACGGAAUUCUUUGUAUUUUCUGAGAUCCAAACAU

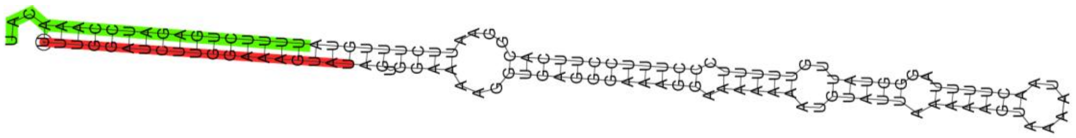

novel_55 MFE = -61.30 kcal/mol

AACUGACAUUUUUAUCUGACCCACUGAGUUUCAGCAGUGCAUUGGUCGGUGACUUGGUGUUUUUAGUAGACCCAUGUGGACCCAACACGUUAACCAAUACAUUGCUGACAACUAGCGGGUCAGAUUAAAAUGUCAAUUUU

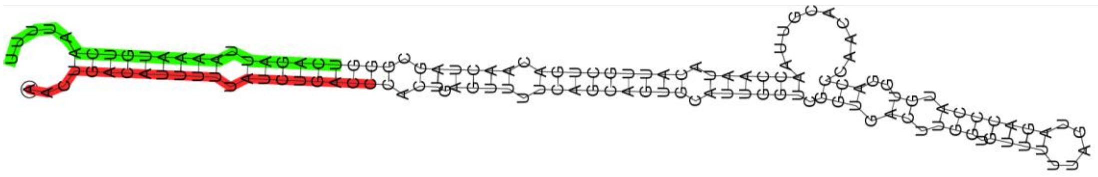

novel_57 MFE = -74.60 kcal/mol

UGAUCUUCACUCCAACAAGCUUUUGGGACAUUUAGGGACAAAAACAUGGUCUCUCCAUCUGAUUCAAUUGGAAAGUUGAAGCAGUGGAAACAAAGAUGGGGUCAAUAACAAAGAAAGGGCUACAACAAUACAUUUUGCAGCUUCAGUAACAUCCAUUGAGAACUAAGGAGACGUAAAUAUCCCCGAAAGCUUGUUGGAGUGAAGAUGAAG

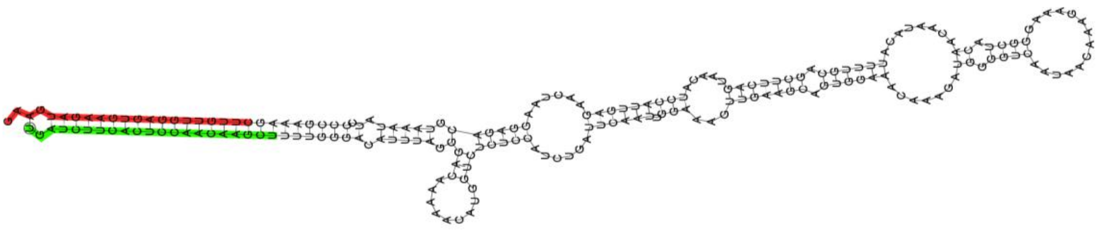

novel_59 MFE = -52.80 kcal/mol

UUAGUUCACGAGUCUGAAGCUCUGUUUUGUGAAUGAACACAGAACAGAGCUU
ACACAGAACAGAGCUUCGGACUCGUGAACUAAAG

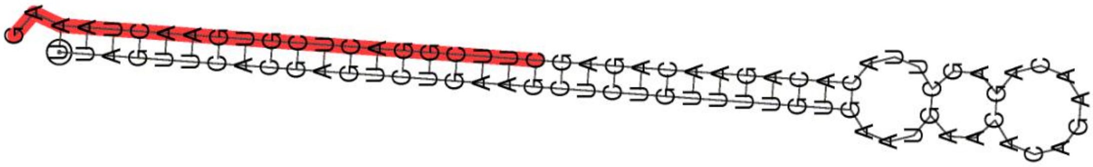

novel_6 MFE = -35.60 kcal/mol

GGGAUGUUGUCUGGCUCGACGCAUUCACUGUACUUUUCUAGAGAGAGAAAGU
GAAGGUAUCGGACCAGGCUUCAUUCCUC

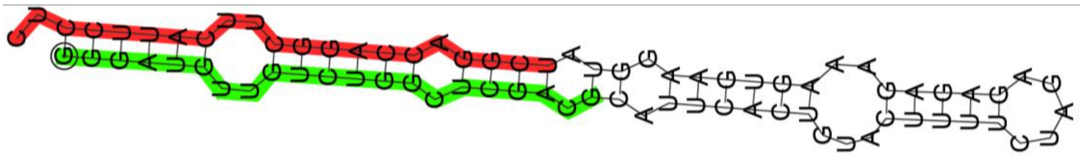

novel_60 MFE = -56.10 kcal/mol

AAGCUCAGGAGGGAUAGCCCCAAUGGAUGACAAUAUUAAUGGCGGCGGGCGGU
GGCUGUCAAGACAGUGUGGGUGUGUUCUAACAUCGUCCGCAGGGCUAUCCAUC
CUGAGUUUA

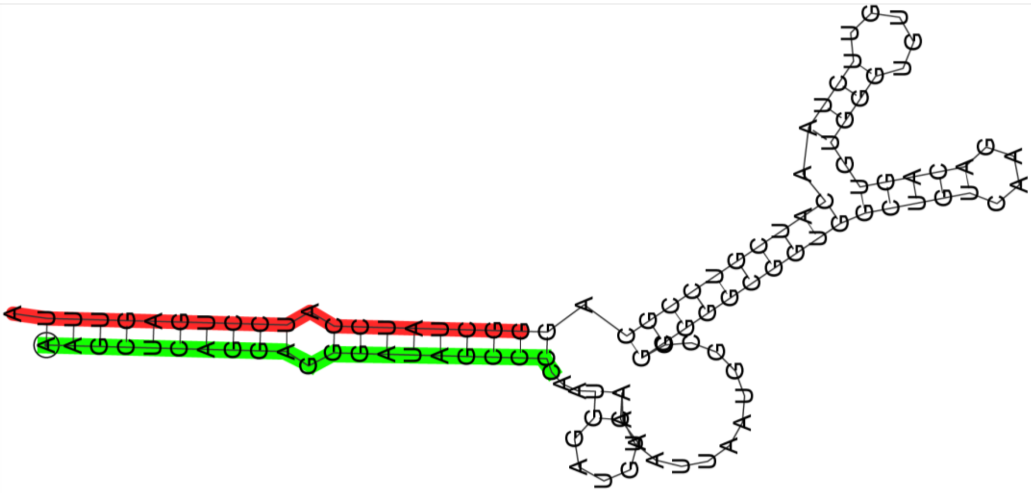

novel_61 MFE = -32.20 kcal/mol

ACCGGAUGAUGUCAACAGUUGGAUUAGAUGUAACCAUUGGAUGGGGAUCUAC ACAUAAUCCAACGGCUGACGACAUUUGGUC

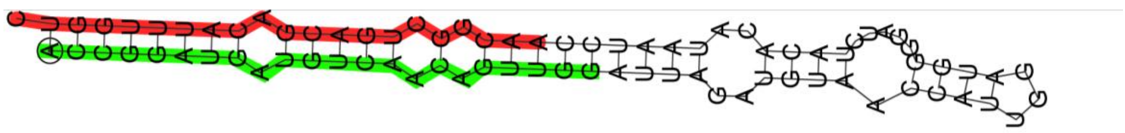

novel_62 MFE = -51.04 kcal/mol

CAAUAAUGCUAGACACACAUAUGAUGCACAAAAAAUUAGUACACAAAAUGAU GCGUCAGGUCAGAAAUUGAGUUCUACCUCAUUACCUGGUCAAUAAAUUCACUA ACACACAAUGUAGCUUACUAAGUUUUGUGUACCUUUUGUUUGUCUAGCAUU AUUGAA

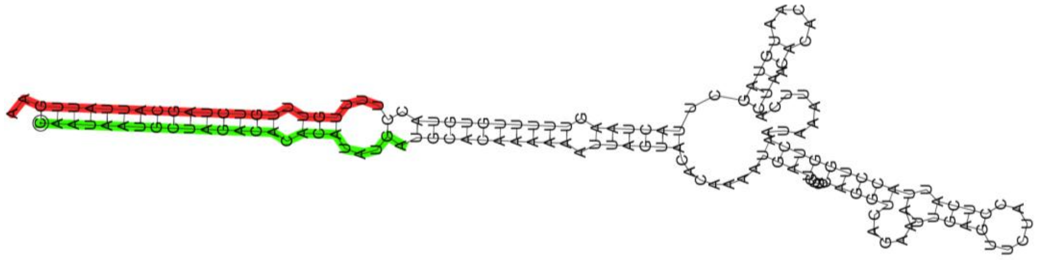

novel_64 MFE = -27.65 kcal/mol

CAUUUUUACUUUUAGCAACACUUUGACGAUUUUUUGCGACAUUUUAUUUUCAC AGAAGUGUCGCUAAAAGUGAAAAUGGA

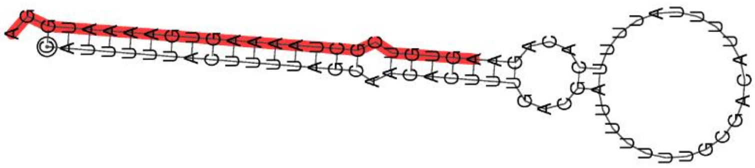

novel_65 MFE = -86.60 kcal/mol

CAAGAAACCCUCCUGAAUGAUUUUUAAUGCAAAAAAGCCCUUGUACUAUGCAU UUUGUUGCUAAGACAUCCCUAGGUGUUGCUAAAAGUCUCCAUAAGGAGGUUU UUGCGACAAAAUGCAUGGUUCAAGGGCUUUUUUGCUUUAAAAAUAGUUCAAG GGGUUUUUUUGCA

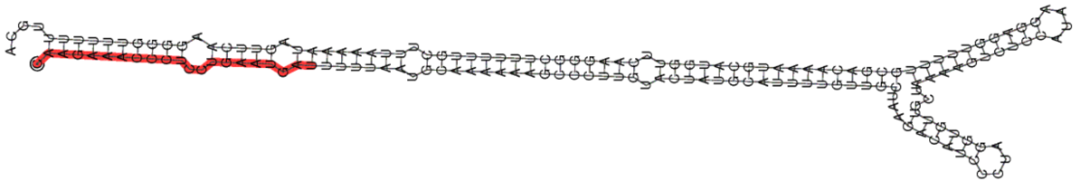

novel_66   MFE = -86.60 kcal/mol

AAAAAUGGGACUCGCUGAAGUGGCAUUAAUGAUGUGUUGGUUGGUGUUUUAA
AGAUAGACAACAGACUCUACACACUAACCAAUGCUUUGGUGCCACAUCAACGA
GUGUCAUUUUUUG

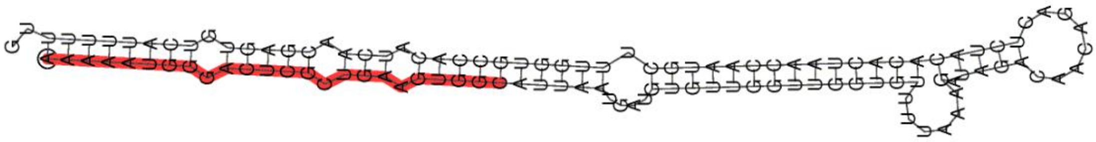

novel_67   MFE = -101.50 kcal/mol

ACCGAACAGCUGCGGAGAUAAGCCAAAGCCCUGAUAAGAAACAAGGCAUGACA
UAGGAUGGUGGCACAUAGCAUAGCAUUAUCCAAGAGGGGCCAUACCAGUAAUA
UGGCGUGACACCAUCCCAUGGCAUGACUAGAGCUUCUUAUUAAGGUCUUGUUC
CUUAUCAGGGCUUUAGUUUAUCUCUGCAGCUGUUCGAUUAUGCGC

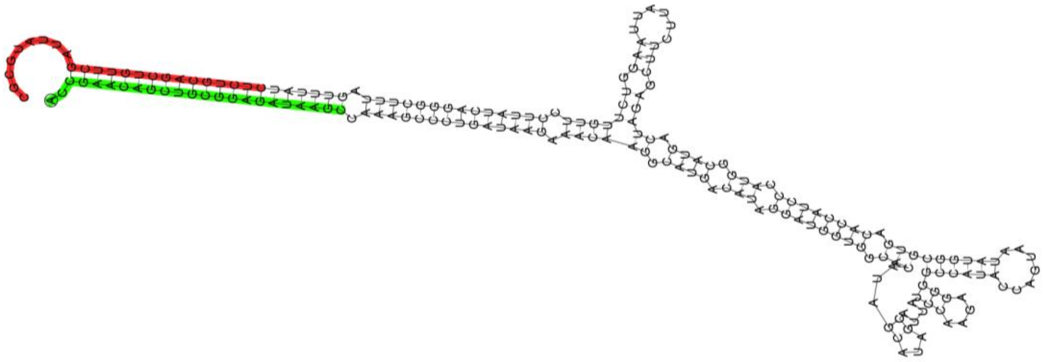

novel_68   MFE = -82.30 kcal/mol

GAGCUUCCUUCAGUCCACUCAUGGAUGAGGAAAGGGGUUGAAUUAGCUGCCGA
CUCAUUCAUUUAACCACUCAGUAGAAAAGAGGAUAAGUUUUGUGCUACUGUG
ACUGAGUGAAUGAUGCGGGAGAUAGUUGUCCAUCCCUCUCCCUCUGUGCUUGG
ACUGAAGGGAGCUCCCUUU

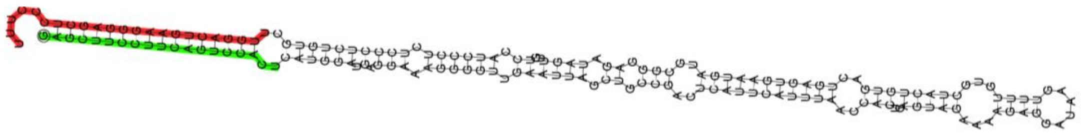

novel_69   MFE = -41.10 kcal/mol

UGGUGGGCAGUGUCCGGCGGGUGGGCCUCCAUCCUGGGCCUCUCCGGCAAAAC
CCACCGGCCGUCACUGCUCCUCACC

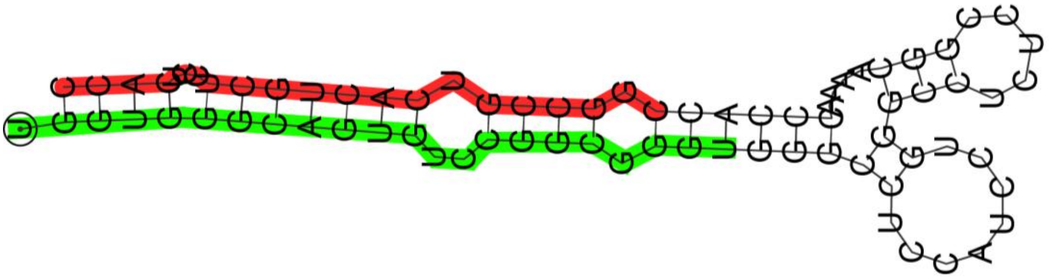

novel_7 MFE = -37.70kcal/mol

AAAUGUCGUCUGGUUCGAGAUCUUCCGUGUGAAUCUUAAGAAUUCAUCAUCGG
AAUGAUUUCGGACCAGGCUUCAUUCCCC

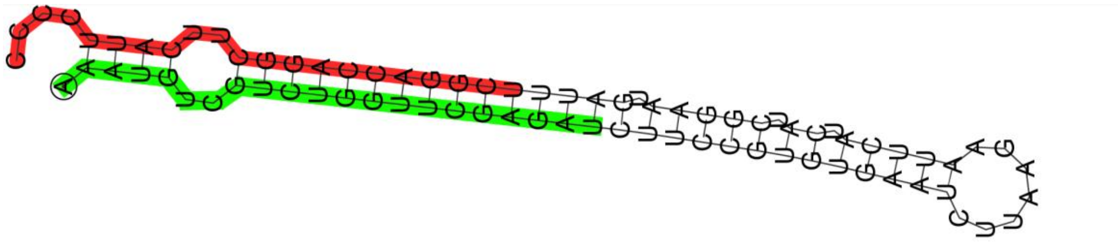

novel_71 MFE = -70.20 kcal/mol

AGUAAUGCGAAACUUACAAAAAAAUGUUAUAAAAAGUGUUCCACAAACUGAU
GUGGCAGACAUUUAUUGAUGAUUAUAUGUGAGUCUUCUUUUAAUGAUCAAUA
AAUGUGUGCCACAUGUGGAGUAUUUUUUGUCAAUAUUUUUUGUAAGUCUAGC
AUUACUCU

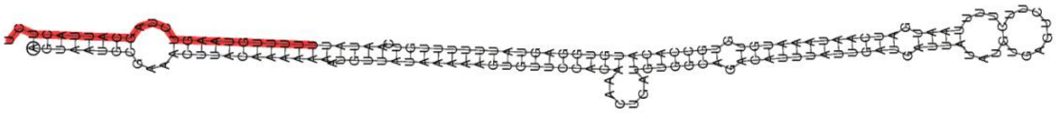

novel_72 MFE = -28.60 kcal/mol

AUGGAUGCGUUUGGCAAUACGAUUGGUUAUUUCCCUUGUGGAAAGGAAAAG
AAAAGUAAUGGUGUUGCCGACACCACCCAUUCCGA

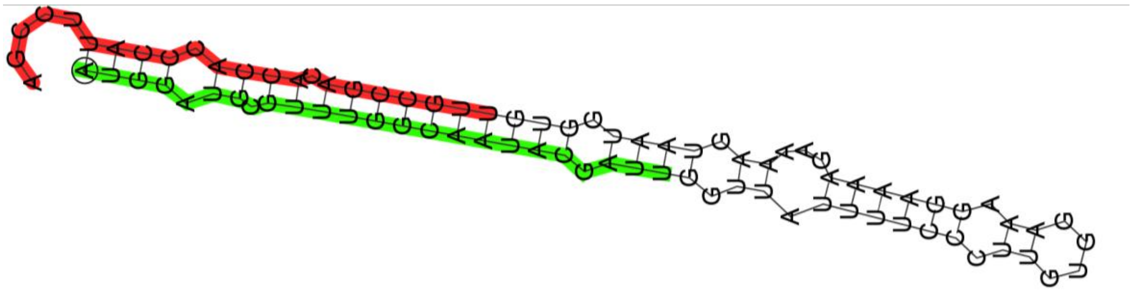

novel_73 MFE = -12.40 kcal/mol

GAAGAGGCGUACAGUUCUUCAGACUGAGGGCUCUUUCC

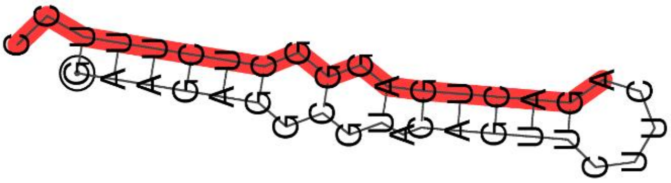

novel_77 MFE = -26.20 kcal/mol

UUUGAGCCUCAAAAUAGUAAGAACAAGGGUCUUGUACGAGUAUGUGCCCUAU
UAAUUAUAUGUCGGAAACUUGUUCUUACUAAUUUUAGAUUCUUAAC

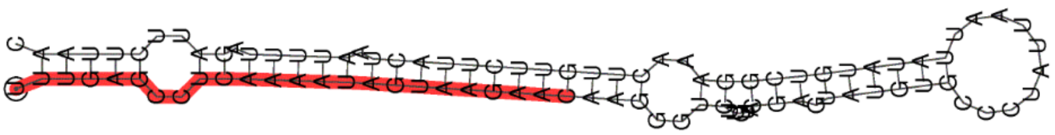

novel_78 MFE = -47.11 kcal/mol

AGUUGCUCACAAAAUGAUGUAGCAGUUGUUGUUAUUAGUUGAUCGGCAGUGG
AGUGUAGCGCACUAACUGCCCAACAAUUUGUCACAUUAUUUCGUGAGCAACUC
UUUGUG

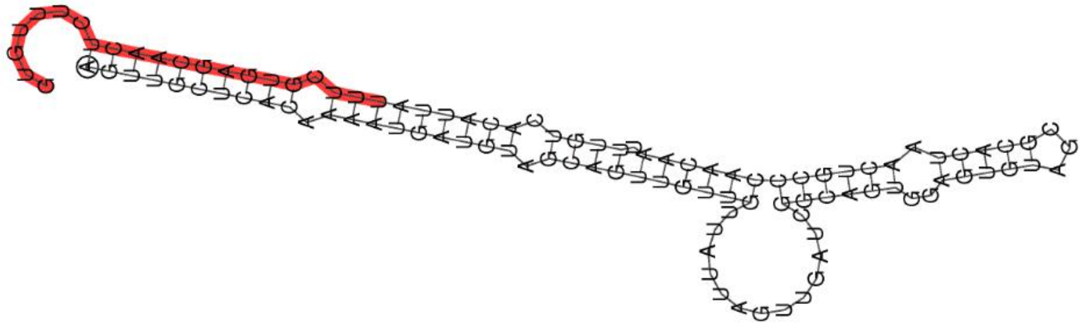

novel_79 MFE = -71.10 kcal/mol

UGACAAUAUCAUAUUUAGAUACAACCCCUAGAAUGUUUAAAAAUUAGACUCCC
CCCCACCCCUACAAACAAAAAUAUGGAUUCUGAUUGUGUAUUCUAUGAUUGUU
UAAAGUGAAAUUCAUUUGUCUGUAGGGGUUGGUCUAAUUUUUUAAGCAUCUG
GGGGUUAUGUCUAAAUAUGCUAUUAUCACA

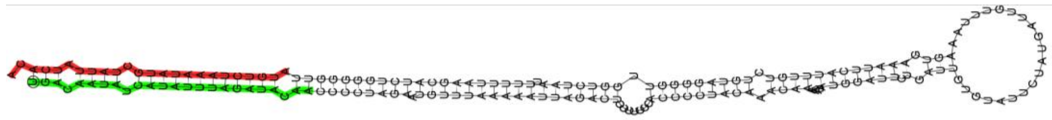

novel_8 MFE = -31.30 kcal/mol

UGUGGGUUGUUGGGCAAGACCAUAGAAACAACGUUGAUGGAGUAUUUGGUCU
UGCCUACACCGCCCAUACC

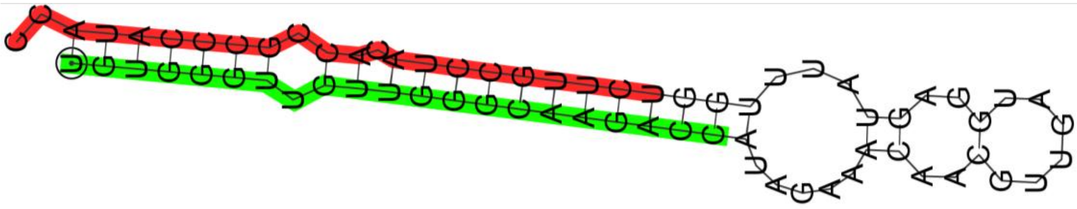

novel_81 MFE = -48.30 kcal/mol

AUUUUCAUAAUCAUUAGACUUAUUCCCUAUUGAGGGAGCAUCUCAAAUGAGG
AUAAUUUAUUUGACUUAAUCCCUUGUUAAUAAUAUAAUAUUUGAGAUGUACC
CCUAAAUAAGGGGUAAAUCUAACGAUUAUGAAAACAGGGGGU

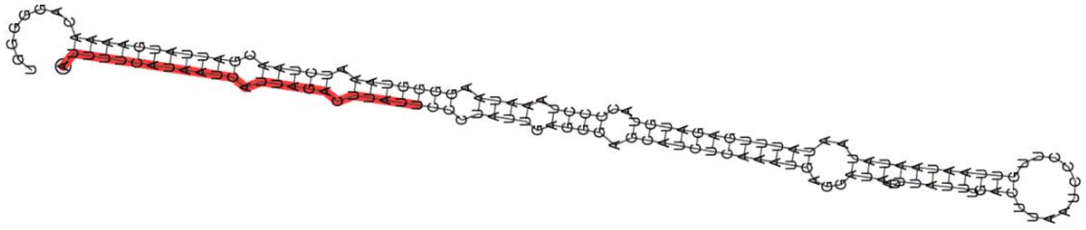

novel_82 MFE = -18.10 kcal/mol

UGCUUUGGAAUUGGAAUUGAGUCAAAGCUAAUUUUUGCUUCAAUCCAAAUCA
AAAGCUUU

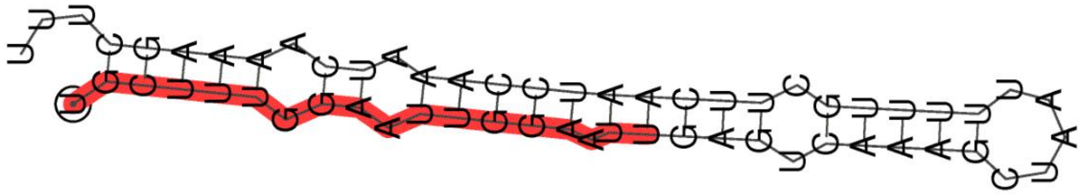

novel_83 MFE = -73.30 kcal/mol

GUAAUUCUACACUGCCUUAGAAUGAUGACUUAAAAUAAGGGACUAACUGAUG
AAGACAGUGCAUUGGUUGGUGUAUGAGGUCCAAUGAUUAUCAUUUAAUAUAU
UAAUCAAUAUACUACUGUCGUAUCAGUUAGUCUCAUAUUUUGAGUUUCUAUC
AAGAGUUAGUGUAGAAUUACUC

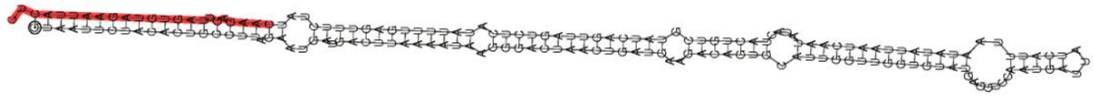

novel_84 MFE = -68.53 kcal/mol

AACGAUGACGUUGUGUAAUAGAUUAAAAGAGGAUAGUUGGAUGGGUGAAGCA
AAGGUUGAGAUUACAUCUGCAAUAAGGGGGUCAUGAAUUUAUAAAAGAGCCA
UUUGUGCUAUCUAGGCCAUUCAAAAAUUUAUCCAAUGAUCCUCUUUCAAUCGG
UUACACCACGUCAUCGUUACAACUA

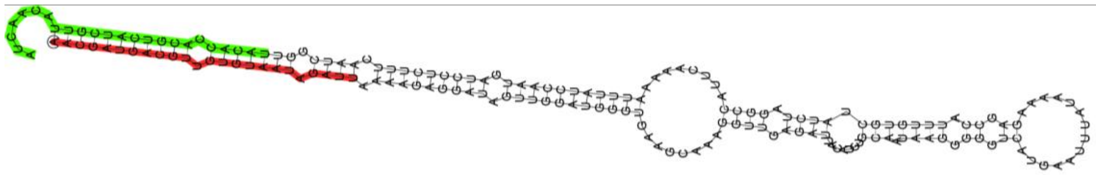

novel_87 MFE = -23.00 kcal/mol

UUGGAAUUGGAAUUGAAAUAUUUUCAGUUCAAAAGGUUCAAUUUUUUAGAUC
AAAAUAUUCCAAUCCCAAUUCCAAAU

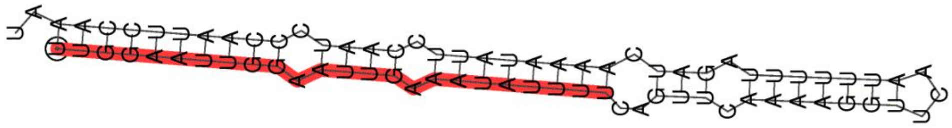

novel_88 MFE = -63.00 kcal/mol

CAAAGUUUAUCAUAUUUGAGGAUUGGCCCCCAAAAGUGUCAAAAAAAACAC
UUUUUCAAUUCUUUAUGAAGUUCUUGUAUGACGUUUUUUAAAUGACAUUUUU
GGGGGCCAACCCUCAAAUAUGAAAAACUUUGGA

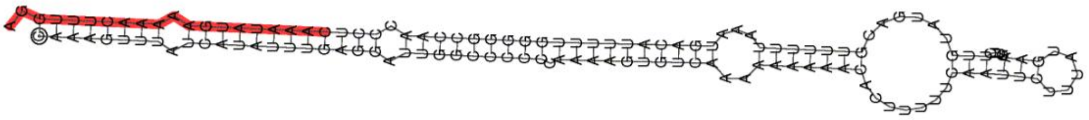

novel_89   MFE = -70.30 kcal/mol

AUCAGUUUCACAGAAUAGAGCUCAAUGAAUUCUUUUACUUUCUUUGACCUUUC
UCGAGUCCAUUUGUACACUUAAGUUCUGUACCAGGAGAAGUAGCUCAAGUAGA
UGAAGUUACACUUGGUGGUGCUUGUGUAAAUAUCAUCUCUCUCAAAUUCCAUU
UUGAAAUGUUGCUCAGUUGCCAUUGAGUUCUAUUCUGUGAAACUGAUGC

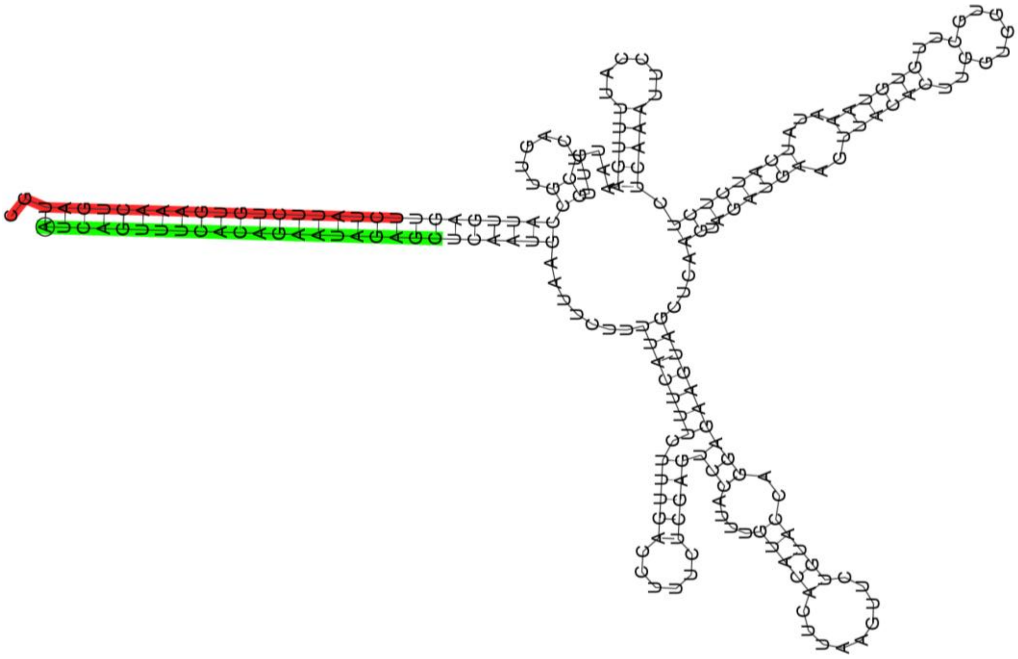

novel_90   MFE = -76.40 kcal/mol

UUGCCAAUAUCCAAACUAAAAUGGCCUUUCUGUUGGAAUCUCUCUCACCUUCC
UUCCACCAUCUGUUUCGUUGUCUGCGAGAAUGCUAGUCCUUGGGUGGCGGUGC
CUGUUUGGUACUAAAGAGGGUUGCUGCAGCAGACGUUGUGGGGUCAUCAUAG
UUGGGAUUUUUAGGUGGUGCCGUUUUAGUUUGGAUUGGCAUCU

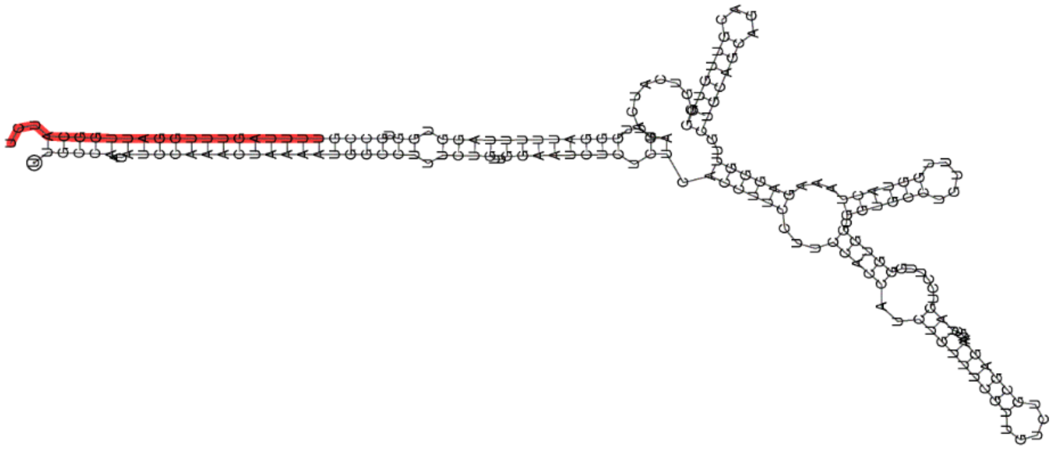

novel_95   MFE = -100.75 kcal/mol

CGCUCAUUUGUGACUUUGCAUUUGAUCCGUGCGGAUAUUCUAUGAAUGGAAU
UGACGGUGACCGUUACUCUACCAUCCACGUGACACCCGAGGACGGGUUUAGUU

ACGCGAGUUUCGAGUGUGUGGGGUCCAUUUAUGAUGACCGUGAUGACAUAGU
AGAUGUUUUGAAGAAGGUGGUGCAAGUUUUCCGGCCGGGGACAAUGUCAGUG
UCCACCACGUGCCAUAGCCUUGAAGUGUGGACACGUGUUGCCAAUGCAAUCGA
GCCACUGGGGAUGAAAUGCCGAAGUUGCACAAUGGACG

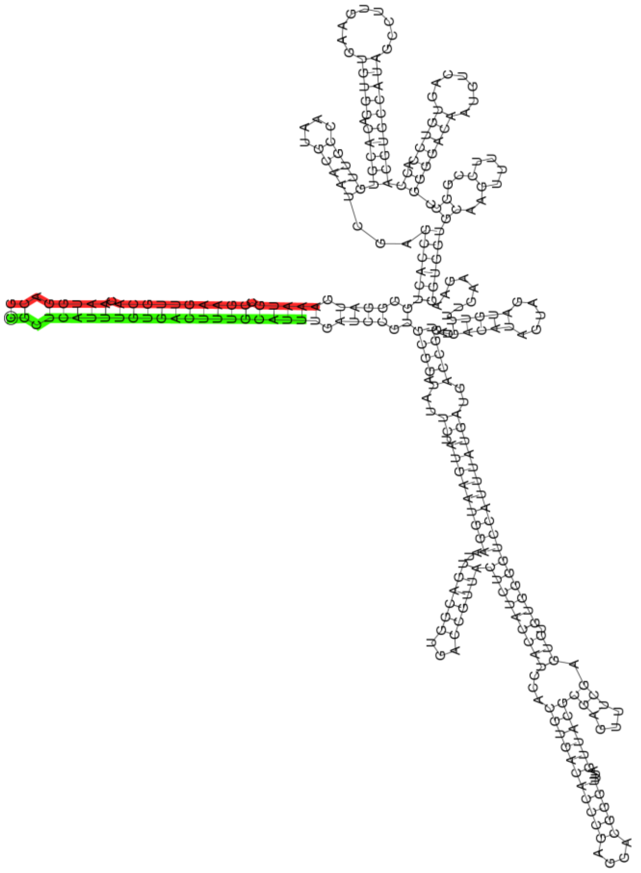

novel_96 MFE = -46.20 kcal/mol

UUUGGAUCUCGGAAUGUAUAGAGGAAAACGCAAUAUAUAUUUAUUGUACCGU
ACAAUCAAUUGUACCAUAUUUUUCCUAUUCUUGGUGUUCUCCUCUAUACUUUC
UGAGAUCUAAACA

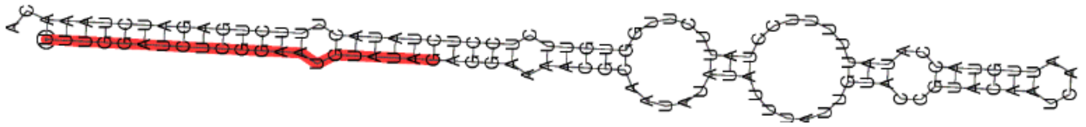

novel_97 MFE = -37.90kcal/mol

AAGCUUUGGAAUUGGAAUUGGAGCAAAUUCAGUUCAAAUUUGAACUAAUUUU
GCUUAAUUCUAAUUCCAAAGCAUGGAAU

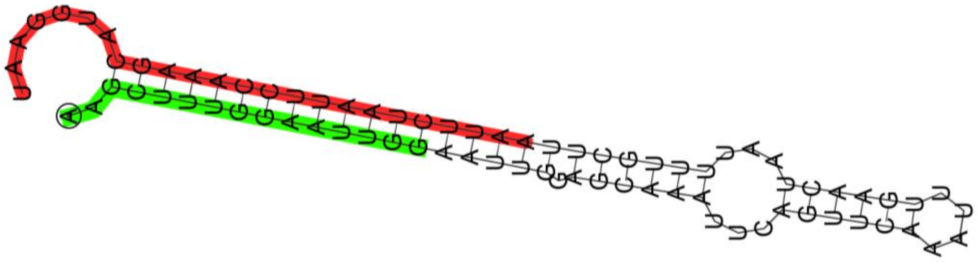

novel_98 MFE = -85.30 kcal/mol

UGCCGCACUCAUCUUACCAUGUUCGAAGUCAUUGCCAAAGGCAGUGCUAUUCU
GAUCGGAAAAAAAGGCAGUGCUAGUUACUAGUUACUACAGUGAAGUGGCGCA
GCUGGAUCACCUCCUUUUUAGGGGCGAACUAAUGGUUUUGGCAGGCAGUGAAU
AGAACAUGGUAAGAUGAGUGUGGUGGA

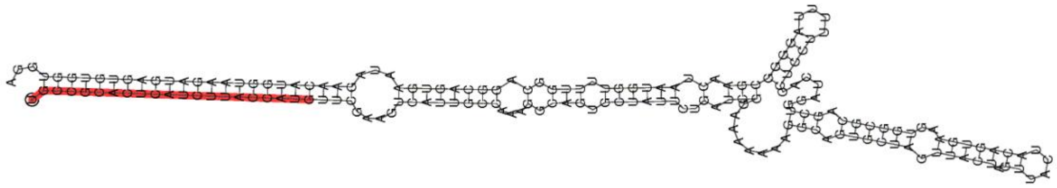

Supplement: Supplementary file 8 — Fold-back structures for novel miRNA from Viburnum macrocephalum f. keteleeri. Precursor sequences for novel miRNAs were shown in black letters with miRNA sequences highlighted in red. Precursor secondary structures and MEF value were produced using the MFOLD (http://unafold.rna.albany.edu/?q=mfold/) software. Precursor sequences for novel miRNA*s were shown in black letters with miRNA sequences highlighted in green. (PDF 2121 kb) [file 12864_2017_4180_MOESM8_ESM.pdf]
