# Supplementary material for: miRNAs involved in the development and differentiation of fertile and sterile flowers in Viburnum macrocephalum f. keteleeri
Source: BMC Genomics. 2017 Oct 13;18:783. doi: 10.1186/s12864-017-4180-x (PMC5640959; doi:10.1186/s12864-017-4180-x)

Additional file 21. Expression changes of miRNAs at different developmental stages between VMF and VMS.

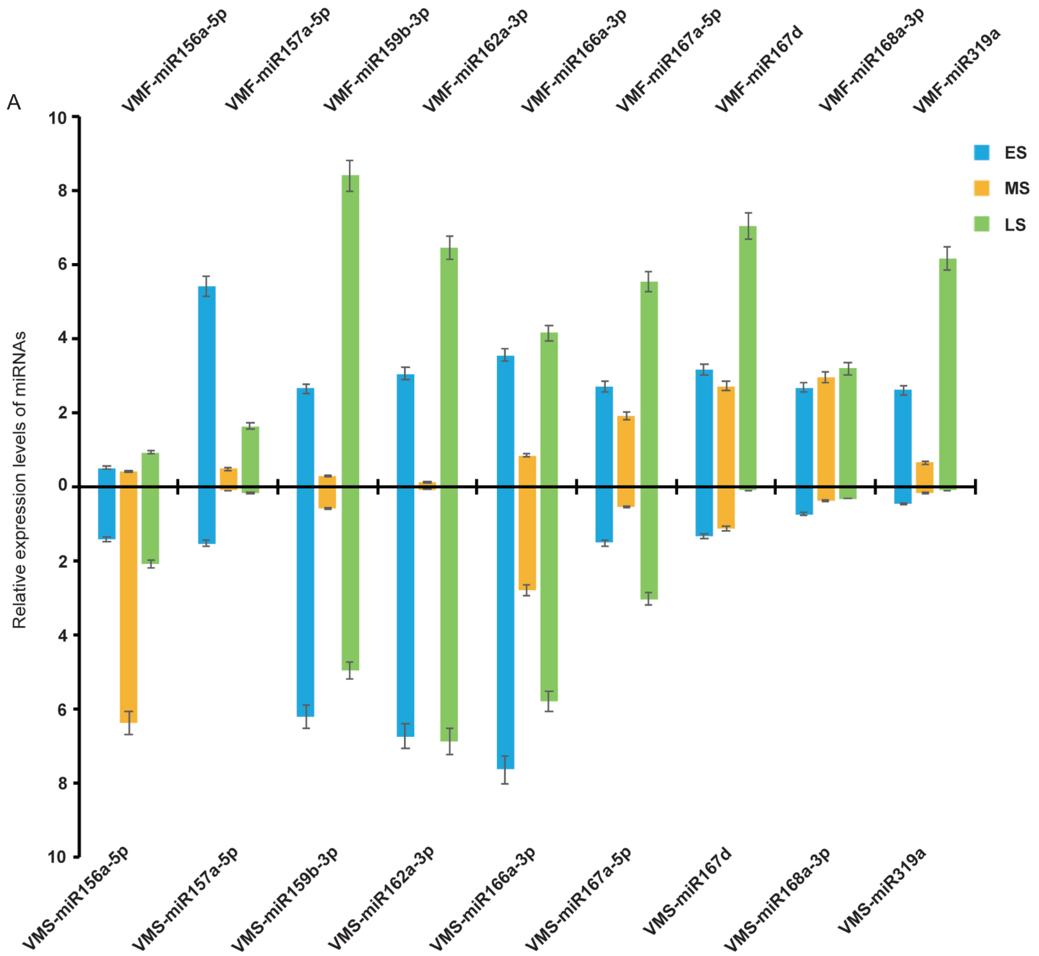

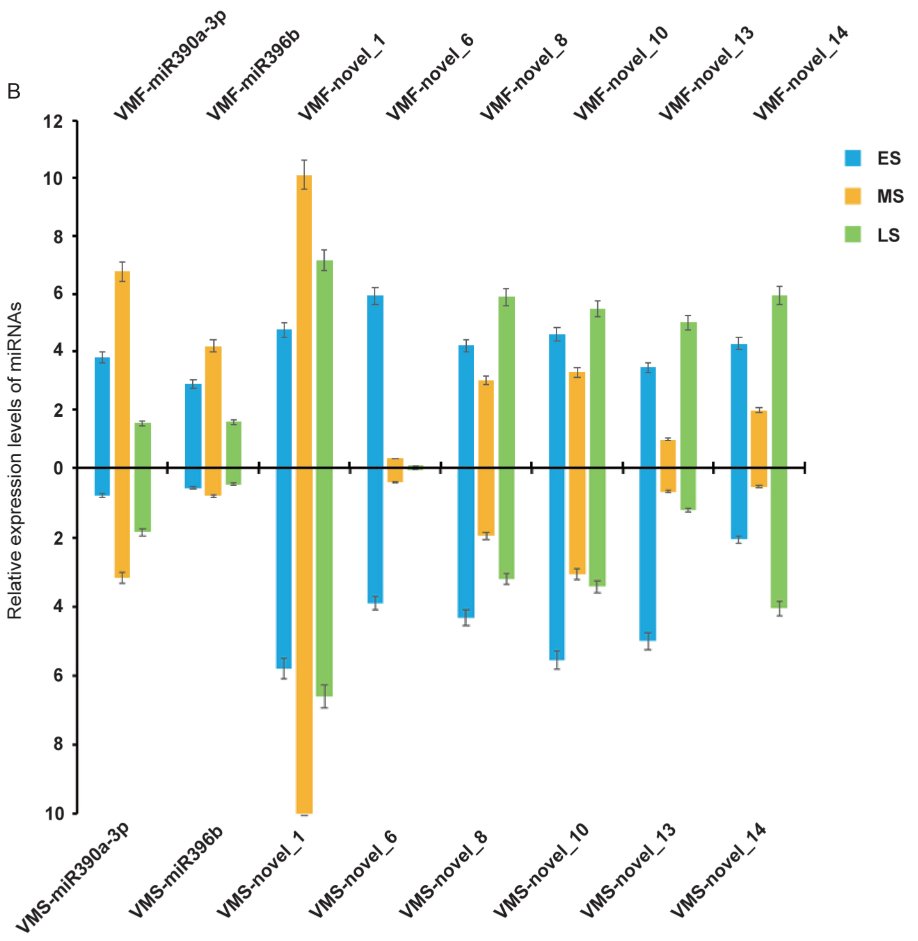

Supplement: Supplementary file 21 — Expression changes of miRNAs at different developmental stages between VMF and VMS. (PDF 2509 kb) [file 12864_2017_4180_MOESM21_ESM.pdf]
