# Supplementary material for: miRNAs involved in the development and differentiation of fertile and sterile flowers in Viburnum macrocephalum f. keteleeri
Source: BMC Genomics. 2017 Oct 13;18:783. doi: 10.1186/s12864-017-4180-x (PMC5640959; doi:10.1186/s12864-017-4180-x)

Additional file 22. Expression changes between miRNAs and targes at different developmental stage.

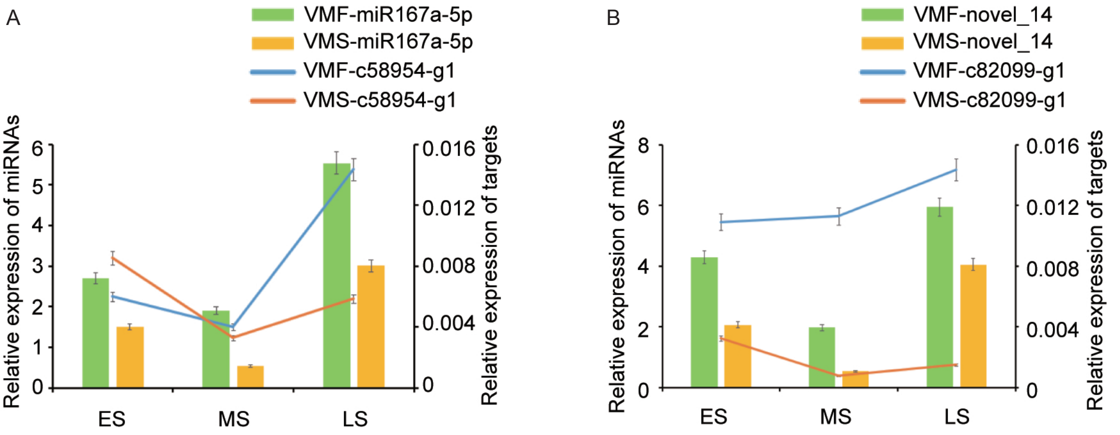

Supplement: Supplementary file 22 — Expression changes between miRNAs and targets at different developmental stages. (PDF 1595 kb) [file 12864_2017_4180_MOESM22_ESM.pdf]
